# Occupational health interventions’ impact on absenteeism and economic returns: A systematic review and meta-analysis

**DOI:** 10.5271/sjweh.4265

**Published:** 2026-03-01

**Authors:** Jonas Backes, Sonja I. Mueller, Alexander Geissler, David Ehlig

**Affiliations:** 1 Chair of Health Economics, Policy, and Management; School of Medicine; University of St. Gallen, St Gallen, Switzerland.

**Keywords:** presenteeism, productivity, sickness absence, sick leave

## Abstract

**Objective:**

Health-related productivity losses impose a significant burden on health systems and economies. Occupational health interventions (OHI) are increasingly promoted as preventive strategies to reduce work-related illness and enhance productivity. However, their effectiveness often remains unclear, creating a lack of guidance to those deciding on their implementation. The aim of this review was to evaluate the effectiveness of OHI in reducing sickness absenteeism and generating economic returns, focusing on mental health, physical health, and workplace atmosphere interventions (eg, work climate enhancement, leadership training).

**Methods:**

A systematic literature search following the Preferred Reporting Items for Systematic Reviews and Meta-Analyses (PRISMA) 2020 guideline was conducted throughout December 2024. Risk of bias was assessed with Joanna Briggs Institute checklists. A random-effects meta-analysis synthesized OHI effects on sick days and return on investment (ROI).

**Results:**

Of 2624 identified studies, 68 across eight industries met eligibility criteria. From these, 23 were included in the meta-analysis: 11 reporting on sick days, and 12 on ROI. OHI were associated with a non-significant reduction in absenteeism [-0.18 days; 95% confidence interval (CI) -2.80–2.43; P=0.890] and a tendency of positive ROI (1.92; 95% CI -0.34–4.17; P=0.096), albeit with statistical uncertainty.

**Conclusion:**

We only found effect of OHI on ROI, however, absence effects on sick days do not necessarily imply a lack of effectiveness. We hypothesize that ROI benefits reflect improvements in presenteeism, although not directly measured. Overall, this review guides OHI selection and implementation, urges standardized evaluation, and prioritizes research on presenteeism measurement, non-OECD settings, and qualitative success factors.

Maintaining workforce productivity is critical for economies and employers globally. Yet, health problems (eg, chronic diseases, mental health conditions, and long COVID-19) cause significant productivity losses (1). Health-related productivity losses can arise from two interrelated phenomena: absenteeism and presenteeism (2).

Health-related absenteeism refers to the absence of employees from work because of health complaints (eg, physical or mental disability) (3). In high-income countries, health-related absenteeism contributes to lost productivity and significant costs. In the United States, 3.3% of full-time workers reduced their hours for health reasons in 2022—the second-highest rate since 1976 (4). In the European Union, health-related absences represent up to 4% of gross domestic product annually (5). Consequently, companies, health insurers, and taxpayers face substantial financial losses, amounting to several billions per year (6).

The rise in health-related absenteeism is driven partly by increasing mental health challenges, emotional exhaustion, and stress-related disorders, now recognized as leading contributors to work disability (6). Additionally, digitalization, infection risks, and social isolation—exacerbated by the COVID-19 pandemic—have intensified physical and psychological pressures on employees (7).

Compounding these challenges, many industries face skilled labor shortages due to an aging population and evolving skill demands, making it harder to replace absent employees. This results in operational bottlenecks and potential declines in service quality (3), creating both short-term productivity losses and long-term challenges in building a resilient, competitive workforce.

Health-related presenteeism refers to the phenomenon where employees continue to work despite being ill or otherwise impaired, often leading to diminished health-related productivity (1). Evidence suggests that the overall productivity loss attributable to presenteeism may surpass that caused by absenteeism. For instance, Li et al (8) estimated that ~40% of the total costs associated with arthritis stem from presenteeism, compared to only ~10% from reduced working hours linked to absenteeism. Similarly, van den Heuvel et al (2) reported that, among individuals with upper extremity disorders, most productivity losses were due to sickness presenteeism (57–89%). Despite its relevance, presenteeism remains difficult to measure, which has likely contributed to its relative underrepresentation in the scientific literature compared to absenteeism (1).

As companies strive to maintain productivity performance, occupational health interventions (OHI) have emerged as a critical strategy for mitigating health issues (9). They are increasingly adopted in clinical, corporate, and governmental settings to improve workforce well-being, reduce absenteeism, presenteeism, and generate economic returns. International organizations, including the World Health Organization (WHO) and the International Labor Organization, have advocated for integrated workplace health programs as part of preventive health strategies (10, 11). However, despite the increasing prioritization of OHI, persistently high absenteeism/presenteeism rates raise the question of which OHI are truly effective for companies and their employees.

Existing reviews often aggregate heterogenous outcome variables (eg, presenteeism, absenteeism, cost savings) or focus narrowly on intervention types, limiting cross intervention comparison (12). Methodological limitations, including small sample sizes and inconsistent designs, have further contributed to inconclusive findings (13). While several reviews examine health-related outcomes (eg, weight loss, stress reduction, improved cardiovascular markers), few have systematically assessed how OHI impact company economics, leaving a critical gap in understanding productivity-related effects. Lastly, OHI are evolving with new technologies and emerging health concerns, leaving earlier syntheses outdated (14).

To address these limitations, the present review systematically compared OHI domains across health-related absenteeism and economic outcomes to identify where interventions yield the greatest value. Our study aimed to assess the effectiveness of OHI in reducing sick-days and creating a positive return on investment (ROI), which is defined as the financial benefit obtained relative to the cost of implementing OHI. Benefits include savings from reduced absenteeism (ie, avoided wage costs for missed workdays) and gains from reduced presenteeism (ie, recovered productivity among employees working while unwell) (15, 16). Thus, a positive ROI reflects not only fewer absences but also improved productivity of employees who perform below their full capacity due to reduced energy, concentration, or work quality (17, 18).

Given rising concerns over mental health, physical inactivity, and workplace atmosphere, which were intensified by the COVID-19 pandemic, this review prioritized OHI targeting those areas. Mental health interventions use problem-solving or stress management programs, while physical health interventions focus on exercise or ergonomics. Workplace atmosphere interventions involve organizational strategies like workshops or team-based communication training to improve communication, teamwork, and psychosocial conditions (19). While safety interventions remain vital, they were excluded from this review as they are typically mandated by national, industry, or company-level regulatory frameworks (eg, the US Occupational Safety and Health Act, the EU Directive 89/391/EEC) and aim to reduce hazards or accidents. Such interventions differ conceptually from voluntary OHI that target behavioral or organizational health improvements, are more flexible in their design choices, and thus limiting comparability.

## Methods

This systematic review and meta-analysis followed the PRISMA 2020 guidelines (20) and was prospectively registered with INPLASY on 15 November 2024 (registration number: 2024110066), two weeks prior to the start of data collection.

### Search strategy and study selection

Studies were included if they met the following criteria: (i) primary quantitative research published in peer-reviewed journals; (ii) experimental, quasi-experimental, observational, or modelling design; (iii) focus on OHI targeting mental health, physical health, nutrition, or workplace atmosphere; (iv) conducted in Organisation for Economic Cooperation and Development (OECD) countries; (v) reported changes in sickness absenteeism or ROI; (vi) involved adult working populations (≥18 years); and (vii) published in English or German between January 2004 and November 2024.

Studies related exclusively to safety, not conducted in workplace settings, or without quantifiable outcomes were excluded. A structured search was performed in PubMed and Web of Science in December 2024 using Boolean operators, tailored to each intervention and outcome type. Full search strings are provided in Illustration 1 of the supplementary material (www.sjweh.fi/article/4265). An unsystematic literature search using Google Scholar supplemented this process to identify gaps missed by structured database searches. Studies from the unsystematic search were directly checked for duplication and verified against the inclusion criteria. Grey literature (eg, dissertations, conference abstracts, organizational reports, preprints, news releases) were not systematically screened.

### Study screening and data collection

Inclusion criteria were applied sequentially as knockout filters. Two independent reviewers screened records using Rayyan software (Rayyan Systems, Inc) (21). Inter-rater reliability was substantial for title and abstract screening (Cohen’s κ=0.66) and almost perfect for full-text screening (Cohen’s κ=0.83). Disagreements were resolved through consensus or by a third reviewer.

Data extraction followed an expanded PICO (Population, Intervention, Comparator, Outcome) framework and included population details, OHI characteristics (eg, type, duration, delivery format), study parameters (eg, sample size, comparators), and outcome data (eg, sick days, ROI ratio). Following Cochrane recommendations, two reviewers jointly extracted effect size data for meta-analysis (22). When several publications utilized the same dataset, we treated each independently in the meta-analyses but counted each set once for overarching descriptives (eg, total participants, mean age, female ratio) to avoid double-counting. Details on data extraction parameters can be found in supplementary table S1.

### Risk of bias assessment following the Joanna Briggs Institute (JBI)

The latest JBI critical appraisal checklists endorsed by Cochrane were used to assess risk of bias (22). Papers were evaluated using the appropriate checklist based on their study type (ie, RCT, quasi-experimental, and cohort study). Studies with an economic outcome underwent an additional assessment using the JBI Economic Evaluations Checklist, implying that ROI-focused studies received two JBI based appraisals.

The checklists were modified by removing two questions on treatment blinding and group equivalence given their impracticality in real-world workplace settings. Two researchers were involved in the risk of bias assessments, with disagreements resolved through consensus. The exact methodology for each appraisal tool is presented in supplementary table S2.

In line with previous research (23), studies were categorized according to the JBI rating as low (>0.7), moderate (0.5–0.7), or high risk of bias (<0.5). Only low-risk studies were included in the primary meta-analysis; others were retained for subgroup analysis.

### Calculation of meta-effects

Meta-analytic synthesis was conducted using random-effects models implemented in R (version 4.4.1). For absenteeism outcomes, mean differences in sick days between intervention and control groups were extracted directly or derived from study data. When reported for periods other than 12 months, results were standardized using linear time-scaling. Confidence intervals (CI) for studies with differing timeframes were adjusted by scaling the standard error (SE) proportionally to the square root of the ratio between the new and original periods:

$${\mathrm{CI}}_{T}\text{=}\mu_{T}\pm Z\cdot {\mathrm{SE}}_{t}\cdot \sqrt{\frac{T}{t}}$$

where *μ* represents the estimate’s mean, *Z* the Z-score for the desired confidence level, and ​*SE_t_* the standard error. *T* and *t* refer to the target and original time periods, respectively. The factor $\sqrt{\frac{T}{t}}$ scales the error for the target period *T* and follows the assumption that the variance of cumulative outcomes (ie, sick days) increases linearly with time (see illustration 2 of the supplementary material for detailed derivation).

ROI values were retained in reported timeframes to avoid time-scaling bias. Studies without CI reporting or alternative outcome measures (eg, departmental-level absenteeism or cost-effectiveness ratios) were excluded due to non-comparability but remain part of the discussion.

Between-study variance τ*^2^* was estimated using the restricted maximum likelihood (REML) method (24), which is robust for meta-analyses with varying heterogeneity and study sizes (25). When REML produced excessive weight concentration in a small number of studies, we instead applied the Sidik-Jonkman (SJ) method. The SJ method was preferred in these cases because it is less sensitive to very small within-study variance, limits undue influences of single studies, and distributes weights more evenly across studies. The choice of the method was made after inspecting the weights.

We conducted sensitivity analyses, including funnel plots and leave-one-out analyses, for both primary (JBI rating >0.7) and extended analyses (all studies) on sick days and ROI. Due to few studies, results should be interpreted cautiously. Subgroup analyses were conducted based on intervention domain and type, industry, and delivery format to compare relative effectiveness and economic efficiency.

## Results

### Included studies and characteristics

**Figure 1:**
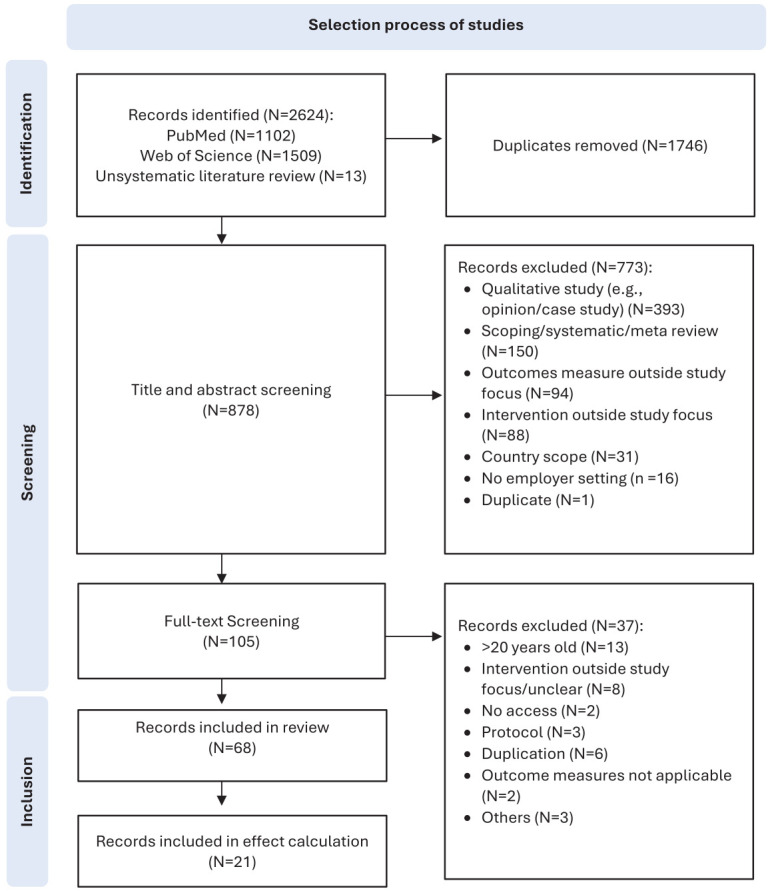
PRISMA flow chart of paper selection process.

The PRISMA flow diagram (figure 1) illustrates the study selection process. After screening 2624 records, 68 studies met the inclusion criteria (26–93) (table 1). Detailed search data by intervention type, outcome measure, and database can be extracted from supplementary figure S1, and the full-text screening decisions in supplementary table S3.

**Table 1:** Summary characteristics of papers included in the review (N=68). More detailed study vignettes can be investigated in table 4 of the supplementary material. [N/A=not available; Quasi-E=quasi experiment; RCT=randomized control trial; ROI=return on investment.]

Reference ^1^	Study design (size)	Study population	Intervention	Control	Outcome ^2^	Delivery format ^3^	Country scope
Akerstrom et al (26)	Quasi-E (N=N/A)	Public sector employees, ~85% in healthcare	24-month stress intervention led by line managers and Human Resources, focused on task redesign	Reference data from 247 matched workplace groups	Sickness absence rate	Phone, In-person	Sweden
Arends et al (27)	RCT (N=158)	Employees (18–63) with Common Mental Disorders at start of sick leave	Problem-solving by occupational physicians (2–5 consults) following SHARP-at-work model	Single consultation per Dutch occupational guidelines	Recurrent sick leave (incidence/time)	In-person	Netherlands
Baker et al (28)	Quasi-E (N=890)	US employees with obesity or comorbid conditions	Weight loss and health improvement program (up to 48 sessions and health tracker/tools)	No comparator	ROI	Phone, Digital	USA
Blake et al (29)	Quasi-E (N=1452)	National Health Service (NHS) hospital staff	5-year wellness program including health campaigns, fitness, and relaxation	No comparator	Sickness absence rate	Digital, In-person	UK
Blangsted et al (30)	RCT (N=616)	Danish office workers	Resistance training and general physical exercise with contracts and trainer visits	No additional physical activity	Sick days; sickness absence rate	In-person	Denmark
Bondar et al (31)	Quasi-E (N=1,132)	US employees with moderate anxiety or depression	Digital mental health program (screening, self-help, care access)	No comparator	ROI	Digital, In-person	USA
Braun et al (32)	Quasi-E (N=N/A)	UK employees in Better Health at Work Award program	Tiered health program focusing on workplace improvements and health promotion (e.g., nutrition)	No comparator	Sick days; cost-benefit	Not provided	UK
Brox & Frøystein (33)	RCT (N=129)	Norwegian care home staff	Weekly light aerobic group exercise and optional stress/nutrition education	No intervention	Sick days	In-person	Norway
De Boer et al (34)*	RCT (N=116)	Employees >50 years, reporting inability to work until retirement	Occupational health program: 3+ consultations, action plan, referrals as needed	Care as usual (access to doctor, no invite to consult)	Sick days	In-person	Netherlands
Dement et al (35) *	Cohort (N=10,432)	University hospital staff	Long-term workplace health program including assessments, coaching, wellness access	No program participation	ROI	Digital, Phone, In-person	USA
Duijts et al (36)	RCT (N=151)	Employees at high risk of sickness absence due to psychosocial health (not on leave)	Preventive coaching (7–9 sessions over 4.5 months) led by trained coaches	Care as usual (e.g. social worker or company doctor counselling if needed)	Sick days	In-person	Netherlands
Ebert et al (37)*	RCT (N=264)	German employees with high stress (PSS-10 ≥22)	2.5-month digital stress management training with e-coach focused on problem-solving and emotion regulation	Waitlist control with care as usual	ROI; net benefit; cost-effectiveness ratio; cost-benefit ratio	Digital	Germany
Elson et al (38)	Modeling (N=1,984)	Education department employees in South Australia	Employee Assistance Program with 6 counseling sessions	No comparator	Cost-benefit ratio	Digital, Phone, In-person	Australia
Framke et al (39)*	RCT (N=2,576)	Preschool staff in 78 Danish schools	25-month participatory work climate intervention (consisting of 4 phases)	Staff in 34 preschools with no intervention	Sick days	In-person	Denmark
Freund et al (40)*	RCT (N=396)	German employees	Digital stress program with modules on psychoeducation, problem-solving, emotion regulation, and future planning	Waitlist control	ROI; net benefit; cost-benefit analysis; cost-effectiveness ratio; cost-utility	Digital	Germany
Geraedts et al (41)	RCT (N=231)	Dutch employees with depressive symptoms	6-week guided online self-help combining cognitive behavioral therapy and problem-solving (weekly lessons)	Care as usual	ROI; net benefit; cost-benefit ratio; cost-effectiveness ratio; cost-utility	Digital	Netherlands
Goetzel et al (42)	Quasi-E (N=2,458)	Employees from small businesses in Colorado	Health Risk Management program, including annual health assessments, action reports, coaching, self-learning	No comparator	ROI	Digital, Phone, In-person	USA
Gregson et al (43)	Quasi-E (N=80)	Danish hospital staff	3-month fitness training (2×30min/week)	Non-participants in same unit	Sickness absence hours	In-person	Denmark
Groeneveld et al (44)	RCT (N=573)	Construction workers with cardiovascular disease risk	Lifestyle intervention including coaching (face and phone) and brochures on activity, diet, and cardiovascular disease	Care as usual	Cost savings	In-person, Phone	Netherlands
Gubler et al* (45)	Quasi-E (N=111)	US industrial laundry workers	Free wellness program with biometric screenings, reports, seminars, insurance discount	Employees at other site	ROI	In-person	USA
Hendriksen et al (46)	Quasi-E (N=433)	Office staff at Dutch insurance company	5-month multilevel program including leadership training and health coaching	No comparator	Sickness absence rate	Digital, Phone, In-person	Netherlands
Hengel et al (47)	RCT (N=293)	Construction workers from 15 departments of 6 Dutch companies	Physical training and empowerment sessions (6 months)	No intervention	Sick days (dichotomized)	In-person	Netherlands
Hengel et al* (48)	RCT (N=293)	Construction workers from 6 Dutch companies	6-month physical and mental training at worksites	Mandatory safety training only	Sick days; ROI	In-person	Netherlands
Herman et al (49)	RCT (N=70)	Workers with chronic low back pain	Naturopathic treatment incl. acupuncture, physical exercise, nutrition	Standardized physiotherapy education and a brochure	ROI	In-person	Not provided
Hughes et al (50)	Quasi-E (N=516)	Overweight employees in three US Fortune 500 companies	Employer-led weight management with physical activity, diet, medical monitoring	No comparator	Sick days	In-person	USA
Iijima et al (51)	Quasi-E (N=12,864)	Employees from 11 Japanese companies	Tiered mental health prevention per national guidelines	Companies grouped by ROI	ROI; net benefit	Not provided	Japan
Ikegami et al (52)	Quasi-E (N=1,420)	Japanese manufacturing employees	Manager training in active listening and feedback session	Pre-intervention baseline	Sickness absence rate	In-person	Japan
Jenny et al (53)	Quasi-E (N=5,014)	Swiss employees from 8 diverse pilot companies	Multifaceted stress management incl. self-assessment, leadership training, and formation of collaborative groups	Non-participants in stress training program	ROI	Digital, In-person	Switzerland
Jorgensen et al (54)	RCT (N=363)	Danish cleaning staff	Physical coordination or cognitive behavioral training with group sessions	Health check with fitness test	Sick days	Phone	Denmark
Kapinos et al (55)	Cohort (N=20,251)	US employees at chronic disease risk	Health risk assessment (20 min) and tailored or intensified lifestyle coaching	No intervention	Cost savings	Digital, Phone, In-person	USA
Karlsson et al (56)	RCT (N=197)	Adults (18–59) on short-term sick leave for common mental disorders	Five-step problem-solving approach in 2–5 sessions	Care as usual (i.e., cognitive behavioral therapy or antidepressants)	Sick days	In-person, Phone	Sweden
Keus van de Poll et al (57)	RCT (N=100)	Employees with common mental disorders or stress symptoms	Occupational Health Service consultants with 1-day training in problem-solving; 3 follow-ups over three months	Occupational Health Service consultants with brief intro to psychosocial/mental health	Sick days	In-person	Sweden
Keus van de Poll et al * (58)	RCT (N=100)	Employees with common mental disorders or stress symptoms	Occupational Health Service consultants with 1-day training in problem-solving; 3 follow-ups over three months	Occupational Health Service consultants with brief intro to psychosocial/mental health	Net benefit; cost-benefit; cost-effectiveness ratio	In-person	Sweden
Klasen et al* (59)	RCT (N=139)	Employees at high risk of long-term sickness absence with mild depressive symptoms	Psychological treatment (problem-solving therapy and cognitive behavioral therapy; up to 13 sessions)	Care as usual on request	Sick days	In-person	Netherlands
Kobayashi et al (60)	Controlled trial (N=1,070)	Office workers in Japanese steel manufacturer (non-shift departments)	Participatory program to improve work environment using Mental Health Action Checklist	No intervention	Sick days (dichotomized)	In-person	Japan
Krampen et al (61)	RCT (N=56)	Public service employees selected for high absenteeism	2-month systematic self-observation and reflection on health behavior (weekly meeting in small groups)	Waiting list control group	Sick days	In-person	Germany
Larsen et al* (62)	Quasi-E (N=405)	Danish nursing home staff	Health literacy training and structured manager-staff dialogues	Same employees but pre-intervention	Sick days	Not provided	Denmark
Lerner et al* (63)	RCT (N=253)	US veterans with work-restricting depression	“Be Well at Work” phone counseling and integrated care (IC)	IC without work counseling	ROI	Phone	USA
Linden et al (64)	Quasi-E (N=509)	Employees of German Federal Pension Insurance department	Health program including courses (nutrition, stress, fitness) and team focus groups	Departments not participating in the intervention	Sickness absence rate	In-person	Germany
Losina et al (65)	Cohort (N=292)	US office employees (insurance)	6-month team-based physical activity challenge with financial incentives	Compared groups based on weekly activity levels	Sickness absence hours	Digital, In-person	USA
Makrides et al (66)	Cohort (N=402)	Public Service employees in Nova Scotia Department of Justice	Comprehensive wellness program including risk assessment, lifestyle coaching, workplace changes	No comparator	Sick days (dichotomized)	In-person, Phone, Digital	Canada
Meenan et al (67)	RCT (N=6,958)	Overweight hotel workers in Hawaii	2-year weight loss program with assessments, feedback, support groups	Minimal intervention	Net present value	In-person	USA
Milani et al (68)	RCT (N=339)	Employees and spouses of one employer	6-month wellness program Risk Assessment and Modification Program covering fitness, stress, and health access	Care as usual	ROI; cost savings	In-person	USA
Musich et al (69)	Quasi-E (N=24,651)	US Dell employees with insurance coverage	Health risk assessments, lifestyle coaching, disease management support	Non-participants	ROI; net benefit	Digital, Phone, In-person	USA
Noben et al* (70)	RCT (N=413)	Dutch hospital nurses at mental health risk	Mental health screening, referral and physician-led improvement protocol	Screening only	ROI; net benefit; cost-benefit assessment	Digital, In-person	Netherlands
Norwitz et al (71)	Quasi-E (N=9)	Employees with obesity, prediabetes, or diabetes	Ketogenic diet, virtual check-ins, glucose monitoring, app support	No comparator	Cost savings	Digital	USA
Ornek et al (72)	Quasi-E (N=70)	Turkish female textile workers with work-related stress	45-minute sessions on stress management techniques, coping strategies, relaxation exercises (12 weeks)	Women at another factory	Sickness absence hours	Digital, Phone, In-person	Turkey
Proper et al (73)	RCT (N=299)	Dutch civil service office workers	Seven physical activity counselling sessions (20 min.) during working hours	General written health information	Cost-benefit assessment; cost-effectiveness ratio	In-person	Netherlands
Rantonen et al* (74)	Quasi-E (N=193)	Workers with lumbar spine pain	Rehab, physiotherapy, or counseling for 1–5 months	Care as usual	Sick days	In-person	Finland
Renaud et al (75)	Quasi-E (N=270)	Canadian financial sector employees	3-year multi-module wellness program on stress management, heart health, nutrition, and physical activity	No comparator	Sickness absence rate	Phone, In-person	Canada
Robroek et al (76)	RCT (N=924)	Employees in Dutch healthcare/logistics/public sectors	Digital wellness program with counseling and online self-monitoring tools	Health check with personal advice and feedback via a website	Sickness absence rate	Digital, In-person	Netherlands
Ryan et al (77)	Pre-post design (N=1,400)	Australian hospital employees	Daily 6-min “take-a-break” group sessions on posture, relaxation, education	3-year pre-program injury data	Sick days; cost savings	In-person	Australia
Saleh et al (78)	Quasi-E (N=151)	Rural US employees from six organizations	Health risk assessment and awareness; with/without lifestyle coaching	No intervention	Cost-effectiveness ratio	Not provided	USA
Song et al (79)	RCT (N=32 974)	US warehouse workers	Multi-component wellness program (12 modules on nutrition, exercise, stress reduction)	No intervention	Sickness absence rate	Not provided	USA
Stansfeld et al* (80)	RCT – pilot (N=350)	Staff and managers from NHS mental health services, excluding those on long-term sick leave	Guided e-learning on stress for managers (6 modules over 3 months)	Managers in another service with no intervention	Sick days	In-person	UK
Taimela et al (81)	RCT (N=382)	Workers with high risk of sickness absence	Nurse/physicians-led program including feedback on risk assessment, action plan, referrals	Contact on request, no feedback or consult	Sick days	In-person	Finland
Taimela et al (82)	RCT (N=382)	Employees at high risk of sickness absence (based on self-reported work ability, fatigue, etc.)	Nurse/physician-led feedback, consultation offer, action plan, referrals if needed	No feedback on health results; no invitation to consult; could request doctor/nurse contact	Sick days	In-person	Finland
Thiart et al* (83)	RCT (N=128)	Teachers with insomnia	Internet-based cognitive behavioral therapy for insomnia with weekly modules and e-coaching	Waitlist control	ROI; net benefit; cost-benefit ratio	Digital	Germany
Tveito & Eriksen (84)	RCT (N=40)	Nursing staff in a care home for the elderly	9-month health program: exercise (1hr/week); lifestyle education, stress sessions (15hrs)	Delayed intervention after pre/post-tests	Sick days	In-person	Norway
van den Ven et al (85)	Quasi-E (N=176)	Hospital and manufacturing workers with low- socio-economic position	Preventive medical check-up and 6-month motivational interviewing (up to 7 sessions)	Preventive medical check-up only	Sick days (dichotomized)	Digital, In-person	Netherlands
van Dongen et al* (86)	RCT (N=730)	Older Dutch hospital employees	Vitality exercise including yoga session, unsupervised exercise training, and three coaching sessions	Written information about a healthy lifestyle	ROI; net benefit; benefit-cost ratio	In-person	Netherlands
van Dongen et al* (87)	RCT (N=412)	Office employees of a financial services provider	9-min. motivational group interviews (3 sessions) and “VIP zones” to improve social and physical environments	Care as usual	ROI; net benefit; cost-effectiveness ratio; cost-benefit-ratio	Digital, In-person	Netherlands
van Holland et al (88)	RCT (N=303)	Meat processing workers (≥12 hrs/week)	Online questionnaire and personalized intervention recommendations (e.g., General practitioner or dietician visits)	Routine occupational healthcare and existing programs	ROI; net benefit; cost-benefit ratio	In-person, Digital	Netherlands
Viester et al* (89)	RCT (N=314)	Construction workers with prior health check-ups	6-month health promotion program on activity and diet	Care as usual	Sick days	Phone, In-person	Netherlands
Von Thiele Schwarz et al (90)	Quasi-E (N=1311)	Public dental health employees	12-month reduced work hours or physical exercise at work	No intervention	Sick days	In-person	Sweden
Vonderlin et al* (91)	Quasi-E (N=1830)	Managers and employees from 12 companies	6-month mindfulness and leadership training for managers	No intervention	Sick days	In-person	Germany
Wijnen et al* (92)	Cohort study (N=303)	Healthcare workers, mainly carers	Digital platform helping to assess needs, select interventions, and evaluate effectiveness	Waitlist control	ROI; net benefit; cost benefit	Digital	Netherlands
Zetterberg et al* (93)	RCT (N=147)	Public sector workers with pain/stress-related illnesses (majority women)	“Effective Communication within the Organization” program: 2–3 hybrid group sessions (skill training, reflection)	Psychoeducation (2 lectures and brochures)	Sick days	Digital, In-person	Sweden

^1^ Studies with stars are included in the meta effect calculation. ^2^ Only outcomes measures listed relevant for this review (eg, multiple studies record health related outcome measures which are not listed here). ^3^ Digital=web/app-based, phone=phone-based.

The studies represented ~160 000 participants with a mean age of 40.3 years and 45.5% female participation. Samples sizes within each study ranged from 9 (71) to 48 664 (79), with a mean of 2388. The majority were conducted in The Netherlands (26%), the United States (22%), Germany (9%), and Scandinavia (24%). The articles spanned various sectors: the majority studied healthcare settings (31%), including hospitals and long-term care, and industrial sectors (22%), such as metal and construction. OHI primarily targeted individual employees (90%), with fewer addressing leadership (19%), organizations (13%), or teams (7%). Interventions were mainly delivered in-person (79%), with digital (38%) and phone (25%) formats used less frequently. Hybrid methods combining in-person, phone, and digital approaches were used in 10% of studies. Delivery formats shifted over time, with digital and hybrid OHI increasing from 2015 onward. Recent years (2019–2024) saw a consistent increase in hybrid models (supplementary figure S2).

**Figure 2:**
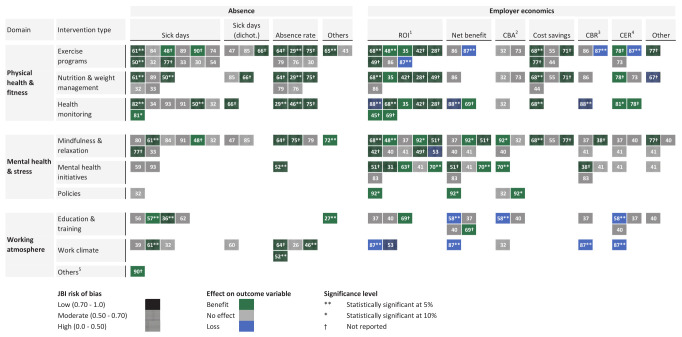
Overview of study results and risk of bias for selected studies based on outcome measure and OHI domain. Note: Study numbers correspond to the reference number and numbering of Table 4 of the Supplement Material, with some studies appearing multiple times as they evaluate various interventions and outcomes. Shading pattern reflects the JBI rating: fully shaded for low (JBI>0.7), dotted for moderate (0.5–0.7), and striped for high risk of bias (<0.5). Colors indicate effect direction: black for benefits, dark gray for no effect, and light grey for negative outcomes. Non-significant results are classified as no effect. Stars indicate statistical significance levels reported for outcome variable in respective study. Crosses represent studies without reported significance levels. 1) Return on investment. 2) Cost-benefit analysis. 3) Cost-benefit ratio. 4) Cost-effectiveness ratio. 5) Eg, Reduced work hours

Interventions were categorized into three overarching OHI domains (figure 2) with 34% focusing on physical health and fitness, 16% on mental health, 13% on workplace atmosphere, and 37% covering multiple domains. Each domain included various intervention types. The most common being exercise programs (50%), mindfulness or relaxation (37%), and nutrition or weight management (35%). Regarding outcome variables, most of the included studies focused on the impact of OHI on sickness absenteeism (53%), followed by economic variables (43%), with a small proportion (4%) addressing both. .

Vignettes for each paper included in the review (N=68) and summary statistics can be found in supplementary tables S4 and S5.

### Risk of bias assessment

Of the 68 studies, 35 (51%) were classified as low, 19 (28%) as moderate, and 14 (21%) as high risk of bias. The most common biases in moderate-to-high-risk studies included missing statistical analyses (16 studies), no control group (15), and unreliable outcome measurements (14).

Of the 33 studies that underwent an economic evaluation appraisal, over 70% were rated as low risk of bias, while 9 (~27%) were classified as having moderate-to-high risk of bias. The main deficits for economic studies included missing information on generalizability (9 studies), insufficient intervention or comparator description (8 studies), and no sensitivity analyses (7 studies). The JBI critical appraisal results are presented in supplementary table S6.

### OHI effects on absence and employer economics

Figure 2 provides an overview of the two primary outcome dimensions, absenteeism and employer economics, mapped to their respective interventions, risk of bias assessment, and statistical effect.

Approximately 25% of studies reported a statistically significant positive effect. Among these, around three-quarters focused on absences, while the remaining addressed economic outcomes. The strongest individual effects among low-risk studies were observed in a stress intervention by van de Poll et al (57) reducing absenteeism by 15 days annually, and a mental health program for nurses by Noben et al (70), reporting an ROI of €11 per euro invested.

Negative effects were found in only 5% of studies, all related to ROI. The study with the largest loss (€6.66 per euro invested) was conducted by van Dongen et al (87), targeting the social and physical work environment of office employees in a financial services organization. Notably, 90% of the studies indicated a positive trend for either sickness-related or economic outcomes. A summary by sector (healthcare, industrial, consumer services) is provided in supplementary figure S3.

### Meta-analyses

Meta-analyses were performed to assess the effect of OHI on sick days and ROI.

*Meta-analyses of sick days for low risk of bias studies*. Using REML estimation, OHI were associated with a non-significant mean reduction of -0.39 sick days per employee annually (95% CI -0.89–0.10; P=0.121), with low heterogeneity and between-study variance (I^2^=0.00%; τ^2^=0.00). Yet, the results are primarily driven by Larsen et al (62), which accounted for 95% of the weight in the random-effects model (supplementary figure S4). This study reported a general reduction in sick days of -0.44 (95% CI -0.95–0.07) scaled up from an original six-month period.

**Figure 3:**
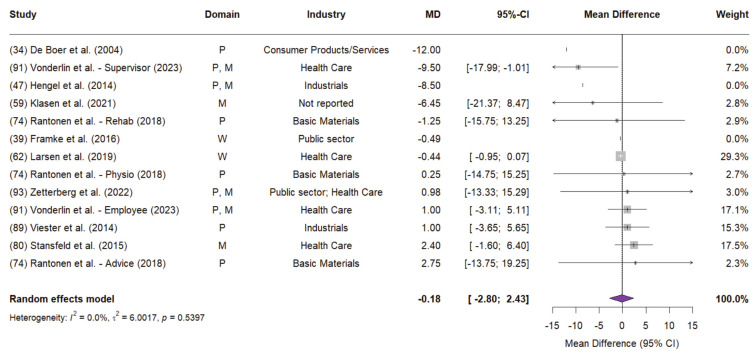
Random-effects model (SJ method) for mean differences = in sick days in low risk of bias studies (JBI > 0.7) Note: Study numbers correspond to reference number and numbering of Table 4 of the Supplement Material. For the intervention domain, the following abbreviations apply: P=Physical health and fitness; M=Mental health and stress; W=Education and training. Studies that did not report statistical results for the mean difference in sick days were excluded from the random-effects calculation (assigned a weight of 0%) and are displayed for reference purposes only. Some studies report sick day results for specific sub-groups (eg, Vonderlin et al. (91), hence they appear multiple times in the forest plot with the respective subgroup designation. The P value at the lower end of the forest diagram refers to the heterogeneity test.

To correct for the excessive weight of the study, a follow-up analysis using the SJ method for heterogeneity estimation was conducted (figure 3). The SJ method yielded a mean difference of -0.18 days (95% CI -2.80–2.43; P=0.890), with a wider CI than the REML model. The heterogeneity and between-study variance estimates remained low (I^2^=0.00%; τ^2^=6.00). The distribution of study weights was more balanced in the SJ model, reducing the dominance of Larsen et al (62), which accounted for 29.3% of the total weight compared to 95% under REML. Despite this adjustment, the overall findings remain statistically non-significant.

Including all studies regardless of risk of bias increased heterogeneity and between-study variance substantially (I^2^=96.1%; τ^2^=105.11). Sick days were reduced to -3.99 (95% CI -10.18–2.21; P=0.207), but results were notably influenced by one high risk of bias outlier [ie, Krampen et al (61)]. The variability in mean differences ranges from -32.4–8.06 sick days.

**Figure 4:**
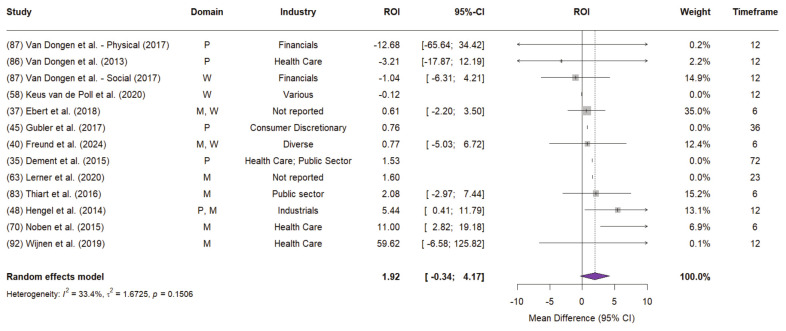
Random-effects model (REML method) for ROI point estimate from low risk of bias studies (JBI>0.7) Note: Study numbers correspond to reference number and numbering of Table 4 of the Supplement Material. For the intervention domain, the following abbreviations apply: P=Physical health and fitness; M=Mental health and stress; W=Education and training. Studies that did not report statistical results for the ROI point estimate were excluded from the random-effects calculation (assigned a weight of 0%) and are displayed for reference purposes only. Timeframe indicates the horizon over which the ROI was calculated. The P value at the lower end of the forest diagram refers to the heterogeneity test.

*Meta-analyses of ROI for low risk of bias studies.* The random-effects model for ROI outcomes in low risk of bias studies yielded an ROI of 1.92 (95% CI -0.34–4.17; P=0.096), with moderate heterogeneity (I^2^=33.4%; τ^2^=1.67) (figure 4). The most precise study, a randomized controlled trial (N=264) by Ebert et al (37) on a digital stress management program, reported an ROI of €0.61 per euro invested over six months. Its modest effect size was offset by high model weight from narrow confidence intervals. ROI estimates ranged widely, with van Dongen et al (87) reporting -€12.68, and Wijnen et al (92) reporting €59.62, driven by €2981 in productivity gains per employee. Due to wide CI, both studies received relatively low weight in the random-effects model.

When including all studies regardless of risk of bias ROI estimate decreased to 1.05 (95% CI- 2.21–4.30; P=0.529), mainly driven by Geraedts et al (41) and van Holland et al (88), both with moderate and high risk of bias. Heterogeneity increased substantially (I^2^=74.00%; τ^2^=14.22).

Funnel plots for low-risk of bias studies appeared broadly symmetrical for both sick days and ROI outcomes, though interpretations should be cautious due to the small number of studies. Leave-one-out analyses showed robust findings, with no single study substantially altering the primary meta-analyses.

*Subgroup analysis.* All subgroup analyses were restricted to studies with a low risk of bias and conducted by intervention domain, intervention type, industry, and delivery format. Due to small sample sizes and lack of significant results in some subgroups, the following analyses are exploratory and require further validation.

A closer examination of the intervention domains and types driving economic effectiveness reveals that mental health and stress interventions (k=7 studies) show a statistically significant positive impact on ROI, with a point estimate of 2.99 (95% CI 0.02–5.96; P=0.049; I^2^=50.00%; τ^2^=4.50), providing evidence of an economic benefit. In contrast, the effect on absenteeism (k=6) was inconclusive (mean difference -1.02; 95% CI -5.70–3.67; P=0.579; I^2^=43.60%; τ^2^=13.16). Over 85% of intervention types contained fewer than four studies. Hence, no other intervention domains or types showed significant effects on sickness absenteeism or ROI, with most exhibiting substantial heterogeneity.

Subgroup analyses by industry showed no statistically significant effects on absenteeism. In healthcare (k=4) the ROI showed a non-significant positive effect of 7.49 (95% CI -6.27–21.24; P=0.144; I^2^=59.80%; τ^2^=68.59). Other industries had minimal or no available data, limiting meaningful comparisons.

Lastly, subgroup analysis by delivery format revealed no statistically significant effects on absenteeism or ROI. In-person interventions (k=3) showed the highest ROI (4.03; 95% CI -2.24–10.29; P=0.074; I2=10.10%; τ^2^=3.79), while digital-only interventions (k=4) showed lower and more uncertain ROI values (0.99; 95% CI -1.31–3.29; P=0.264; I^2^=7.80%; τ^2^=<0.01). Full estimates of the subgroup analyses are reported in supplemtnary figure S6.

*Heterogeneity across studies.* Meta-analyses of low-risk studies showed low heterogeneity, but including all studies increased heterogeneity significantly (I^2^=96.10% for sick days, I^2^=74.00% for ROI), mainly due to outliers like Krampen (61). Adding the study by Krampen to the low-risk group raised heterogeneity to I^2^=96.9%. Heterogeneity also changed in other subgroup analyses. Mental health and stress interventions had the highest variability (I^2^=43.60% for sick days; I^2^=50.00% for ROI). Healthcare industry studies also showed significant heterogeneity (I^2^=41.20% for sick days; I^2^=59.80% for ROI), likely due to diverse organizational settings and workforce structures.

## Discussion

This study examined whether OHI reduce sickness absenteeism and generate economic returns. Most prior reviews have focused narrowly on specific intervention domains or workforce subgroups (17, 94–96), limiting cross-domain comparisons. This review is among the first to systematically evaluate and compare the effectiveness of major OHI domains across both absenteeism and ROI outcomes.

Meta-analysis using the SJ method yielded a mean difference in absenteeism of -0.18 days (95% CI -2.80–2.43; P=0.890), indicating no measurable effect. The pooled ROI was 1.92 (95% CI -0.34–4.17; P=0.096), indicating a tendency for an economic benefit. However, the CI points to uncertainty around the estimate. At the same time, the upper bound of the interval suggests that, if effective, OHI may yield substantial economic returns.

Mental health and stress interventions were the only domain with a statistically significant ROI.

Although our analysis did not show a statistically significant reduction in sick days, multiple studies within our sample demonstrated significant effects. Van de Poll et al (57) reported a 15-day reduction in sick leave over 12 months, Hughes et al (50) observed a drop in self-reported sick days from 3.1 to 1.1 within 3 months, and Ornek & Esin (72) found significant decreases in absenteeism duration alongside enhanced job performance. Authors argue effects were often fueled by adequate resource provision (both monetary and non-monetary), strong managerial involvement, active employee participation, clear communication, and an open feedback culture.

The economic advantage of mental health interventions aligns with prior evidence linking improved presenteeism to reduced productivity loss from depression, anxiety, and chronic stress (97–99). Prior studies suggest that presenteeism may account for greater economic burden than absenteeism (100, 101), and interventions that improve mental health have shown consistent potential to offset these losses.

Findings should be interpreted in light of study quality and heterogeneity. While primary analyses showed low heterogeneity (I^2^=0% for sick days; 33% for ROI), some subgroups varied substantially. This heterogeneity arises, for instance, from differences in study design (eg, RCT versus quasi experimental), target populations (sick-listed, high-risk, or general employees), OHI level (individual, group or organizational), and outcome definitions (registry- versus self-reported absence).

Many of these parameters can be standardized to enhance comparability and quality. For instance, standards could specify the cost components included in ROI calculations or establish registry-based reporting (rather than self-reported data) as the preferred approach. With only half of our included studies rated with low risk of bias, developing and adopting these standards is essential.

### Presenteeism: The hidden burden

The absence of measurable change in sickness absence does not necessarily imply that employees are maintaining their health level. Gradual changes, for example in presenteeism, often remain undetected; some surveys suggest up to ~80-90% of employees occasionally work while ill (102, 103). Habitual presenteeism can have serious implications for health systems, as it may prolong illness, delay recovery, and increase vulnerability to future health conditions, thereby consuming additional healthcare resources (104). We hypothesize that the positive ROI in combination with inconclusive effects for sick day reduction might capture potential gains in on-the-job productivity driven by a reduction in presenteeism (105). This explanation is theoretical as this study did not directly extract outcome indicators for presenteeism.

Several studies in our sample identified presenteeism as the primary driver of cost reductions and positive ROI. For example, Thiart et al (83) evaluated an internet-based cognitive behavioral therapy for insomnia and found that savings from reduced presenteeism (€540 per participant) far exceeded those from absenteeism (€78), resulting in an ROI of 208%. Similarly, Ebert et al (37) reported that the economic benefit of a digital stress-management program (ROI €0.61) was largely attributable to improvements in on-the-job productivity rather than reduced sick leave. Thus, including presenteeism metrics can substantially change the estimated economic value of OHI. Ignoring these productivity effects may underestimate the true benefits.

Including presenteeism costs in economic evaluations has become increasingly common despite debates about complexity and the heterogeneity of measurement (1, 106). In our sample, we identified three methods for estimating presenteeism effects: (i) quantifying lost workdays (eg, Osterhaus method) (37, 40, 70, 83, 92), (ii) assessing overall performance during working hours (eg, WHO Health and Work Performance Questionnaire [WHO-HPQ]) (48, 87), and (iii) evaluating health-related functional limitations (eg, Work Limitations Questionnaire [WLQ]) (63). Thus, comparing presenteeism effects across studies often remains challenging. For practice, this highlights the need for standardized measurement tools and reporting guidelines to enhance comparability across studies.

### Barriers to effective OHI

Dissemination and implementation science emphasizes that the success of OHI depends not only on the intervention itself but also on how it is implemented and communicated within an organizational context. Thereby, implementation refers to the process of integrating OHI into the organization, while dissemination focuses on how OHI information is actively communicated (107).

Several authors highlighted that implementation success depends heavily on leadership engagement and communication. For example, Stansfeld et al (80) found that limited senior management support hindered the effectiveness of a mental-health e-learning intervention, while Larsen et al (62) identified weak internal communication as a barrier to improving nurses’ health literacy. In contrast, Hendriksen et al (46) showed that strong managerial involvement and an enabling culture were linked to reduced sickness absence.

Furthermore, OHI implementations also tend to fail when they do not address the specific health risks or working conditions of the target population (108). For example, Brox & Frøystein (33) found no reduction in sick days following an aerobic fitness intervention in the field of elderly care. However, the intervention failed to address the population’s primary issues, such as neck, shoulder, and back pain, which were likely related to poor posture rather than low activity levels. Additional barriers, such as shift work-hindered attendance, were not adequately addressed and are often overlooked in similar interventions. In contrast, the highest-quality study in our sample with a significant reduction in sickness absence, van de Poll et al (57) reported an impressive mean difference of -15 days. This success can, amongst others, be attributed to the intervention’s individualized approach, which included interviews with both managers and employees, joint problem-solving sessions, and follow-ups to address specific work situations (70, 108).

Lastly, organizations unfamiliar with OHI may struggle with implementation. As Nielsen et al (108) noted cultural fit and change management capacity are key to translating OHI potential into measurable outcomes. As such, organizations with limited prior experience with OHI may encounter greater challenges in adopting such interventions.

### Targeting and implementing OHI

Next, we present two frameworks helping decision-makers to decide which OHI to select and how to implement them.

The US the National Institute for Occupational Safety and Health's total worker health (TWH) framework stresses that improving employee well-being starts with changing the cultural, social, and physical environment before targeting individual behavior (109). The framework outlines a five-step hierarchy for targeting OHI strategies, including (i) stressor elimination, (ii) substitution with safer alternatives, (iii) work re-design, (iv) education and training, and (v) behavioral change (110).

Several studies in our sample modified organizational or physical conditions to eliminate stressors. For instance, Åkerström et al (26) implemented an organizational-level job stress program mandating structural work changes (eg, work schedule improvements), while Framke et al (39) introduced participatory organization-level intervention to reduce unnecessary work tasks. On the contrary, many interventions in our sample focused on individual behavior without broader organizational redesign (eg, fitness programs or self-care workshops). From a practical perspective, organizations should assess where they currently stand within the TWH hierarchy and prioritize interventions that address their most pressing deficits, beginning with higher-level structural or organizational changes when these have not yet been established.

While the TWH framework supports the decision on which hierarchy levels to address, the WHO's healthy workplace model offers a holistic eight-step framework (ie, mobilize, assemble, assess, prioritize, plan, do, evaluate, improve) for translating such strategies into practice, keeping leadership engagement and worker participation at the core (111). Applying this model may help overcome several aforementioned implementation barriers. For instance, the mobilize step emphasizes securing top-management engagement and anchoring health as a strategic organizational priority, while the assessment stage promotes systematic evaluation of working conditions through employee input (eg, surveys) to ensure interventions address real needs. The subsequent evaluate–improve cycle supports structured implementation and iterative learning, enabling organizations unfamiliar with OHI to build experience and adapt interventions over time. For practice, the model may offer a pragmatic roadmap to secure successful OHI implementation and enhance effectiveness.

### Limitations and research outlook

Methodologically, grey literature was not systematically screened, which may introduce publication bias. Yet, focusing on published peer-reviewed studies ensures a minimum methodological quality and aligns with previous systematic searches (12, 96, 112). Additionally, we focused mainly on sickness absence and ROI; future research could broaden the scope to include presenteeism outcomes measured by validated tools such as the WLQ or the Work Productivity and Activity Impairment (WPAI) scale (113, 114). Additionally future reviews could incorporate physiological and wellbeing-related measures to better understand the full spectrum of OHI effects and contributors.

Second, RCT in workplace health settings are inherently difficult to implement. Strong organizational commitment, often a prerequisite, may bias results positively. Outcomes are shaped by complex real-world factors (eg, leadership behavior and organizational climate), which may not be fully controlled (115). We used the JBI assessment to focus on RCT with high methodological quality.

Moreover, numerous studies evaluated multi-component interventions, making it often challenging to completely isolate the effects by intervention type (eg 33, 61, 64, 68, 75,). Such interventions typically include multiple concurrent strategies (eg, physical exercise combined with mental health counselling and ergonomic adjustments), meaning observed outcomes might result from interactions among these components rather than any single element alone. Excluding these multi-component interventions would have significantly reduced the sample size. Van Dongen et al (87) is one of the few studies to report disaggregated outcomes by intervention type, and a leading example that allows investigators to separate effects by OHI type. To further disentangle mixed-effects of multi-component OHI, future research can use factorial designs or component analyses to disentangle intervention mechanisms clearly and identify the most effective components.

Most of the included studies had follow-up periods of 12–24 months, thus, potential long-term effects of OHI on absenteeism, well-being, and workplace conditions may therefore not be fully captured. Future studies could attempt to extend follow-up periods to capture the effects after two years and beyond.

Older studies mostly evaluated in-person OHI (eg, in-person fitness sessions), thus limiting generalizability to today’s digital or hybrid interventions (eg, app-based exercise programs), where factors like engagement and adherence differ. Digital OHI offer rapid scalability, easier deployment, and reduced set-up costs, potentially increasing ROI (116). These characteristics make them particularly attractive for small and medium-sized enterprises and dispersed or remote workforces. However, factors such as reduced engagement may impact their effectiveness (117). As the landscape of OHI evolves, ongoing evaluations are warranted to assess the comparative value of digital versus in-persona.

This meta-analysis was limited to 23 studies due to missing CI or incompatible outcome formats. Many excluded studies reported directional improvements, suggesting that the true effect size may be underestimated. Adherence to established reporting guidelines [eg, EQUATOR network (118)] could improve future data validity.

Lastly, our review was restricted to studies conducted in OECD countries, limiting generalizability to low- and middle-income contexts, where OHI effectiveness may differ given availability of (financial) resources, differing labor regulations, or distinct cultural attitudes toward OHI and absenteeism. Additionally, our study has shown overrepresentation of European studies (~70%), highlighting a need for increased research in underrepresented regions, such as North America (eg, Canada) and the Asia-Pacific region (eg, Japan).

### Concluding remarks

While our meta-analysis reveals no statistically significant effect on absenteeism, there is suggestive evidence that they may offer economic value, particularly in mental health interventions. Yet, the absence of short-term effects on sick days does not necessarily indicate a lack of effectiveness, given benefits such as individual well-being, employee engagement, or team dynamic may not be fully captured by quantitative absenteeism measures. Practically, this review guides decision-makers in selecting the most effective OHI from both absenteeism and economic perspectives and highlights key principles for implementation. Our findings highlight the likely economic value of workplace health promotion. Theoretically, this review enables cross-domain comparison of OHI, sheds light on study quality and heterogeneity of absenteeism and economic related studies, and underscores the need for more standardized evaluation approaches and reporting standards. Future research should measure presenteeism directly, expand beyond OECD contexts, and assess qualitative success factors such as leadership and cultural fit in sustaining OHI impact.

## Supplementary material

Supplementary material

## Data Availability

The code and data will be uploaded to GitHub upon project completion, OR the code and data will be available from the corresponding author upon request.
