# Supplementary material for: Occupational health interventions’ impact on absenteeism and economic returns: A systematic review and meta-analysis
Source: Scand J Work Environ Health. 2026 Feb 28;52(2):79–97. doi: 10.5271/sjweh.4265 (PMC12954742; doi:10.5271/sjweh.4265)

Occupational health interventions' impact on absenteeism and economic returns: A systematic review and meta-analysis[1]

*By Jonas Backes, MSc[2] Sonja I. Mueller, MSc, Alexander Geissler, PhD, David Ehlig, PhD*

1. Supplementary material
2. Correspondence to: Jonas Backes, University of St.Gallen School of Medicine, Jakob-Strasse 21, 9000 St.Gallen Switzerland.[E-mail: Jonas.backes@unisg.ch]

**Contents**

Illustration 1. Search strategy .......... 1

Table 1. Inclusion and exclusion criteria .......... 2

Table 2. Risk of bias assessment .......... 4

Table 3. List of all reviews with inclusion/exclusion decision .......... 8

Table 4. Vignettes for studies included in the systematic review .......... 10

Table 5. Summary statistics of papers included in the review (n = 68) .......... 79

Table 6. Overview of JBI critical appraisal results .......... 80

Figure 1. Search results by intervention type and outcome measure .......... 84

Figure 2. Evolution of OHI delivery formats for studies included in the systematic review .......... 86

Figure 3. Overview of study results and risk of bias for selected studies clustered by industry .......... 87

Figure 4. Forest plots of sub-group analyses .......... 89

**Illustration 1. Search strategy**

**PubMed Search:**

**1.** Keyword combinations for **work-related setting**

(Work*[ti] OR employ*[ti] OR occupational[ti] OR job[ti] OR labor[ti] OR OHS[ti] OR company*[ti])
AND (health[ti] OR well-being[ti] OR wellness[ti] OR prevent*[ti] OR assistan*[ti])
AND (intervention*[ti] OR program*[ti] OR strateg*[ti] OR initiative*[ti])

2. Keyword combinations for **mental health**

Mental[tiab] OR psych*[tiab] OR stress[tiab] OR depress*[tiab] OR therapy*[tiab] OR cognit*[tiab] OR cognitive behavioral therapy[tiab] OR CBT[tiab] OR mindful*[tiab] OR burnout[tiab] OR anxiety[tiab] OR emotion*[tiab]

**3.** Keyword combinations for **physical activity**

Activ*[tiab] OR exercise[tiab] OR training[tiab] OR activ*[tiab] OR movement[tiab] OR fitness[tiab] OR sport*[tiab] OR sedentar*[tiab] OR physical[tiab]

4. Keyword combinations for **nutrition and weight management**

(Nutritition*[tiab] OR diet*[tiab] OR eating*[tiab] OR food[tiab] OR nutrient*[tiab] OR vitamin*[tiab])
OR (weight management[tiab] OR weight control[tiab] OR weight loss[tiab] OR weight reduction[tiab] OR body weight[tiab] OR BMI[tiab] OR body mass index[tiab] OR obesity[tiab] OR overweight[tiab])

**5.** Keyword combinations for **working climate**

Climate[tiab] OR environment[tiab] OR social[tiab] OR culture[tiab] OR atmosphere[tiab] OR psychosocial[tiab] OR relation*[tiab] OR satisfact*[tiab]

**6.** Keyword combinations for economics

Econom*[tiab] OR cost-effectiv*[tiab] OR cost-benefit[tiab] OR ROI[tiab] OR return on investment[tiab] OR economic impact[tiab]

**7.** Keyword combinations for sickness absence

Claims[tiab] OR sick days[tiab] OR sickness days[tiab] OR sickness absence*[tiab] OR sick leave[tiab]

**Web of Science Search:**

**1.** Keyword combinations for **work-related setting**

Work* OR employ* OR occupational OR job OR labor OR OHS OR company*)
AND (health OR well-being OR wellness OR prevent* OR assistan*)
AND (intervention* OR program* OR strateg* OR initiative*

**2.** Keyword combinations for **mental health**

Mental OR psych* OR stress OR depress* OR therapy* OR cognit* OR "cognitive behavioral therapy" OR CBT OR mindful* OR burnout OR anxiety OR emotion*

**3.** Keyword combinations for **physical activity**

Activ* OR exercise OR training OR movement OR fitness OR sport* OR sedentar* OR physical

4. Keyword combinations for nutrition and weight management

(nutrition* OR diet* OR eating* OR food OR nutrient* OR vitamin*)
OR (weight management OR weight control OR weight loss OR weight reduction OR body weight OR BMI OR "body mass index" OR obesity OR overweight)

**5.** Keyword combinations for **working climate**

Climate OR environment OR social OR culture OR atmosphere OR psychosocial OR relation* OR satisfact*

**6.** Keyword combinations for economics

Econom* OR cost-effectiv* OR cost-benefit OR ROI OR "return on investment" OR "economic impact"

**7.** Keyword combinations for sickness absence

Claims OR "sick days" OR "sickness days" OR "sickness absence*" OR "sick leave"

**Illustration 2. Derivation of time-scaling for confidence intervals**

To compare absenteeism rates over different study durations, effects are scaled for a common period of 12 months (*T*). For sick days, we increased the expected mean linearly with time. Meanwhile its variance grows proportionally to the observation length.
Let *t* be the study's original follow-up and *T* the target period of 12-month. Let *μ* the mean difference in sick days between intervention and control group and *SE* its standard error.

*1. Mean scaling (linear time):*

$$\mu_T = \mu_t \times \frac{T}{t}$$

*2. Variance scaling (proportionally to time if outcomes are independent):*

$$VAR_T = VAR_t \times \frac{T}{t}$$

*3. Calculating the SE (based on the square root of VAR):*

$$SE_T = SE_t \times \sqrt{\frac{T}{t}}$$

*4. Calculating the 12-month CI (based on the SE and μ):*

$$CI_T = \mu_T \pm Z \times SE_t \times \sqrt{\frac{T}{t}}$$

**Table S1. Inclusion and exclusion criteria**

| Criteria | Inclusion | Exclusion |
|---|---|---|
| Study type | Completed, primary quantitative articles including but not limited to experimental, quasi-experimental, observational and modelling studies published in peer-reviewed journals | Uncompleted or qualitative heavy articles including but not limited to protocols, systematic reviews, meta-studies, opinion pieces or any study not published in peer-reviewed journals |
| Intervention type | Intervention focuses on improvement of mental health, physical health & fitness, nutrition, as well as working atmosphere | Focus on interventions that do not address the topics of mental health, physical health & fitness, nutrition, as well as working atmosphere |
| Intervention setting | Employer/work-related intervention | Non-employer/work-related intervention |
| Country scope | Developed countries classified by OECD (2024) | Other Countries |
| Outcome measures | Economic return and sick days clearly quantified with numeric outcomes respective statistics (e.g., CI, SE, etc.) | No quantification of outcomes variables with significant lack in statistical descriptives |
| Participants | >18 years of age | <18 years of age |
| Language | English or German | Not English or German |
| Time | 2004 onwards | 2004 and before |

**Table S2. Risk of bias assessment**

The risk of bias of the papers included was assessed using the Joanna Briggs Institute's (JBI) critical appraisal tools. The JBI tools provide a comprehensive and standardized approach to assessing the methodological quality of diverse study designs, ensuring reliability and consistency in the evaluation process.
The length of each checklist varied depending on the assessment type, with response options including "Yes," "No," "Unclear," and "N/A." A numerical score was calculated based on the proportion of "Yes" responses, expressed as a percentage of all questions answered. During the evaluation, it was identified that two specific questions from the RCT appraisal checklist (i.e., "Were those delivering the treatment blind to treatment assignment?" and "Were treatment groups treated identically other than the intervention of interest?") did not provide meaningful differentiation across reviews. This was attributed to the unrealistic expectation of strictly double-blinded and controlled designs in workplace settings (Barthelmes et al., 2019). Consequently, Questions 5 and 6, often unfulfilled, were removed from the RCT checklist. For similar considerations, Question 4 from the critical appraisal tool for quasi-experimental studies was excluded.
The exact criteria and the systematic method of evaluation employed to ensure fair and consistent assessment are described in the following.

JBI critical appraisal tool for assessment of risk of bias for randomized controlled trials (2023)

| JBI Question | Systematic evaluation method |
|---|---|
| 1. Was true randomization used for assignment of participants to treatment groups? | Always "Yes", as otherwise, it is not an RCT. |
| 2. Was allocation to treatment groups concealed? | "Yes" if the randomization was performed by an independent researcher, or a computer algorithm was used, or a coin was flipped. |
| 3. Were treatment groups similar at the baseline? | "Yes" if there were only minimal differences or if these were controlled for in the analysis. |
| 4. Were participants blind to treatment assignment? | "Yes" or '"No" if explicitly stated, otherwise "Unclear". |
| 5. Were those delivering the treatment blind to treatment assignment? | Excluded due to feasibility considerations for this type of interventions. |
| 6. Were treatment groups treated identically other than the intervention of interest? | Excluded due to feasibility considerations for this type of interventions. |
| 7. Were outcome assessors blind to treatment assignment? | "Yes" or '"No" if explicitly stated, otherwise "Unclear". |
| 8. Were outcomes measured in the same way for treatment groups? | Always "Yes", as no study indicated any evidence of different measurements. |
| 9. Were outcomes measured in a reliable way? | "Yes" if Cronbach's alpha was specified or standardized, validated and commonly used questionnaires or non-self-reported data from insurers or employees were used for at least one outcome variable. "No" if only undefined questionnaires or questionnaires modified without details were used. |
| 10. Was follow up complete and if not, were differences between groups in terms of their follow up adequately described and analysed? | "Yes" if follow-up was complete. If not complete: "No" if the authors do not describe differences in characteristics between completers and non-completers. "No" if differences were found but not analyzed. |

| JBI Question | Systematic evaluation method |
|---|---|
| | Otherwise “Yes” or “Unclear” if no information was provided. |
| 11. Were participants analysed in the groups to which they were randomized? | “Yes” or '“No” if explicitly stated, otherwise “Unclear”. |
| 12. Was appropriate statistical analysis used? | “No” if no power analyses were performed, or any confounders were included in the analysis, or the methods were not appropriate, or important data were not reported. Otherwise “Yes”. |
| 13. Was the trial design appropriate and any deviations from the standard RCT design accounted for in the conduct and analysis of the trial? | “Yes” for all studies except one, which is “Unclear” because it discusses the design itself and indicates it as a potential limitation. |

JBI critical appraisal tool for assessment of risk of bias for economic evaluations (2020)

| JBI Question | Systematic evaluation method |
|---|---|
| 1. Is there a well-defined question? | Always “Yes”. |
| 2. Is there a comprehensive description of alternatives? | “Yes” or “No”. |
| 3. Are all important and relevant costs and outcomes for each alternative identified? | Always “Yes” if reasonably comprehensive costs and outcomes were identified, with one exception that excluded any intervention costs. |
| 4. Has clinical effectiveness been established? | “Yes” if the study was combined with an RCT or quasi-experiment with significant results or if relevant literature was reported. Otherwise “No”. |
| 5. Are costs and outcomes measured accurately? | “Yes” for insurer or company data. “No” if a large proportion is based on estimates or self-reported data, or data may have been collected inconsistently. |
| 6. Are costs and outcomes valued credibly? | “Yes” if both costs and outcomes were valued. “No” if insufficient justifications were given and insufficient differentiation was made. |
| 7. Are costs and outcomes adjusted for differential timing? | “Yes” for all studies that have not exceeded a time period of one year, because the discount rate in these cases is automatically 0. Otherwise “Yes” if explicitly specified, else “No”. |
| 8. Is there an incremental analysis of costs and consequences? | “Yes” or “No”. |
| 9. Were sensitivity analyses conducted to investigate uncertainty in estimates of cost or consequences? | “Yes” or “No”. |
| 10. Do study results include all issues of concern to users? | “Yes” as long as a comprehensive analysis and discussion has taken place and various aspects have been highlighted. “No” for very unidimensional analyses or the lack of important information for evaluating the results (i.e. details on statistical methods). |

| JBI Question | Systematic evaluation method |
|---|---|
| 11. Are the results generalizable to the setting of interest in the review? | “No” for studies on a single company or if generalizability was explicitly discussed. Otherwise “Yes”. |

JBI critical appraisal tool for assessment of risk of bias for quasi-experimental studies (2024)

| JBI Question | Systematic evaluation method |
|---|---|
| 1. Is it clear in the study what is the “cause” and what is the “effect” (ie, there is no confusion about which variable comes first)? | Always “Yes”. |
| 2. Was there a control group? | “Yes” or “No”. |
| 3. Were participants included in any comparisons similar? | “N/A” for missing control group. “Unclear” if details are absent. “No” if there were significant differences, otherwise “Yes”. |
| 4. Were the participants included in any comparisons receiving similar treatment/care, other than the exposure or intervention of interest? | Excluded due to feasibility considerations for this type of interventions. |
| 5. Were there multiple measurements of the outcome, both pre and post the intervention/exposure? | Always “Yes”. |
| 6. Were the outcomes of participants included in any comparisons measured in the same way? | “N/A” for missing control group. “Unclear” if details are absent. "No" if there were differences in measurement, otherwise "Yes". |
| 7. Were outcomes measured in a reliable way? | “Yes” if Cronbach's alpha was specified or standardized, validated and commonly used questionnaires or non-self-reported data from insurers or employees were used for at least one outcome variable. “No” if details were absent ore only undefined questionnaires or questionnaires modified without details were used. |
| 8. Was follow up complete and if not, were differences between groups in terms of their follow up adequately described and analysed? | “N/A” for missing control group. “Yes” if follow-up was complete. If not complete: “No” if the authors do not describe differences in characteristics between completers and non-completers. “No” if differences were found but not analyzed. Otherwise “Yes” or “Unclear” if no information was provided. |
| 9. Was appropriate statistical analysis used? | “No” if no power analyses were performed, or any confounders were included in the analysis, or the methods were not appropriate, or important data were not reported. Otherwise “Yes”. |

JBI critical appraisal tool for assessment of risk of bias for cohort studies (2020)

| JBI Question | Systematic evaluation method |
|---|---|
| 1. Were the two groups similar and recruited from the same population? | "Yes" or "Unclear". |
| 2. Were the exposures measured similarly to assign people to both exposed and unexposed groups? | "Yes" or "Unclear". |
| 3. Was the exposure measured in a valid and reliable way? | "Yes" if standardized, validated and commonly used questionnaires or non-self-reported data from insurers or employees were used for at least one outcome variable. "No" if only undefined questionnaires or questionnaires modified without details were used. |
| 4. Were confounding factors identified? | Always "Yes". |
| 5. Were strategies to deal with confounding factors stated? | Always "Yes". |
| 6. Were the groups/participants free of the outcome at the start of the study (or at the moment of exposure)? | "Yes" or "Unclear". |
| 7. Was the exposure measured in a valid and reliable way? | "Yes" if standardized, validated and commonly used questionnaires or non-self-reported data from insurers or employees were used for at least one outcome variable. "No" if only undefined questionnaires or questionnaires modified without details were used. |
| 8. Was the follow up time reported and sufficient to be long enough for outcomes to occur? | Always "Yes". |
| 9. Was follow up complete, and if not, were the reasons to loss to follow up described and explored? | Always "No" as no reasons were given. |
| 10. Were strategies to address incomplete follow up utilized? | "Yes" or "No". |
| 11. Was appropriate statistical analysis used? | "No" if no power analyses were performed, or any confounders were included in the analysis, or the methods were not appropriate, or important data were not reported. Otherwise "Yes". |

**Table S3. List of all reviews with inclusion/exclusion decision**

| Ref | Authors | Year | Inclusion/exclusion | Reason for exclusion |
|---|---|---|---|---|
| (26) | Akerstrom et al. | 2021 | Inclusion | N/A |
| - | Aldana et al. | 1993 | Exclusion | >20 years old |
| (27) | Arends et al. | 2013 | Inclusion | N/A |
| - | Avey et al. | 2022 | Exclusion | Outcomes not applicable |
| (28) | Baker et al. | 2008 | Inclusion | N/A |
| - | Bergstroem et al. | 2007 | Exclusion | Intervention not identifiable |
| (29) | Blake et al. | 2013 | Inclusion | N/A |
| (30) | Blangsted et al. | 2008 | Inclusion | N/A |
| (31) | Bondar et al. | 2022 | Inclusion | N/A |
| (32) | Braun et al. | 2014 | Inclusion | N/A |
| (33) | Brox and Frøystein | 2005 | Inclusion | N/A |
| - | Burton et al. | 2014 | Exclusion | Intervention not identifiable |
| - | Chung et al. | 2009 | Exclusion | Outcomes not applicable |
| (34) | De Boer et al. | 2004 | Inclusion | N/A |
| - | Declercq et al. | 2022 | Exclusion | Protocol |
| (35) | Dement et al. | 2015 | Inclusion | N/A |
| (36) | Duijts et al. | 2008 | Inclusion | N/A |
| (37) | Ebert et al. | 2018 | Inclusion | N/A |
| (38) | Elson et al. | 2019 | Inclusion | N/A |
| - | Erfurt et al. | 1992 | Exclusion | >20 years old |
| - | Erfurt et al. | 1992 | Exclusion | Duplication |
| - | Eriksen et al. | 2002 | Exclusion | >20 years old |
| (39) | Framke et al. | 2016 | Inclusion | N/A |
| (40) | Freund et al. | 2024 | Inclusion | N/A |
| - | Fries et al. | 1993 | Exclusion | >20 years old |
| - | Frost et al. | 2007 | Exclusion | No access |
| (41) | Geraedts et al. | 2015 | Inclusion | N/A |
| (42) | Goetzel et al. | 2014 | Inclusion | N/A |
| (43) | Gregson et al. | 2023 | Inclusion | N/A |
| (44) | Groeneveld et al. | 2011 | Inclusion | N/A |
| (45) | Gubler et al. | 2017 | Inclusion | N/A |
| - | Helma et al. | 2007 | Exclusion | Intervention not in focus |
| (46) | Hendriksen et al. | 2016 | Inclusion | N/A |
| (47) | Hengel et al. | 2013 | Inclusion | Outcomes not applicable |
| (48) | Hengel et al. | 2014 | Inclusion | N/A |
| (49) | Herman et al. | 2008 | Inclusion | N/A |
| - | Hoopmann et al. | 2001 | Exclusion | >20 years old |
| (50) | Hughes et al. | 2007 | Inclusion | N/A |
| - | Hulten et al. | 2021 | Exclusion | Intervention not in focus |
| - | Hultqvist et al. | 2021 | Exclusion | Intervention not identifiable |
| (51) | Iijima et al. | 2013 | Inclusion | N/A |
| (52) | Ikegami et al. | 2010 | Inclusion | N/A |
| (53) | Jenny et al. | 2011 | Inclusion | N/A |
| - | Johnsen et al. | 2018 | Exclusion | Intervention as comparator |
| (54) | Jorgensen et al. | 2011 | Inclusion | N/A |
| (55) | Kapinos et al. | 2015 | Inclusion | N/A |
| (56) | Karlsson et al. | 2024 | Inclusion | N/A |

| Ref | Authors | Year | Inclusion/exclusion | Reason for exclusion |
|---|---|---|---|---|
| (57) | Keus van de Poll et al. | 2020 | Inclusion | N/A |
| (58) | Keus van de Poll et al. | 2020 | Inclusion | N/A |
| - | Kinchin et al. | 2017 | Exclusion | Not employer perspective |
| - | Klarreich et al. | 1987 | Exclusion | >20 years old |
| (59) | Klasen et al. (RCT 2) | 2021 | Inclusion | N/A |
| (60) | Kobayashi et al. | 2008 | Inclusion | N/A |
| (61) | Krampen | 2010 | Inclusion | N/A |
| - | Kuorinka et al. | 1994 | Exclusion | >20 years old |
| - | Lahiri et al. | 2005 | Exclusion | Intervention not in focus |
| (62) | Larsen et al. | 2019 | Inclusion | N/A |
| (63) | Lerner et al. | 2020 | Inclusion | N/A |
| (64) | Linden et al. | 2014 | Inclusion | N/A |
| - | Loeppke et al. | 2008 | Exclusion | >20 years old |
| (65) | Losina et al. | 2017 | Inclusion | N/A |
| (66) | Makrides et al. | 2011 | Inclusion | N/A |
| - | Maniscalco et al. | 1999 | Exclusion | >20 years old |
| (67) | Meenan et al. | 2010 | Inclusion | N/A |
| (68) | Milani et al. | 2009 | Inclusion | N/A |
| - | Mohandas et al. | 2022 | Exclusion | No access |
| (69) | Musich et al. | 2015 | Inclusion | N/A |
| - | Nadolski et al. | 1987 | Exclusion | >20 years old |
| - | Noben et al. | 2014 | Exclusion | Duplication |
| (70) | Noben et al. | 2014 | Inclusion | N/A |
| (71) | Norwitz et al. | 2022 | Inclusion | N/A |
| (72) | Ornek et al. | 2020 | Inclusion | N/A |
| - | Pegus et al. | 2002 | Exclusion | >20 years old |
| (73) | Proper et al. | 2004 | Inclusion | N/A |
| - | Rantonen et al. | 2011 | Exclusion | Duplication |
| (74) | Rantonen et al. | 2018 | Inclusion | N/A |
| (75) | Renaud et al. | 2008 | Inclusion | N/A |
| (76) | Robroek et al. | 2012 | Inclusion | N/A |
| - | Rollison et al. | 2022 | Exclusion | Intervention not identifiable |
| (77) | Ryan et al. | 2018 | Inclusion | N/A |
| (78) | Saleh et al. | 2010 | Inclusion | N/A |
| - | Shaw et al. | 2014 | Exclusion | Protocol |
| - | Shimizu et al. | 2003 | Exclusion | >20 years old |
| - | Song et al. | 2019 | Exclusion | Duplication |
| (79) | Song et al. | 2021 | Inclusion | Follow-up study |
| (80) | Stansfeld et al. | 2015 | Inclusion | N/A |
| - | Stein et al. | 2000 | Exclusion | >20 years old |
| - | Syrjälä et al. | 2022 | Exclusion | Protocol |
| (81) | Taimela et al. | 2009 | Inclusion | N/A |
| - | Taimela et al. | 2008 | Exclusion | Duplication |
| (82) | Taimela et al. | 2007 | Inclusion | N/A |
| (83) | Thiart et al. | 2016 | Inclusion | N/A |
| (84) | Tveito and Eriksen | 2008 | Inclusion | N/A |
| (85) | Van den Ven et al. | 2023 | Inclusion | N/A |
| (86) | Van Dongen et al. | 2013 | Inclusion | N/A |
| (87) | Van Dongen et al. | 2017 | Inclusion | N/A |

| Ref | Authors | Year | Inclusion/exclusion | Reason for exclusion |
|---|---|---|---|---|
| (88) | Van Holland et al. | 2017 | Inclusion | N/A |
| (89) | Viester et al. | 2014 | Inclusion | N/A |
| (90) | Von Thiele Schwarz et al. | 2012 | Inclusion | N/A |
| - | Von Thiele Schwarz et al. | 2011 | Exclusion | Duplication |
| (91) | Vonderlin et al. | 2023 | Inclusion | N/A |
| - | Wickizer et al. | 2011 | Exclusion | Intervention not in focus |
| (92) | Wijnen et al. | 2019 | Inclusion | N/A |
| - | Yoshimura et al. | 2013 | Exclusion | Not in English or German |
| (93) | Zetterberg et al. | 2022 | Inclusion | N/A |

**Table S4. Vignettes for studies included in the systematic review**

**(26) Akerstrom et al. (2021)**

| | | | |
|---|---|---|---|
| **Title** | Can Working Conditions and Employees' Mental Health Be Improved via Job Stress Interventions Designed and Implemented by Line Managers and Human Resources on an Operational Level? | | |
| **Country scope** | Sweden | | |
| **Industry scope** | Mainly health care | | |
| **Study type** | Quasi-Experiment | | |
| **JBI evaluation** | Risk of bias: Medium | | |
| **PICO framework** | | | |
| **Population** | Public sector employees, with approximately 85% working in the healthcare sector. | | |
| | Sample size: N/A | Mean age: N/A | Female ratio: N/A |
| **Intervention(s)** | Domain: Working atmosphere | | |
| | Type: Work climate change | | |
| | A workplace-wide stress intervention was designed and implemented by line managers in collaboration with HR partners at the operational level. It focused on modifying work processes and how tasks were performed. | | |
| | Duration: ~24 months | | |
| | Setting: Phone-based, in-person | | |
| **Comparator** | Reference data from 247 workplace groups across 18 operational areas and 10 departments, matching the organizational level of the intervention groups. Employee turnover and sick leave data were only available in aggregated form. | | |

| | |
|---|---|
| **Outcome** | Outcome measure(s): Sickness absence rate<br><br>Registry data on sickness absence rate as well as data on working conditions, motivation, health, employee turnover |
| **Results** | The intervention had no significant effect on overall sickness rates but led to a statistically significant reduction in short-term illnesses (≤14 days) by 0.2 percentage points in the intervention group. |

**(27) Arends et al. (2013)**

| | |
|---|---|
| **Title** | Economic Evaluation of a Problem Solving Intervention to Prevent Recurrent Sickness Absence in Workers with Common Mental Disorders |
| **Country scope** | Netherlands |
| **Industry scope** | Not specified |
| **Study type** | RCT |
| **JBI evaluation** | Risk of bias: Low |
| **PICO framework** | |
| **Population** | Employees aged 18 to 63 years with a common mental disorder (CMD) diagnosis at the start of sickness absence and the capacity for partial or full return to work.<br>Sample size: 158 \| Mean age: 42.3 \| Female ratio: 59% |
| **Intervention(s)** | Domain: Working atmosphere<br>Type: Education and training opportunities<br>The intervention group received the SHARP-at-work program, delivered by occupational physicians (OPs) when employees with a CMD diagnosis were ready to return to work. The program followed five steps: problem identification, solution brainstorming, documentation, action planning, and evaluation. OPs underwent a two-day training and were advised to conduct 2 to 5 consultations, each lasting approximately 30 minutes.<br>Duration: Not specified<br>Setting: In-person |
| **Comparator** | The control group followed the Netherlands Society of Occupational Medicine guidelines for managing workers with mental health issues. Occupational physicians conducted a single consultation after returning to work to prevent relapse. |
| **Outcome** | Outcome measure(s): Incidence/time to recurrent sickness absence<br>Primary: Incidence of recurrent sickness absence<br>Secondary: Time to recurrent sickness absence, cost-effectiveness ratio (CER), and cost-benefit analysis (CBA) |
| **Results** | Time to recurrent sickness absence was significantly shorter in the intervention group (253 vs. 365 days in CAU), with a mean effect difference of 55 days. However, no economic benefit was observed, as the intervention incurred €800 higher costs on average, making it more effective in reducing sickness absence but also more expensive. |

| (28) Baker et al. (2008) | | | |
|---|---|---|---|
| **Title** | Using a Return-On-Investment Estimation Model to Evaluate Outcomes from an Obesity Management Worksite Health Promotion Program | | |
| **Country scope** | United States | | |
| **Industry scope** | N/A | | |
| **Study type** | Quasi-Experimental | | |
| **JBI evaluation** | Risk of bias: High | | |
| **PICO framework** | | | |
| **Population** | Employees with a BMI of 30 or higher, or employees with a BMI of 25 or higher and a comorbid condition such as type 2 diabetes, high blood pressure, other cardiovascular diseases or other weight-related conditions. | | |
| | Sample size: 890 | Mean age: 44.2 | Female ratio: 74.3% |
| **Intervention(s)** | Domain: Physical health and fitness | | |
| | Type: Exercise programs, nutrition and weight management, health monitoring programs | | |
| | The Healthyroads program supports weight loss and health improvement through up to 48 telephone counseling sessions with a personal health coach, access to written materials and a health website, exercise planning, nutrition education, and a web-based health tracker. | | |
| | Duration: 12 months | | |
| | Setting: Phone-based, digital (web/app-based) | | |
| **Comparator** | N/A | | |
| **Outcome** | Outcome measure(s): ROI | | |
| | Self-reporting-based financial outcomes (including ROI) and prevalence of modifiable risk factors (e.g., insufficient physical activity) | | |
| **Results** | After one year, the program resulted in an estimated savings of $1.17 for every dollar invested. The total savings for employers amounted to USD 311,755, with 59% ($184,582) coming from reduced healthcare expenditures and 41% ($127,173) from productivity improvements. | | |

**(29) Blake et al. (2013)**

| | |
|---|---|
| **Title** | Five-year workplace wellness intervention in the NHS |
| **Country scope** | United Kingdom |
| **Industry scope** | Health care |
| **Study type** | Quasi-Experiment |
| **JBI evaluation** | Risk of bias: High |
| **PICO framework** | |
| **Population** | National Health Service (NHS) staff in a large acute hospital.<br>Sample size: 1,452 \| Mean age: 41.06 \| Female ratio: 79.6% |
| **Intervention(s)** | Domain: Physical health and fitness<br>Type: Exercise programs, nutrition and weight management, health monitoring programs<br>The five-year NHS workplace wellness program aimed to improve staff health and wellbeing, supported by dedicated funding and a health commissioner. It included health campaigns, fitness facilities, physical and dietary activities, relaxation techniques, health education, community engagement, cycling initiatives, workplace health advocates, and an online platform with activity schedules.<br>Duration: 6 months<br>Setting: In-person, digital (web/app-based) |
| **Comparator** | N/A |
| **Outcome** | Outcome measure(s): Sickness absence rate<br>Self-reported data on sick days, health and behavioral outcomes, psychosocial factors, work-related outcomes |
| **Results** | After the intervention, long-term sickness absence was lower in the intervention group at 6 months but slightly higher at 12 months. However, the differences were not statistically significant. After 12 months, the intervention group had an average of 8.5 sick days (SD: 20.6) compared to 7.5 days in the control group (SD: 16.9). Although the baseline sick days were lower in the control group, the intervention group showed a higher reduction in sick days. |

**(39) Blangsted et al. (2008)**

| | | | |
|---|---|---|---|
| **Title** | One-year randomized controlled trial with different physical-activity programs to reduce musculoskeletal symptoms in the neck and shoulders among office workers | | |
| **Country scope** | Denmark | | |
| **Industry scope** | Not assignable | | |
| **Study type** | RCT | | |
| **JBI evaluation** | Risk of bias: Medium | | |
| **PICO framework** | | | |
| **Population** | Office workers from a Danish national authority in the east of Denmark. Participants all worked in an office environment. | | |
| | Sample size: 616 | Mean age: 44.99 | Female ratio: 64.5% |
| Intervention(s) | Domain: Physical health and fitness | | |
| | Type: Exercise programs | | |
| | Two interventions were implemented: (1) Specific Resistance Training with three 20-minute weekly sessions to strengthen shoulder and neck muscles. (2) All-round Physical Exercise aimed at increasing overall activity, with motivational sessions, personalized contracts, and 1-4 trainer visits per month. | | |
| | Duration: 12 months | | |
| | Setting: In-person | | |
| **Comparator** | No additional physical activity; focused on improving workplace health (e.g., ergonomics, stress, indoor air) through group-led initiatives | | |
| **Outcome** | Outcome measure(s): Sick days, Sickness absence rate | | |
| | Total number of sick days in the last year: 3 months accumulated number of all sick days multiplied by 4 to obtain an annual value<br>Sickness absence rate: Changes in the sickness absence values of the WAI (work ability index) | | |
| **Results** | No statistically significant changes in sickness absence due to the interventions. At the beginning of the study, the average sickness absence of the participants in the last 3 months was 1.5 days, and the average sickness absence in the previous year was 5.3 days. | | |

| **(31) Bondar et al. (2022)** | | | |
|---|---|---|---|
| **Title** | Clinical and Financial Outcomes Associated With a Workplace Mental Health Program Before and During the COVID-19 Pandemic | | |
| **Country scope** | United States | | |
| **Industry scope** | Not assignable | | |
| **Study type** | Quasi-Experiment | | |
| **JBI evaluation** | Risk of bias: Medium | | |
| **PICO framework** | | | |
| **Population** | Employees with at least moderate anxiety or depression, determined by baseline scores on the PHQ-9 (≥10) or GAD-7 (≥10), who participated in a workplace mental health program. | | |
| | Sample size: 1,132 | Mean age: 32.9 | Female ratio: 71.8% |
| **Intervention(s)** | Domain: Mental health and stress | | |
| | Type: Mental health initiatives/training | | |
| | The digital mental health program offered included screening for common mental disorders, access to digital self-help content, guidance in finding appropriate care, as well as options for video and in-person psychotherapy or medication management. | | |
| | Duration: 6 months | | |
| | Setting: In-person, digital (web/app-based) | | |
| **Comparator** | N/A | | |
| **Outcome** | Outcome measure(s): ROI | | |
| | Primary: Self-reporting-based ROI, symptoms of depression and anxiety<br>Secondary: Increase in working hours and employee retention | | |
| **Results** | The study showed a favorable ROI across all wage groups. Employees with depression or anxiety saved $3,440 in salary costs over six months. Treatment cost $740 on average. Participants took 0.32 fewer sick days per week and reported 0.64 fewer unproductive days, indicating increased productivity. | | |

**(32) Braun et al. (2014)**

| | | | |
|---|---|---|---|
| **Title** | Better health at work? An evaluation of the effects and cost–benefits of a structured workplace health improvement program in reducing sickness absence | | |
| **Country scope** | United Kingdom | | |
| **Industry scope** | Not assignable | | |
| **Study type** | Quasi-Experiment | | |
| **JBI evaluation** | Risk of bias: High | | |
| **PICO framework** | | | |
| **Population** | Employees in Northeast England took part in the Better Health at Work Award (BHWA) program, involving 232 organizations and 21.4% of the regional workforce. Private sector participation was 49%. | | |
| | Sample size: N/A | Mean age: N/A | Female ratio: N/A |
| Intervention(s) | Domain: Working atmosphere, mental health & stress, physical health & fitness | | |
| | Type: Exercise programs, nutrition and weight management, health monitoring programs, mindfulness and relaxation, work climate change, mental health policies | | |
| | The "Better Health at Work Award" program has three levels—Bronze, Silver, and Gold—focusing on workplace improvements and health promotion, including physical activity, nutrition, health monitoring, and mental well-being. | | |
| | Duration: N/A | | |
| | Setting: N/A | | |
| **Comparator** | N/A | | |
| **Outcome** | Outcome measure(s): Sick days, cost-benefit (CBA) | | |
| | Sick days were measured as the reduction in sickness-related absences per employee per year | | |
| **Results** | Participation in the BHWA program reduced absenteeism by 0.26 to 1.6 days per employee per year, depending on the level achieved. Program costs ranged from £359 (Bronze) to £3,606 (Gold), with the cost per absence day saved estimated at £0.90 (Bronze) to £125 (Gold). | | |

| (33) Brox and Frøystein (2005) | | | |
|---|---|---|---|
| **Title** | Health-related quality of life and sickness absence in community nursing home employees: randomized controlled trial of physical exercise | | |
| **Country scope** | Norway | | |
| **Industry scope** | Health care | | |
| **Study type** | RCT | | |
| **JBI evaluation** | Risk of bias: Medium | | |
| **PICO framework** | | | |
| **Population** | Care home staff in a community-based facility for older people. | | |
| | Sample size: 129 | Mean age: 42.5 | Female ratio: 96.5% |
| Intervention(s) | Domain: Mental health & stress, physical health & fitness | | |
| | Type: Exercise programs, nutrition and weight management, mindfulness & relaxation | | |
| | Weekly one-hour light group exercise based on an aerobic fitness model designed to improve cardiovascular fitness, muscular strength and flexibility. In addition, the intervention group was offered courses on exercise, nutrition and stress management. | | |
| | Duration: 6 months | | |
| | Setting: In-person | | |
| **Comparator** | Control group was not offered any intervention | | |
| **Outcome** | Outcome measure(s): Sick days | | |
| | Sick days recorded over two 7-month periods, starting on 1 April of the year before and the year of the intervention | | |
| **Results** | Sickness absence rose in both groups (from 6.8 to 15.6 in the intervention and from 10.4 to 14.5 in the control group), with a statistically significant increase in the intervention group, resulting in a 4.7-day difference (95% CI: -5.7 to 15.0) favoring the control group. No changes were observed in self-certified sick days, but both groups experienced increases in the number of long-term sick leave cases and the duration of these absences. | | |

**(34) De Boer et al. (2004)**

| | |
|---|---|
| **Title** | An Occupational Health Intervention Programme for Workers at Risk for Early Retirement; A Randomised Controlled Trial |
| **Country scope** | Netherlands |
| **Industry scope** | Technology |
| **Study type** | RCT |
| **JBI evaluation** | Risk of bias: Low |
| **PICO framework** | |
| **Population** | Employees over the age of 50 reporting they would not be able to work until the normal retirement age.<br>Sample size: 116 / Mean age: 53.4 / Female ratio: 7% |
| **Intervention(s)** | Domain: Physical health and fitness<br>Type: Health monitoring programs<br>The occupational health program, conducted by the respective company doctors of the participating employees, included at least three counseling sessions, the creation of a detailed action plan, consultations with superiors and HR managers, and, if necessary, referrals to GPs, specialists, or psychologists.<br>Duration: 6 months<br>Setting: In-person |
| **Comparator** | Care as usual, meaning participants were not invited to a counseling interview but could visit the company doctor at any time. |
| **Outcome** | Outcome measure(s): Sick days<br>Primary: Early retirement<br>Secondary: Registry data on sick days (6, 12 and 24 months), ability to work, stress-related symptoms, quality of life, and satisfaction with the intervention |
| **Results** | Differences in sick leave days were not statistically significant, with the intervention group averaging 15.4 days compared to 21.4 days in the control group during the first six months ($p = 0.23$). Over two years post-randomization, the intervention group averaged 82.3 sick leave days, while the control group averaged 107.8 days. |

| (35) Dement et al. (2015) | | | |
|---|---|---|---|
| **Title** | Impacts of Workplace Health Promotion and Wellness Programs on Health Care Utilization and Costs Results From an Academic Workplace | | |
| **Country scope** | United States | | |
| **Industry scope** | Health care | | |
| **Study type** | Cohort study | | |
| **JBI evaluation** | Risk of bias: Low | | |
| **PICO framework** | | | |
| **Population** | Employees from a large university hospital system, including an academic medical center and two regional hospitals, with roles in research, teaching, administration, and patient care. | | |
| | Sample size: 10,432 | Mean age: 41.55 | Female ratio: 58% |
| **Intervention(s)** | Domain: Physical health and fitness | | |
| | Type: Exercise programs, nutrition and weight management, health monitoring programs | | |
| | The comprehensive workplace health promotion program, offered free to eligible employees for over 20 years, aimed to improve health indicators like smoking, blood pressure, cholesterol, weight, physical activity, and stress. Key elements included health risk assessments, voluntary screenings, health coaching, weight management classes, fitness counseling, running/walking clubs, activity campaigns, and access to farmers' markets and fitness clubs, all managed by an external provider. | | |
| | Duration: N/A | | |
| | Setting: In-person, digital (web/app-based) | | |
| **Comparator** | Employees who did not complete a health risk assessment (HRA) during the study period from 2006 to 2011 and did not participate in the disease management program in the two years before or during the study period. | | |
| **Outcome** | Outcome measure(s): ROI | | |
| | Self-reported and registry data on ROI (savings divided by cost of the HP program [including intervention, personnell and administrative costs]), service utilization | | |
| **Results** | The study estimated a return on investment (ROI) of 2.53 for every dollar invested in the health promotion (HP) program, based on an average monthly saving of $35 in healthcare costs per participant. | | |

**(36) Duijts et al. (2008)**

| | | | |
|---|---|---|---|
| **Title** | Effectiveness of a Preventive Coaching Intervention for Employees at Risk for Sickness Absence Due to Psychosocial Health Complaints: Results of a Randomized Controlled Trial | | |
| **Country scope** | Netherlands | | |
| **Industry scope** | Health care, consumer services | | |
| **Study type** | RCT | | |
| **JBI evaluation** | Risk of bias: Medium | | |
| **PICO framework** | | | |
| **Population** | Employees at high risk of sickness absence due to psychosocial health issues, excluding those on sick leave. | | |
| | Sample size: 151 | Mean age: 42.8 | Female ratio: 82% |
| **Intervention(s)** | Domain: Working atmosphere | | |
| | Type: Education & training opportunities | | |
| | The intervention group received a preventive coaching program with 7 to 9 one-hour sessions, led by eight coaches from the 'Capability' organization. The program included goal-setting, a planning session with the supervisor, individual coaching on behavioral change, and a final evaluation. | | |
| | Duration: 4.5 months | | |
| | Setting: In-person | | |
| **Comparator** | The control group received the usual care in their companies, such as counselling from social workers or company doctors, if required. | | |
| **Outcome** | Outcome measure(s): Sick days | | |
| | Primary: Self-reported and registry data on sick days due to mental health status (never, 1, 2, 3, 4, 5, 5+; 12 months)<br>Secondary: General health, mental stress, anxiety, coping strategies, mental work characteristics, recovery needs, fatigue, burnout, and various socio-demographic and organizational characteristics | | |
| **Results** | Employees in the coaching group had 2.5 fewer sick days (14% reduction) in the year after baseline compared to usual care, with a mean difference of −2.53 days in favor of the intervention group. Secondary outcomes showed improvements in self-reported health, psychological distress, burnout, and anxious mood in the intervention group. | | |

| (37) Ebert et al. (2018) | | | |
|---|---|---|---|
| **Title** | A health economic outcome evaluation of an internet-based mobile-supported stress management intervention for employees | | |
| **Country scope** | Germany | | |
| **Industry scope** | N/A | | |
| **Study type** | RCT | | |
| **JBI evaluation** | Risk of bias: Low | | |
| **PICO framework** | | | |
| **Population** | Employees experiencing increased stress symptoms, with participants scoring ≥22 on the Perceived Stress Scale (PSS-10). Recruitment was primarily conducted through the occupational health program of a large health insurance company in Germany, targeting the general working population. | | |
| | Sample size: 264 | Mean age: 43.3 | Female ratio: 73.1% |
| **Intervention(s)** | Domain: Mental health and stress, working atmosphere | | |
| | Type: Mindfulness and relaxation, education and training opportunities | | |
| | The intervention was an internet-based, mobile-supported stress management program called "GET.ON Stress." It consisted of seven sessions focused on problem-solving and emotion regulation techniques, along with a refresher session. Participants received personalized feedback from an e-coach to support their progress. | | |
| | Duration: 2.5 months | | |
| | Setting: Digital (web/app-based) | | |
| **Comparator** | Waiting list control group (WLC) with unrestricted access to care as usual. | | |
| **Outcome** | Outcome measure(s): ROI, net benefit, cost-effectiveness ratio, cost-benefit ratio | | |
| | Self-reported data for cost-benefit analysis (6 months), ICER (6 months) for the employer (including intervention costs, costs due to absenteeism and presenteeism), cost-benefit analysis, symptoms | | |
| **Results** | The intervention showed a net benefit of €181 per participant in the first six months, with a benefit-cost ratio of 1.6. The ROI was €0.61 per euro invested. The ICER was -521 €, indicating that the intervention was more effective and cost-saving compared to usual care. | | |

**(38) Elson et al. (2019)**

| | | | |
|---|---|---|---|
| **Title** | Cost-benefit analysis of an employee assistance program for a geographically dispersed workforce in South Australia | | |
| **Country scope** | Australia | | |
| **Industry scope** | Consumer discretionary | | |
| **Study type** | Modeling | | |
| **JBI evaluation** | Risk of bias: Medium | | |
| **PICO framework** | | | |
| **Population** | Employees of the South Australian Department of Education, with 40.2% residing outside the Adelaide metropolitan area. | | |
| | Sample size: 1,984 | Mean age: N/A | Female ratio: N/A |
| **Intervention(s)** | Domain: Mental health and stress | | |
| | Type: Mindfulness and relaxation, mental health initiatives/training | | |
| | The South Australian Department of Education offers an Employee Assistance Program (EAP) with up to six fully funded counseling sessions per year, available through phone, in-person (in city areas), or online via Zoom. | | |
| | Duration: 12 months | | |
| | Setting: In-person, phone-based, digital (web/app-based) | | |
| **Comparator** | N/A | | |
| **Outcome** | Outcome measure(s): Cost-benefit ratio | | |
| | Primary: Registry-based cost-benefit ratio (including absenteeism and presenteeism estimates)<br>Secondary: Costs and benefits of the EAP, EAP utilization, and employee satisfaction | | |
| **Results** | The Employee Assistance Program (EAP) cost AUD 409.27 per user (AUD 14.26 per employee). By reducing teacher sick leave, the Ministry saved approximately AUD 96,787 in substitute teacher costs in 2018. The program reported a Cost-benefit ratio of 3.34, ranging from 2.84 to 3.84. | | |

**(39) Framke et al. (2016)**

| | | | |
|---|---|---|---|
| **Title** | Effect of a participatory organizational-level occupational health intervention on short-term sickness absence: a cluster randomized controlled trial | | |
| **Country scope** | Denmark | | |
| **Industry scope** | Consumer services | | |
| **Study type** | RCT | | |
| **JBI evaluation** | Risk of bias: Low | | |
| **PICO framework** | | | |
| **Population** | Pedagogical leaders, teachers, teaching assistants, and other staff employed at one of the 78 participating preschools in the municipality of Copenhagen between June 2011 and December 2013. | | |
| | Sample size: 2,576 | Mean age: 38.4 | Female ratio: 81.7% |
| **Intervention(s)** | Domain: Working atmosphere | | |
| | Type: Work climate change | | |
| | The "Pioneer Project" was a 25-month participative intervention designed to improve workplace conditions by focusing on core tasks and reducing unnecessary duties. It consisted of four phases: planning, development with employee input, implementation, and self-evaluation. Activities included workshops, change management training, advisory support, seminars for steering groups, training on workplace culture, and ongoing support from workplace environment advisors. | | |
| | Duration: 34 months | | |
| | Setting: In-person | | |
| **Comparator** | The control group included 34 preschools with 1,279 employees who did not receive any intervention. | | |
| **Outcome** | Outcome measure(s): Sick days | | |
| | Registry data on incidence rate of short-term sickness absence (≤14 calendar days) during a follow-up period of 29 months | | |
| **Results** | The intervention group had 8.68 days of short-term sickness absence per person-year, compared to 9.17 days in the control group. The rate ratio (RR) was 0.93 (95% CI: 0.86–1.00), indicating a significantly lower risk of short-term sickness absence in the intervention group. | | |

| (40) Freund et al. (2024) | | | |
|---|---|---|---|
| **Title** | A Universal Digital Stress Management Intervention for Employees: Randomized Controlled Trial with Health-Economic Evaluation | | |
| **Country scope** | Germany | | |
| **Industry scope** | N/A | | |
| **Study type** | RCT | | |
| **JBI evaluation** | Risk of bias: Low | | |
| **PICO framework** | | | |
| **Population** | Employees in Germany | | |
| | Sample size: 396 | Mean age: 41.76 | Female ratio: 76% |
| Intervention(s) | Domain: Mental health & stress, working atmosphere | | |
| | Type: Mindfulness and relaxation, education and training opportunities | | |
| | The GET.ON Stress digital stress management intervention included seven modules and a follow-up session, focusing on psychoeducation, problem-solving, emotion regulation, and future planning. Based on the transactional stress model, it featured interactive training, exercises, reports, and multimedia content. Homework, behavior planning, and an online diary helped integrate the new knowledge. Participants could also receive automatic text messages on their mobile phones. | | |
| | Duration: 6 months | | |
| | Setting: Digital (web/app-based) | | |
| **Comparator** | The control group was a waiting list control group (WLC). Participants in both study groups had full access to care as usual | | |
| **Outcome** | Outcome measure(s): Cost-benefit assessment (CBA), cost-effectiveness ratio, cost utility (CUA), net benefit, ROI | | |
| | Health outcome in the cost-effectiveness analysis (CEA) was the symptom-free status based on the Perceived Stress Scale (PSS) | | |
| **Results** | ROI: The intervention had a high probability (78%) of a positive ROI. The ROI was calculated at 77%. Net benefit: The net benefit per participant in the intervention group was €76. Cost effectiveness: The iSMI intervention had a 94% likelihood of being cost-effective compared to the waitlist control, based on a willingness-to-pay threshold of €1500 (US $1645.37) per additional symptom-free individual. Cost utility: The intervention showed a high probability of being cost-effective. With a willingness to pay of € 20,000 per QALY gained, the probability was 80% | | |

| (41) Geraedts et al. (2015) | | | |
|---|---|---|---|
| **Title** | Economic Evaluation of a Web-Based Guided Self-Help Intervention for Employees With Depressive Symptoms | | |
| **Country scope** | Netherlands | | |
| **Industry scope** | Financials, Industrials, Consumer Services, Not assignable | | |
| **Study type** | RCT | | |
| **JBI evaluation** | Risk of bias: Medium | | |
| **PICO framework** | | | |
| **Population** | Employees with increased depressive symptoms who were not on sick leave from six companies: two banks, two research organizations, a security company, and a university. Employees who scored 16 or more points on the Centre for Epidemiologic Studies Depression Scale were eligible to participate. | | |
| | Sample size: 231 | Mean age: 43.4 | Female ratio: 62.3% |
| Intervention(s) | Domain: Mental health and stress | | |
| | Type: Mindfulness and relaxation, mental health initiatives/training | | |
| | "Happy@Work" was a short, minimally guided self-help intervention combining problem-solving and cognitive therapy. It consists of six weekly lessons, each covering a different topic with information, examples, and tasks. Participants received feedback from a coach, a trained Master's student in clinical psychology, with all feedback reviewed by a supervisor to ensure treatment fidelity. | | |
| | Duration: 1.5 months | | |
| | Setting: Digital (web/app-based) | | |
| **Comparator** | Usual care which included an email with the result of the randomization and the advice to contact their family doctor, company doctor or a psychologist if necessary. | | |
| **Outcome** | Outcome measure(s): ROI, net benefit, cost-effectiveness ratio, cost-benefit-ratio, cost utility | | |
| | ROI calculation includes the intervention costs and the changes in the costs of absenteeism, presenteeism and company medical care | | |
| **Results** | The average ROI was 178% (95% CI: -2466 to 2863), with a 63% chance of positive financial return. The net benefit was €508 (95% CI: -7029 to 8039), and the BCR was 2.8 (CI: -25.7 to 27.6). The cost-effectiveness probability was 55% with a WTP of €0, rising to 95% at €3500 per CES-D point improvement. No significant effect on quality-adjusted life years was found. | | |

| (42) Goetzel et al. (2014) | | | |
|---|---|---|---|
| **Title** | Estimating the Return on Investment From a Health Risk Management Program Offered to Small Colorado-Based Employers | | |
| **Country scope** | United States | | |
| **Industry scope** | Not assignable | | |
| **Study type** | Quasi-Experiment | | |
| **JBI evaluation** | Risk of bias: High | | |
| **PICO framework** | | | |
| **Population** | Employees from small businesses in Colorado, USA, spanning various industries. | | |
| | Sample size: 2,458 | Mean age: 43 | Female ratio: 34% |
| **Intervention(s)** | Domain: Physical health and fitness, mental health and stress | | |
| | Type: Exercise programs, nutrition and weight management., health monitoring programs, mindfulness and relaxation | | |
| | Health Risk Management program, including annual employee health assessments capturing biometric and lifestyle data (diet, exercise, tobacco/alcohol use, stress). Employees received personalized wellness reports, unlimited health coaching via phone in English and Spanish, and access to online tools like videos, behavior change programs (e.g., smoking cessation, weight loss), and health articles. Employers received aggregated health reports identifying workforce risks and estimating productivity losses due to illness. | | |
| | Duration: 12 months | | |
| | Setting: In-person, phone-based, digital (web/app-based) | | |
| **Comparator** | N/A | | |
| **Outcome** | Outcome measure(s): ROI | | |
| | Self-reporting-based ROI, productivity, health status | | |
| **Results** | The analysis reveals a Return on Investment (ROI) of $2.03 per dollar invested, considering both medical cost savings and productivity gains. This ROI comprises $0.58 from medical cost reductions and $1.45 from improved productivity. Over a one-year period, total medical costs for employees amounted to $11.2 million. By addressing ten key health risk factors, cumulative savings were projected to reach $124,867 in medical costs. | | |

| (43) Gregson et al. (2023) | | | |
|---|---|---|---|
| **Title** | Worksite exercise intervention for hospital health care providers: Outcomes and resource utilization of a pilot study | | |
| **Country scope** | Denmark | | |
| **Industry scope** | Health care | | |
| **Study type** | Quasi-Experiment | | |
| **JBI evaluation** | Risk of bias: Medium | | |
| **PICO framework** | | | |
| **Population** | Hospital employees in a Danish hospital department including nurses, doctors, secretaries and care assistants. | | |
| | Sample size: 80 | Mean age: 44.4 | Female ratio: 81.3% |
| **Intervention(s)** | Domain: Physical health and fitness | | |
| | Type: Exercise programs | | |
| | The program consisted of two 30-minute sessions per week, incorporating aerobics, resistance training, and balance exercises. It was supervised by qualified trainers and followed the "intelligent training" concept, which was tailored to the individual needs and abilities of the participants. | | |
| | Duration: 3 months | | |
| | Setting: In-person | | |
| **Comparator** | Non-participants in the same department. | | |
| **Outcome** | Outcome measure(s): Sickness absence hours | | |
| | Registry data on sick hours (3 months), intervention costs, self-reported quality of life, commitment to work, social capital in the workplace | | |
| **Results** | There was no statistically significant difference in sickness-related absenteeism between the intervention group (those who participated in the training program) and the comparison group (non-participants). The mean difference in total sick days was 18.8 hours (60.5 hours in the intervention group vs. 79.2 hours in the comparison group, p = 0.64). For sickness-related absences during working hours, the mean difference was 8.3 hours (43.6 hours in the intervention group vs. 52.0 hours in the comparison group, p = 0.87). | | |

| (44) Groeneveld et al. (2011) | | | |
|---|---|---|---|
| **Title** | Cost-Effectiveness and Cost-Benefit of a Lifestyle Intervention for Workers in the Construction Industry at Risk for Cardiovascular Disease | | |
| **Country scope** | Netherlands | | |
| **Industry scope** | Industrials | | |
| **Study type** | Netherland | | |
| **JBI evaluation** | Risk of bias: Low | | |
| **PICO framework** | | | |
| **Population** | Construction workers aged 18 to 65 with an increased risk of cardiovascular disease (CVD). Participants that stated that they wanted to improve their physical activity or diet were included in the study. | | |
| | Sample size: 573 | Mean age: Not provided | Female ratio: 0% |
| Intervention(s) | Domain: Physical health and fitness | | |
| | Type: Exercise programs, nutrition and weight management | | |
| | Personalized lifestyle intervention including face to face counselling (3 sessions lasting 45 to 60 minutes), telephone counselling (4 sessions lasting 15 to 30 minutes), and brochures on healthy lifestyles and CVD. | | |
| | Duration: 6 months | | |
| | Setting: In-person, phone-based | | |
| **Comparator** | Regular care by the company doctor, which consisted of brief information about the CVD risk profile. | | |
| **Outcome** | Outcome measure(s): Cost savings | | |
| | Costs included trainer fees, handbook printing, consultation (in-person/phone), €15 reimbursement per session, and coordination expenses while benefits incl savings from reduced absenteeism, calculated at €264/day for employees and €216/day for workers. ICER calculated only for social perspective. | | |
| **Results** | Regarding cost savings the analysis revealed a net loss of € 254 (95% CI: -1070 to 1536) per employee for the employer. The ICER (from social perspective) was € 145 per kilogram of weight loss, meaning that the society would have to invest €145 to achieve an additional kilogram of weight loss compared to standard care. The intervention could be considered cost-effective if society was willing to pay €2000 for an extra kilogram of weight loss. | | |

| (45) Gubler et al. (2017) | |
|---|---|
| **Title** | Doing Well by Making Well: The Impact of Corporate Wellness Programs on Employee Productivity |
| **Country scope** | United States |
| **Industry scope** | Industrials |
| **Study type** | Quasi-Experiment |
| **JBI evaluation** | Risk of bias: Low |
| **PICO framework** | |
| **Population** | Production employees in five laundry plants of a large, independent industrial laundry company in the US, called LaundryCo. Managers and non-factory workers (e.g., salespeople) were excluded from the study.<br>Sample size: 111 / Mean age: N/A / Female ratio: N/A |
| Intervention(s) | Domain: Physical health & fitness<br>Type: Health monitoring programs<br>Voluntary and free-of charge company wellness program run by an external provider. Two components included in the program: (1) Participants of the program received a 15% reduction in the monthly insurance premium (~0.4% of wage). (2) The program included annual biometric screenings testing for 42 common health markers, comprehensive health surveys covering medical history and lifestyle behaviors, personalized health reports with results and recommendations, and nurse-led seminar providing individual feedback and referrals for abnormal results.<br>Duration: 36 months<br>Setting: In-person |
| **Comparator** | Employees at a fifth LaundryCo plant that did not participate in the wellness program due to a different insurance plan. |
| **Outcome** | Outcome measure(s): ROI<br>Registry data was used to estimate the ROI of the program by comparing the productivity increases with the program costs. |
| **Results** | Considering the increase in productivity among participants, the decrease in productivity among non-participants and the program costs, an ROI of 76.3% was calculated. The cost of the program was USD 240 per participating employee, including the cost of the external provider and lost working time. The program led to an average increase in productivity of around 4% among the participating employees ("compliers"). This corresponds to one additional working hour per month per employee. |

**(46) Hendriksen et al. (2016)**

| | |
|---|---|
| **Title** | Effectiveness of a Multilevel Workplace Health Promotion Program on Vitality, Health, and Work-Related Outcomes |
| **Country scope** | Netherlands |
| **Industry scope** | Financials |
| **Study type** | Quasi-Experiment |
| **JBI evaluation** | Risk of bias: Medium |
| **PICO framework** | |
| **Population** | Employees of a Dutch insurance company, mainly office staff, including management.<br>Sample size: 433 \| Mean age: 42.2 \| Female ratio: 52.9% |
| **Intervention(s)** | Domain: Physical health and fitness, working atmosphere<br>Type: Health monitoring programs, work climate change<br>The study introduced a multi-stage workplace health promotion program, which included management training to promote vitality, team workshops and discussions to motivate compliance, and personalized health assessments with coaching for individuals.<br>Duration: 5 months<br>Setting: In-person, phone-based, digital (web/app-based) |
| **Comparator** | N/A |
| **Outcome** | Outcome measure(s): Sickness absence rate<br>Primary: Registry data on sick days (Average percentage of sick leave), presenteeism, vitality, and performance<br>Secondary: Health and well-being outcomes, behavior, and work-life balance |
| **Results** | The intervention significantly reduced sickness absence, with a drop in the percentage of absent employees from 48.4% at baseline to 32.0% after 15 months, showing a long-term positive effect on sickness rates. |

| (47) Hengel et al. (2013) | | | |
|---|---|---|---|
| **Title** | The effectiveness of a construction worksite prevention program on work ability, health, and sick leave: results from a cluster randomized controlled trial | | |
| **Country scope** | Netherlands | | |
| **Industry scope** | Industrials | | |
| **Study type** | RCT | | |
| **JBI evaluation** | Risk of bias: Low | | |
| **PICO framework** | | | |
| **Population** | Participants were recruited from 15 departments of six Dutch construction companies specializing in house, commercial, or industrial building. Participation required workers to perform construction work. | | |
| | Sample size: 293 | Mean age: 42.8 | Female ratio: 0.8% |
| **Intervention(s)** | Domain: Physical health and fitness, mental health and stress | | |
| | Type: Exercise programs, mindfulness and relaxation | | |
| | The intervention included a physical component with two 30-minute training sessions with a physical therapist and a rest-break tool, and a mental component with two one-hour empowerment training sessions at the worksite. | | |
| | Duration: 6 months | | |
| | Setting: In-person | | |
| **Comparator** | No intervention. | | |
| **Outcome** | Outcome measure(s): Sick days (dichotomized) | | |
| | Primary: Ability to work, mental and physical health<br>Secondary outcomes: Registry data on sick days (6-month prevalence of no or short-term sick leave [0–5 days] and long-term sick leave [>6 days]) and occurrence of musculoskeletal symptoms | | |
| **Results** | In relative terms, the intervention group showed a lower 6-month and 12-month prevalence of long-term sick leave compared to the control group, although the differences were not statistically significant. | | |

| (48) Hengel et al. (2014) | | | |
|---|---|---|---|
| **Title** | Prevention Program at Construction Worksites Aimed at Improving Health and Work Ability Is Cost-Saving to the Employer: Results From an RCT | | |
| **Country scope** | Netherlands | | |
| **Industry scope** | Industrials | | |
| **Study type** | RCT | | |
| **JBI evaluation** | Risk of bias: Low | | |
| **PICO framework** | | | |
| **Population** | Construction workers from 15 departments of six companies specializing in house, commercial, or industrial construction, including those actively performing construction work. | | |
| | Sample size: 293 | Mean age: 42.8 | Female ratio: 0.8% |
| **Intervention(s)** | Domain: Physical health and fitness, mental health and stress | | |
| | Type: Exercise programs, mindfulness and relaxation | | |
| | The intervention included a physical component with two 30-minute individual training sessions with a physical therapist and a rest-break tool, and a mental component with two one-hour interactive empowerment training sessions at the worksite in a construction trailer. | | |
| | Duration: 6 months | | |
| | Setting: In-person | | |
| **Comparator** | The control group received usual practice, consisting only of the mandatory training required by Dutch companies for employees to obtain a health and safety certificate. | | |
| **Outcome** | Outcome measure(s): Sick days | | |
| | Primary: Registry and self-reported data on cost savings, net benefit, cost-benefit, ROI (difference between monetary benefits [sickness absence] and intervention costs [costs of intervention and costs due to productivity losses]), ability to work, physical and mental health<br>Secondary: Musculoskeletal symptoms | | |
| **Results** | The intervention yielded a positive financial return for the employer by reducing sickness absence costs. For every euro invested, 6.4€ were saved, with a net benefit of 641€ per employee and an ROI of 544%. The intervention group had 8.5 fewer sick days than the control group. This positive return remained consistent in sensitivity analyses considering presenteeism costs. | | |

| (49) Herman et al. (2008) | | | |
|---|---|---|---|
| **Title** | Cost-effectiveness of Naturopathic Care for Chronic Low Back Pain | | |
| **Country scope** | Not provided | | |
| **Industry scope** | Industrials | | |
| **Study type** | RCT | | |
| **JBI evaluation** | Risk of bias: Medium | | |
| **PICO framework** | | | |
| **Population** | Employees with chronic back pain. Participants included warehouse workers who were diagnosed with chronic back pain of at least 6 weeks duration and who were not on sick leave. | | |
| | Sample size: 70 | Mean age: 46.58 | Female ratio: 50% |
| Intervention(s) | Domain: Mental health and stress, physical health and fitness | | |
| | Type: Exercise programs, nutrition and weight management, mindfulness and relaxation | | |
| | The treatment included acupuncture, relaxation exercises, physical exercise, nutritional counselling, 30-minutes semi-weekly naturopathic treatment, and a back training brochure. | | |
| | Duration: 3 months | | |
| | Setting: In-person | | |
| **Comparator** | 30-minutes semi-weekly standardized physiotherapy education and a brochure on back training | | |
| **Outcome** | Outcome measure(s): ROI | | |
| | ROI was estimated by comparing the costs per day of absence avoided with the costs to the employer due to productivity losses | | |
| **Results** | The investment in naturopathic treatment led to a return on investment of 7.9 % over a period of 6 months. The positive ROI was driven by a reduction in days of absence. The cost per day of absence avoided was USD 154 which was lower compared to the 172 US dollars that the employer suffers per day of absence due to lost productivity. | | |

| (50) Hughes et al. (2007) | | | |
|---|---|---|---|
| **Title** | A Lifestyle-Based Weight Management Program Delivered to Employees of Health and Economic Outcomes | | |
| **Country scope** | United States | | |
| **Industry scope** | N/A | | |
| **Study type** | Quasi-Experiment | | |
| **JBI evaluation** | Risk of bias: High | | |
| **PICO framework** | | | |
| **Population** | The study focused on overweight or obese employees (average weight of 113 kg and BMI of 39 kg/m$^2$) from three Fortune 500 companies in the Pacific Northwest of the United States. Most participants were middle-aged men and women, with job roles that did not require significant physical activity. | | |
| | Sample size: 516 | Mean age: 39 | Female ratio: 65% |
| **Intervention(s)** | Domain: Physical health and fitness | | |
| | Type: Exercise programs, nutrition and weight management., health monitoring programs | | |
| | The employer-sponsored weight management program focused on physical activity, dietary modification, behavioral counseling, and medical monitoring. The intervention was based on the National Institutes of Health's clinical guidelines for weight management. | | |
| | Duration: 12 months | | |
| | Setting: In-person | | |
| **Comparator** | N/A | | |
| **Outcome** | Outcome measure(s): Sick days | | |
| | Self-reported data on sick days (3 months), physical parameters (e.g., BMI, weight, blood pressure), number of doctor visits, medication use | | |
| **Results** | The program led to a significant reduction in self-reported sick days, from an average of 3.1 at baseline to 1.1 at the end of the intervention (P = 0.04). Participants experienced an average weight loss of 5.4%, along with improvements in blood pressure, waist circumference, and BMI. Additionally, depressive symptoms, as measured by the Beck Depression Inventory, decreased by 57.1%. The average number of prescription medications per participant fell by 44%, resulting in estimated annual medication cost savings of $2,382 per participant. | | |

**(51) Iijima et al. (2013)**

| | | | |
|---|---|---|---|
| **Title** | Cost-benefit Analysis of Comprehensive Mental Health Prevention Programs in Japanese Workplaces: A Pilot Study | | |
| **Country scope** | Japan | | |
| **Industry scope** | N/A | | |
| **Study type** | Quasi-Experiment | | |
| **JBI evaluation** | Risk of bias: High | | |
| **PICO framework** | | | |
| **Population** | The study involved employees from 11 large companies (average size: 5,543 employees) that had already implemented mental health prevention programs. | | |
| | Sample size: 12,864 | Mean age: N/A | Female ratio: N/A |
| **Intervention(s)** | Domain: Mental health and stress | | |
| | Type: Mindfulness and relaxation, mental health initiatives/training | | |
| | The program followed Japanese health guidelines with three prevention levels: Primary (education and workplace improvements), Secondary (early detection via health checks and surveys), and Tertiary (support for reintegration through consultations and coordination). | | |
| | Duration: N/A | | |
| | Setting: N/A | | |
| **Comparator** | Companies with a ROI of more than one was compared to companies with a ROI of less than one. | | |
| **Outcome** | Outcome measure(s): ROI, net benefit | | |
| | Productivity and costs for sick pay | | |
| **Results** | The mental health prevention programs had an average ROI of 1.55, with seven of eleven companies exceeding an ROI of one. The average net benefit per employee was 6,921 yen. Companies with a higher ROI had 12 fewer sick days per employee and lower overall costs, emphasizing the financial advantages of comprehensive mental health strategies. | | |

| (52) Ikegami et al. (2010) | | | |
|---|---|---|---|
| **Title** | The Effects of a Mental Health Training Program for Manufacturing Company Managers | | |
| **Country scope** | Japan | | |
| **Industry scope** | Technology | | |
| **Study type** | Quasi-Experiment | | |
| **JBI evaluation** | Risk of bias: Medium | | |
| **PICO framework** | | | |
| **Population** | Employees at all levels in a Japanese electronics company, mainly technicians and engineers who worked in production or in research and development. | | |
| | Sample size: 1,420 | Mean age: 35.2 | Female ratio: 16% |
| Intervention(s) | Domain: Mental health and stress, working atmosphere | | |
| | Type: Work climate change, mental health initiatives/training | | |
| | The intervention was a mental health training program for managers, focusing on Active Listening (AL) to improve communication and support employees. It included role-plays and group discussions to enhance mutual understanding. Managers received feedback on their department's Brief Job Stress Questionnaire (BJSQ) results, with follow-up sessions offering further training, support, and counseling for participants. | | |
| | Duration: N/A | | |
| | Setting: In-person | | |
| **Comparator** | Baseline data prior to the interventions were used as a control | | |
| **Outcome** | Outcome measure(s): Sickness absence rate | | |
| | Absence rate measured as the number of employees on sick leave for more than one month due to mental health problems | | |
| **Results** | The number of employees who were on sick leave for longer than one month due to mental health problems halved in 2006 and 2007 compared to the years before 2005. After the implementation of the program, which included Active Listening Training, the values for "quantitative workload", "qualitative workload" and "physical demands" compared to the baseline. | | |

| (53) Jenny et al. (2011) | | | |
|---|---|---|---|
| **Title** | Projekt SWiNG – Schlussbericht der Evaluation | | |
| **Country scope** | Switzerland | | |
| **Industry scope** | Industrials, utilities, consumer goods, health care | | |
| **Study type** | Quasi-Experiment | | |
| **JBI evaluation** | Risk of bias: High | | |
| **PICO framework** | | | |
| **Population** | Employees in eight pilot companies in Switzerland that all differ in size, sector and language region. | | |
| | Sample size: 5,014 | Mean age: N/A | Female ratio: N/A |
| **Intervention(s)** | Domain: Mental health and stress, working atmosphere | | |
| | Type: Mindfulness and relaxation, work climate change | | |
| | SWiNG Project utilizes a multifaceted intervention strategy to address workplace stress and promote employee health. Key elements of the intervention include S-Tool assessment, stress management courses, leadership training, and the formation of collaborative groups. | | |
| | Duration: N/A | | |
| | Setting: Digital (web/app-based), in-person | | |
| **Comparator** | People who have not taken part in the stress management course | | |
| **Outcome** | Outcome measure(s): ROI | | |
| | Economic effectiveness of SWiNG including ROI calculation | | |
| **Results** | ROI of 0.53 at 1 year post intervention end. Approximately 3 years after intervention end ROI of ~1. Overall, employees showed a positive impact on participants' resource-to-strain ratio, health outcomes, work performance. Beyond individual-level improvements, SWiNG catalyzed significant organizational changes including the creation of leadership forums, integration of health-related content into existing training programs, and modification of meeting agendas to address well-being concerns. | | |

| (54) Jorgensen et al. (2011) | | | |
|---|---|---|---|
| **Title** | Effects on musculoskeletal pain, work ability and sickness absence in a 1-year randomized controlled trial among cleaners | | |
| **Country scope** | Denmark | | |
| **Industry scope** | Industrials | | |
| **Study type** | RCT | | |
| **JBI evaluation** | Risk of bias: Low | | |
| **PICO framework** | | | |
| **Population** | Cleaning staff from nine different cleaning companies in Denmark. Participants needed to be employed at least 20 hours/week, work mainly during the day, and with the main task being cleaning. | | |
| | Sample size: 363 | Mean age: 45 | Female ratio: 100% |
| Intervention(s) | Domain: Physical health and fitness | | |
| | Type: Exercise programs | | |
| | Two interventions: (1) Physical coordination training (PCT) and cognitive behavioral training (CBTr). The PCT consisted of weekly 20-minute sessions at the workplace under the guidance of a trainer during the first 3 months. The training focused on intensive exercises to activate the stabilizing muscles around the trunk and shoulder girdle. (2) The CBT consists of group sessions led by a trained group leader twice a month for 2 hours at the workplace during the first 3 months. The training included group discussions on pain-related dysfunctional attitudes, coping and management, as well as the promotion of functional. | | |
| | Duration: 12 months | | |
| | Setting: Phone-based | | |
| **Comparator** | The comparison group received a one-hour health check (incl. lung function test and an aerobic capacity test). | | |
| **Outcome** | Outcome measure(s): Sick days | | |
| | Sick days were measured as median sickness absence days at follow-up (12 months) | | |
| **Results** | No significant effects of CBT or PCT on the ability to work or sick leave were found. The reference group had median sickness absence days of 2 at follow-up, meanwhile the PCT and CBT group showed 4.25 and 3.0 median days respectively. | | |

**(55) Kapinos et al. (2015)**

| | | | |
|---|---|---|---|
| **Title** | Does Targeting Higher Health Risk Employees or Increasing Intervention Intensity Yield Savings in a Workplace Wellness Program? | | |
| **Country scope** | United States | | |
| **Industry scope** | N/A | | |
| **Study type** | Cohort study | | |
| **JBI evaluation** | Risk of bias: Low | | |
| **PICO framework** | | | |
| **Population** | Employees in a large US company at increased risk of developing chronic diseases. The study focused on employees who were more actively engaged in the program. | | |
| | Sample size: 20,251 | Mean age: 37.56 | Female ratio: 39.15% |
| **Intervention(s)** | Domain: Physical health and fitness, mental health and stress | | |
| | Type: Exercise programs, nutrition and weight management, mindfulness and relaxation | | |
| | The wellness program included chronic condition management and lifestyle promotion. Employees completed a 20-minute Health Risk Assessment (HRA), which led to tailored coaching and support. High-risk employees received intensified coaching to address obesity, hypertension, diabetes, and high cholesterol. | | |
| | Duration: N/A | | |
| | Setting: In-person, phone-based, digital (web/app-based) | | |
| **Comparator** | No intervention. | | |
| **Outcome** | Outcome measure(s): Cost savings | | |
| | Third-party reported healthcare costs (prescription drugs, inpatient and outpatient costs) | | |
| **Results** | There was no evidence that the pre-disease program targeting employees at increased risk of chronic diseases led to cost savings. However, participants in the lifestyle management program who attended five or more sessions per year had about $20 lower medical costs compared to matched peers. Overall, the findings suggest that the preventative components of workplace wellness programs do not significantly reduce healthcare costs. | | |

**(56) Karlsson et al. (2024)**

| | |
|---|---|
| **Title** | Is a Problem-Solving Intervention with Workplace Involvement for Employees on Sickness Absence Due to Common Mental Disorders More Effective, than Care as Usual, in Reducing Sickness Absence Days? Results of a Cluster-Randomised Controlled Trial in Primary Health Care |
| **Country scope** | Sweden |
| **Industry scope** | Not specified |
| **Study type** | RCT |
| **JBI evaluation** | Risk of bias: Low |
| **PICO framework** | |
| **Population** | Leaders and employees aged 18 to 59 who were on sick leave for 2 to 12 weeks, primarily due to a diagnosis of mild to moderate depression, anxiety disorder, or adjustment disorder.<br>Sample size: 197 \| Mean age: 42 \| Female ratio: 85% |
| **Intervention(s)** | Domain: Working atmosphere<br>Type: Education & training opportunities<br>The PSI-WPI intervention involved rehabilitation coordinators delivering a structured problem-solving approach in 2–5 sessions, starting one week after study entry. It followed five steps: employee assessment, manager consultation, solution brainstorming, action plan creation, and a joint meeting for finalization and implementation.<br>Duration: Not specified<br>Setting: In-person, phone based |
| **Comparator** | Care as usual (CAU), which typically includes cognitive behavioral therapy or antidepressants. |
| **Outcome** | Outcome measure(s): Sick days<br>Registry data (18 months) |
| **Results** | The authors found no significant reduction in sickness absence for the intervention. At month 12, the mean absence was 3.2 days for CAU and 5.7 days for the intervention group, with a mean difference of 2.5 days (95% CI: 0.95–3.27). Over the study period, the estimated mean difference was 2.24 days (95% CI: 1.85–2.64), suggesting that PSI-WPI was not more effective than CAU in reducing sick days. |

| **(57) Keus van de Poll et al. (2020)** | | | |
|---|---|---|---|
| **Title** | Preventing sickness absence among employees with common mental disorders or stress-related symptoms at work: a cluster randomised controlled trial of a problem-solving-based intervention conducted by the Occupational Health Services | | |
| **Country scope** | Sweden | | |
| **Industry scope** | Not specified | | |
| **Study type** | RCT | | |
| **JBI evaluation** | Risk of bias: Low | | |
| **PICO framework** | | | |
| **Population** | Employees with general mental disorders or work-related stress symptoms with less than 3 months of sick leave due to common mental disorders (CMD). | | |
| | Sample size: 100 | Mean age: 43.33 | Female ratio: 80% |
| **Intervention(s)** | Domain: Working atmosphere | | |
| | Type: Education & training opportunities | | |
| | OHS consultants received one day of training to support work adjustments and stress management, addressing six workplace factors (workload, control, reward, community, fairness, values) using problem-solving techniques. The intervention included interviews, a joint meeting, and at least three follow-ups over three months. | | |
| | Duration: 1.5 months | | |
| | Setting: In-person | | |
| **Comparator** | OHS consultants in the CAU group received a one-hour introduction to psychosocial and mental health factors, without the same theoretical framework as the intervention group. | | |
| **Outcome** | Outcome measure(s): Sick days | | |
| | Primary: Registry data (12 months)<br>Secondary: Self-reported sick days every 4 weeks, RTW and production loss | | |
| **Results** | The PSI group showed a significant reduction in sick days, particularly from months 5 to 8, with a total difference of 30.68 days over 12 months compared to CAU. While the PSI group also improved in mental health and earlier partial return to work, these changes were not statistically significant. The mean effect size was -2.59 (CI: -3.96 to -1.21). | | |

| (58) Keus van de Poll et al. (2020) | | | |
|---|---|---|---|
| **Title** | Cost-Effectiveness of a Problem-Solving Intervention Aimed to Prevent Sickness Absence among Employees with Common Mental Disorders or Occupational Stress | | |
| **Country scope** | Sweden | | |
| **Industry scope** | Not specified | | |
| **Study type** | RCT | | |
| **JBI evaluation** | Risk of bias: Low | | |
| **PICO framework** | | | |
| **Population** | Workers who sought support from one of three occupational health services due to work-related stress or CMD symptoms affecting their ability to work. | | |
| | Sample size: 100 | Mean age: 43.33 | Female ratio: 80% |
| **Intervention(s)** | Domain: Working atmosphere | | |
| | Type: Education and training opportunities | | |
| | OHS consultants received one day of training to support work adjustments and stress management, addressing six workplace factors (workload, control, reward, community, fairness, values) using problem-solving techniques. The intervention included interviews, a joint meeting, and at least three follow-ups over three months. | | |
| | Duration: Not specified | | |
| | Setting: Digital, in-person | | |
| **Comparator** | OHS consultants in the CAU group received a one-hour introduction to psychosocial and mental health factors, without the same theoretical framework as the intervention group. | | |
| **Outcome** | Outcome measure(s): Net benefit, Cost-benefit (CBA), Cost-effectiveness ratio | | |
| | Other outcomes measures included sick days (12 months) - see also study number 57 | | |
| **Results** | The study found that PSI recipients had at least 15 fewer long-term sick leave days over one year compared to CAU. Cost-effectiveness was not demonstrated from the employer's perspective, but PSI was cost-beneficial from a societal standpoint. | | |

| **(59) Klasen et al. (2021)** | | | |
|---|---|---|---|
| **Title** | Efficacy of an indicated prevention strategy on sickness absence and termination of the employment contract: a 5-year follow-up study | | |
| **Country scope** | Netherlands | | |
| **Industry scope** | Not specified | | |
| **Study type** | RCT | | |
| **JBI evaluation** | Risk of bias: Low | | |
| **PICO framework** | | | |
| **Population** | Employees at high risk of future long-term sickness absence (LTSA) with mild depressive symptoms were included. Depressive symptoms were assessed using the Hospital Anxiety and Depression Scale (HAD-D). | | |
| | Sample size: 139 | Mean age: 47.7 | Female ratio: 38.9% |
| **Intervention(s)** | Domain: Mental health | | |
| | Type: Mental health initiatives | | |
| | The intervention group received psychological treatment based on problem-solving therapy (PST) and cognitive-behavioral therapy (CBT). Employees were offered up to seven 45-minute individual sessions, with homework reviewed in each session, extendable to 13 if needed. Ten psychologists, trained through a two-day pre-intervention program and a one-day booster during the study, facilitated the sessions. | | |
| | Duration: Not specified | | |
| | Setting: In-person | | |
| **Comparator** | The control groups in both RCTs received usual care, meaning support was provided only when requested. | | |
| **Outcome** | Outcome measure(s): Sick days | | |
| | Primary: Registry data on sick days (12 months)<br>Secondary: Termination of the employment relationship | | |
| **Results** | RCT 2 found no significant differences in sickness absence duration or frequency between groups after 12 months. After 3–5 years, the control group had fewer sick days, but the difference was uncertain and not statistically significant in ITT and PP analyses. | | |

**(60) Kobayashi et al. (2008)**

| | | | |
|---|---|---|---|
| **Title** | Effects of a Worker Participatory Program for Improving Work Environments on Job Stressors and Mental Health among Workers: A Controlled Trial | | |
| **Country scope** | Japan | | |
| **Industry scope** | Industrials | | |
| **Study type** | Controlled Trial | | |
| **JBI evaluation** | Risk of bias: Medium | | |
| **PICO framework** | | | |
| **Population** | Office workers in a large steel manufacturing company, excluding departments that worked in shifts. | | |
| | Sample size: 1,070 | Mean age: 41.9 | Female ratio: 11% |
| **Intervention(s)** | Domain: Working atmosphere | | |
| | Type: Work climate change | | |
| | The participatory program aimed to improve the working environment using the Mental Health Action Checklist for a Better Workplace Environment. The intervention included the following steps: forming a team to improve the working environment, creating a work stress profile, planning workshops, and implementing action plans. | | |
| | Duration: 6 months | | |
| | Setting: In-person | | |
| **Comparator** | No intervention. | | |
| **Outcome** | Outcome measure(s): Sick days (dichotomized) | | |
| | Registry data on sick days (0 or ≥1), psychological distress and health risks | | |
| **Results** | The intervention had no significant effect on sick days over one year, with the following reasons provided: low frequency of sick days, the follow-up measurement covering 12 months during which the measures were still being implemented, and a small sample size for women, particularly in the intervention group. | | |

**(61) Krampen (2010)**

| | | | |
|---|---|---|---|
| **Title** | Evaluation of a Program on Systematic Self-Monitoring and Reflection of Health Behavior in Organisations: Results of Two Randomised Controlled Studies on Well-Being and Absenteeism of Employees and Skilled Workers (Study 1) | | |
| **Country scope** | Germany | | |
| **Industry scope** | Industrials | | |
| **Study type** | RCT | | |
| **JBI evaluation** | Risk of bias: High | | |
| **PICO framework** | | | |
| **Population** | Employees from the public service of a state administrative organization, selected due to increasing absenteeism. | | |
| | Sample size: 56 | Mean age: 41.4 | Female ratio: 47% |
| **Intervention(s)** | Domain: Physical health and fitness, mental health, working atmosphere | | |
| | Type: Exercise programs, nutrition and weight management, mindfulness and relaxation, work climate change | | |
| | The intervention in both studies is the SySeRe program, which focuses on systematic self-observation and reflection on health behavior, encouraging participants to independently find ways to improve their health and attitudes. The program is conducted in small groups of up to 15 participants, who meet weekly for 90 to 120 minutes. It covers six areas of life and behavior: eating and drinking habits (including alcohol), drug and tobacco use (including medications), physical activity, media consumption, social relationships, and stress reactions and coping. | | |
| | Duration: 2 months | | |
| | Setting: In-person | | |
| **Comparator** | Waiting list control group | | |
| **Outcome** | Outcome measure(s): Sick days | | |
| | Registry data on sick days (12 months) as well as psychosomatic complaints, subjective well-being, personal self-regulation of development | | |
| **Results** | In Study 1, the intervention group showed 1.9 days of absenteeism, while the control group had 7.3 days, resulting in a mean difference of 5.4 fewer sick days during the follow-up period (last 2 months). | | |

| **(62) Larsen et al. (2019)** | |
|---|---|
| **Title** | The effect of strengthening health literacy in nursing homes on employee pain and consequences of pain – a stepped-wedge intervention trial |
| **Country scope** | Denmark |
| **Industry scope** | Health care |
| **Study type** | Quasi-Experiment |
| **JBI evaluation** | Risk of bias: Low |
| **PICO framework** | |
| **Population** | All types of nursing home staff. Nursing assistants who were either trained social and health care assistants or assistants with 1-2 years of vocational training. Managers were primarily registered nurses and carers. Other employees included kitchen, cleaning and technical staff. |
| | Sample size: 405 / Mean age: 47 / Female ratio: 89% |
| Intervention(s) | Domain: Working atmosphere |
| | Type: Education and training opportunities |
| | Organizational intervention to strengthen health literacy in the workplace in nursing homes. The intervention consisted of two elements: (1) Training for staff and management to create knowledge on pain prevention and management with communication tools. (2) Structured dialogues between staff and supervisors to develop action plans to prevent and reduce pain. |
| | Duration: 6 months |
| | Setting: N/A |
| **Comparator** | The comparison group consisted of the same employees before the intervention |
| **Outcome** | Outcome measure(s): Sick days |
| | Sick days measures with self-reporting survey every 4-week capturing the number of days (0-28) participants were absent from work due to pain in muscles and joints |
| **Results** | Intervention showed no significant overall effect on the number of sick days taken by employees. The reduction in sick days by -0.22 days (95% CI -0.57 0.14) per month was observed but was not statistically significant. Sensitivity analysis focusing on employees with an initial pain level of >3 showed a significant reduction in sick days by 0.65 days (95% CI -1.23-0.07). |

**(63) Lerner et al. (2020)**

| | | | |
|---|---|---|---|
| **Title** | Effect of Adding a Work-Focused Intervention to Integrated Care for Depression in the Veterans Health Administration a Randomized Clinical Trial | | |
| **Country scope** | United States | | |
| **Industry scope** | N/A | | |
| **Study type** | RCT | | |
| **JBI evaluation** | Risk of bias: Low | | |
| **PICO framework** | | | |
| **Population** | Veterans aged 18 years or older with at least 15 working hours per week in a job held for at least 6 months suffering from work-related restrictions and a current major depressive disorder or a persistent depressive disorder. | | |
| | Sample size: 253 | Mean age: 45.7 | Female ratio: 14.8% |
| **Intervention(s)** | Domain: Mental health and stress | | |
| | Type: Mental health initiatives/training | | |
| | Participants received integrated care (IC) and the "Be Well at Work" (BWAW) program. IC included depression treatment through screening, clinical informatics, measurement-based care, and referrals to psychiatric services. BWAW provided eight biweekly telephone sessions, followed by a follow-up call, with PhD-level psychologists helping patients address work-related barriers and apply cognitive-behavioral strategies and job modifications. | | |
| | Duration: 4 months | | |
| | Setting: Phone-based | | |
| **Comparator** | Only integrated care (IC) without work-related counselling (BWAW). | | |
| **Outcome** | Outcome measure(s): ROI | | |
| | Primary: Self-reporting-based ROI (24 months), productivity<br>Secondary: Symptoms development | | |
| **Results** | The ROI was 160% when both productivity improvements and reduced absenteeism were considered at the 4-month follow-up. When only productivity gains were accounted for, the ROI was 30%. Over a 2-year period, the ROI increased to 421% when factoring in both absenteeism and productivity, and 160% when excluding productivity gains. | | |

**(64) Linden et al. (2014)**

| | | | |
|---|---|---|---|
| **Title** | Reduction of sickness absence by an occupational health care management program focusing on self-efficacy and self-management | | |
| **Country scope** | Germany | | |
| **Industry scope** | Consumer services | | |
| **Study type** | Quasi-Experiment | | |
| **JBI evaluation** | Risk of bias: Medium | | |
| **PICO framework** | | | |
| **Population** | Employees of a department within the German Federal Pension Insurance. | | |
| | Sample size: 509 | Mean age: N/A | Female ratio: N/A |
| **Intervention(s)** | Domain: Physical health and fitness, mental health, working atmosphere | | |
| | Type: Exercise programs, Nutrition and weight management, Mindfulness and relaxation, Work climate change | | |
| | The intervention included various courses and public lectures on general health topics, with 69% attendance for workplace reviews, 15.7% for nutrition courses, 5.7% for gymnastics and dance, 5% for stress management, and 5% for laughter courses. A key component was focus groups with nine teams (55% attendance) held outside regular work hours at a rehabilitation center, discussing workspace, tasks, colleagues, support needs, and resources. | | |
| | Duration: Not specified | | |
| | Setting: In-person | | |
| **Comparator** | The comparator group included two departments of German Federal Pension Insurance, which had similar organization and working methods to the intervention department but did not participate in the OHMP. | | |
| **Outcome** | Outcome measure(s): Sickness absence rate | | |
| | Registry data on sick day rate (12 months) | | |
| **Results** | In the year following the intervention, sickness absence in the intervention group decreased from 9.26% to 7.93%, while it increased from 7.9% to 10.7% in the two control groups. | | |

| (65) Losina et al. (2017) | | | |
|---|---|---|---|
| **Title** | Physical activity and unplanned illness-related work absenteeism: Data from an employee wellness program | | |
| **Country scope** | United States | | |
| **Industry scope** | Health care | | |
| **Study type** | Cohort study | | |
| **JBI evaluation** | Risk of bias: Medium | | |
| **PICO framework** | | | |
| **Population** | Employees of a Dutch insurance company, mainly office staff, including management. | | |
| | Sample size: 292 | Mean age: 38.5 | Female ratio: 83% |
| **Intervention(s)** | Domain: Physical health and fitness | | |
| | Type: Exercise programs | | |
| | The program aimed at increasing physical activity, where participants used a fitness tracker. Teams of three earned financial rewards for meeting weekly activity goals (e.g., +10% compared to the previous week). | | |
| | Duration: 6 months | | |
| | Setting: In-person, digital (web/app-based) | | |
| **Comparator** | The study compared groups based on weekly activity levels: low (0-74 minutes), medium (75-149 minutes), and those meeting CDC guidelines (≥150 minutes/week). | | |
| **Outcome** | Outcome measure(s): Sickness absence hours | | |
| | Self-reported data on sick hours (6 months) | | |
| **Results** | The study found that lower physical activity was linked to more sickness-related absences. Sick leave decreased as physical activity increased: 5 hours for ≥150 minutes/week, 11 hours for 75–149 minutes/week, and 19 hours for 0–74 minutes/week. After adjusting for factors like age, race, education, and chronic conditions, those in the middle activity group were 2.4 times more likely to take unplanned sick leave, while those in the lowest group were 3.5 times more likely, compared to the most active group. | | |

| (66) Makrides et al. (2011) | | | |
|---|---|---|---|
| **Title** | The Healthy Lifeworks Project. A Pilot Study of the Economic Analysis of a Comprehensive Workplace Wellness Program in a Canadian Government Department | | |
| **Country scope** | Canada | | |
| **Industry scope** | Not assignable | | |
| **Study type** | Cohort study | | |
| **JBI evaluation** | Risk of bias: Medium | | |
| **PICO framework** | | | |
| **Population** | Employees of the Department of Justice (DOJ) of the Public Service of Nova Scotia. | | |
| | Sample size: 402 | Mean age: 44.9 | Female ratio: 61% |
| **Intervention(s)** | Domain: Physical health and fitness | | |
| | Type: Exercise programs, nutrition and weight management, health monitoring programs | | |
| | The workplace wellness program included health risk assessments, motivation contests, incentive programs, lifestyle coaching, workshops, and health fairs. It also introduced workplace changes like healthier cafeteria options, healthy meeting snacks, walking paths, and wellness spaces. | | |
| | Duration: N/A | | |
| | Setting: In-person, phone-based, digital (web/app-based) | | |
| **Comparator** | N/A | | |
| **Outcome** | Outcome measure(s): Sick days (dichotomized) | | |
| | Registry data on sick days (short-term and general illnesses), drug costs, cost savings, and wellness-Scores | | |
| **Results** | Employees who maintained a low health risk (0-2 risk factors) showed a slight 4% decrease in absenteeism. Maintaining a low-risk status helped prevent an increase in costs related to short-term and generalized illnesses (STI and GI). However, no causal relationship between the intervention and absenteeism was proven. Estimated cost savings were 248 Canadian dollars per employee per year for each reduction in health risk. | | |

| (67) Meenan et al. (2010) | | | |
|---|---|---|---|
| **Title** | Economic Evaluation of a Worksite Obesity Prevention and Intervention Trial among Hotel Workers in Hawaii | | |
| **Country scope** | United States | | |
| **Industry scope** | Consumer services | | |
| **Study type** | RCT | | |
| **JBI evaluation** | Risk of bias: High | | |
| **PICO framework** | | | |
| **Population** | Overweight and obese employees (BMI > 25) working in hotels on Oahu, Hawaii. The sample included a diverse ethnic composition, representative of the Hawaiian population. | | |
| | Sample size: 6,958 | Mean age: N/A | Female ratio: N/A |
| **Intervention(s)** | Domain: Physical health and fitness | | |
| | Type: Nutrition and weight management | | |
| | The "Work, Weight, and Wellness" (3W) program was a two-year weight loss initiative. Level 1 focused on awareness through assessments and feedback, while Level 2 added on-site weight management groups, environmental initiatives, and support in the final year. | | |
| | Duration: 24 months | | |
| | Setting: In-person | | |
| **Comparator** | Minimal intervention (Level 1) | | |
| **Outcome** | Outcome measure(s): Net present value | | |
| | Primary: BMI and Waist to height ratio<br>Secondary: Self- and third-party reporting-based ROI and net present value (including medical costs, absenteeism, productivity, and employee fluctuation) | | |
| **Results** | Both intervention levels resulted in negative NPVs over 24 months. Stage 1 (minimal) had an NPV of -$341,996, while Stage 2 (intensive) had an NPV of -$1,173,961. The impact on sick days was inconsistent, based on self-reported data, and no clear positive effect was demonstrated. | | |

**(68) Milani et al. (2009)**

| | | | |
|---|---|---|---|
| **Title** | Impact of Worksite Wellness Intervention on Cardiac Risk Factors and One-Year Health Care Costs | | |
| **Country scope** | United States | | |
| **Industry scope** | N/A | | |
| **Study type** | RCT | | |
| **JBI evaluation** | Risk of bias: Medium | | |
| **PICO framework** | | | |
| **Population** | Employees and spouses of a single employer. | | |
| | Sample size: 339 | Mean age: 40 | Female ratio: 52.4% |
| **Intervention(s)** | Domain: Physical health and fitness, mental health and stress | | |
| | Type: Exercise programs, nutrition and weight management., health monitoring programs, mindfulness and relaxation | | |
| | The six-month Risk Assessment and Modification Program (RAMP) by Ochsner Health System included workplace health education, smoking cessation support, stress management, medical referrals, addiction treatment, and fitness center access. | | |
| | Duration: 6 months | | |
| | Setting: In-person | | |
| **Comparator** | The control group received usual healthcare without RAMP-specific interventions but had access to exercise opportunities during lunch breaks, similar to the intervention group. | | |
| **Outcome** | Outcome measure(s): ROI, cost savings | | |
| | Registry data on medical costs (total cost of health insurance claims determined for each participant 1 year post intervention) and health outcomes | | |
| **Results** | Over 12 months post-intervention, medical claim costs averaged $1,539 per participant in the intervention group (p = .002) and $2,522 in the control group (not significant), with a statistically significant cost difference (p = 0.01). The intervention generated $6 in healthcare savings for every $1 invested. | | |

**(69) Musich et al. (2015)**

| | | | |
|---|---|---|---|
| **Title** | An Evaluation of the Well at Dell Health Management Program: Health Risk Change and Financial Return on Investment | | |
| **Country scope** | United States | | |
| **Industry scope** | Technology | | |
| **Study type** | Quasi-Experiment | | |
| **JBI evaluation** | Risk of bias: Low | | |
| **PICO framework** | | | |
| **Population** | Employees of the company Dell who had continuous health insurance coverage between 2010 and 2011. | | |
| | Sample size: 24,651 | Mean age: 42 | Female ratio: 27% |
| Intervention(s) | Domain: Physical health and fitness, working atmosphere | | |
| | Type: Health monitoring programs, education and training opportunities | | |
| | "Well at Dell" health management program, which consists of three core components: (1) Health risk assessment (HRA) / Wellness, (2) Lifestyle-Management-Coaching (incl. telephone coaching), and (3) Disease-Management-Coaching (incl. employees who suffered from diabetes, heart disease and/or back pain). Intervention consisted of year-round multi-channel communications and campaigns. | | |
| | Duration: 12 months | | |
| | Setting: Digital (web/app-based), phone-based, in-person | | |
| **Comparator** | Difference-in-Difference method with people that did not participate in the respective programs. | | |
| **Outcome** | Outcome measure(s): ROI, net benefit | | |
| | For each program changes in health expenditure per member per year before and after participation between participants and non-participants used for economic calculation. | | |
| **Results** | ROI of 2.48 with an average program investment of USD 91.46 per participant. The savings resulted in a net profit of USD 135.17 per employee. | | |

**(70) Noben et al. (2014)**

| | |
|---|---|
| **Title** | Protecting and Promoting Mental Health of Nurses in the Hospital Setting: Is it cost-effective from an employer's perspective |
| **Country scope** | Netherlands |
| **Industry scope** | Health care |
| **Study type** | RCT |
| **JBI evaluation** | Risk of bias: Low |
| **PICO framework** | |
| **Population** | Nurses in an academic hospital in the Netherlands who are at increased risk of mental health problems.<br>Sample size: 413 · Mean age: 42.19 · Female ratio: 79.7% |
| Intervention(s) | Domain: Mental health and stress<br>Type: Mental health initiatives/training<br>The intervention includes screening for work ability impairments and six mental health issues: stress, fatigue, drinking, depression, anxiety, and PTSD. Participants receive personalized feedback, and those with positive results are referred to an occupational physician. The physicians follow a seven-step protocol to improve work ability and well-being, supported by a three-hour training on protocol implementation.<br>Duration: 6 months<br>Setting: Digital (web/app-based), in-person |
| **Comparator** | Screening without feedback or referral to the company doctor |
| **Outcome** | Outcome measure(s): ROI, net benefit, cost-benefit-analysis (CBA)<br>Economic evaluation looks at a period of 6 months (without discontinuation of costs and benefits given short 6-month period) |
| **Results** | For every euro invested in the intervention, a profit of 11 € was realized. Sensitivity analyses for ROI ranging from 5 to 11 €. Net savings of 244 € per nurse (employee) were realized when only reduced absenteeism was included. In case higher productivity (inclusion of presenteeism) was taken into account a net benefit of 651 € was realized and statistically significant (p = 0.008). The cost-benefit ratio was calculated at 0.09 €. |

**(71) Norwitz et al. (2022)**

| | | | |
|---|---|---|---|
| **Title** | A Company Is Only as Healthy as Its Workers: A 6-Month Metabolic Health Management Pilot Program Improves Employee Health and Contributes to Cost Savings | | |
| **Country scope** | United States | | |
| **Industry scope** | N/A | | |
| **Study type** | Quasi-Experiment | | |
| **JBI evaluation** | Risk of bias: High | | |
| **PICO framework** | | | |
| **Population** | Employees of a manufacturing company with obesity, prediabetes, or diabetes. All participants had a BMI over 35 kg/m² at baseline and were selected based on their perceived medical need. | | |
| | Sample size: 9 | Mean age: 52.9 | Female ratio: 56% |
| **Intervention(s)** | Domain: Physical health and fitness | | |
| | Type: Exercise programs, nutrition and weight management | | |
| | The intervention included a ketogenic diet, regular virtual meetings, continuous glucose monitoring, and access to an app with educational content, live streams, group meetings, and a community chat. | | |
| | Duration: 6 months | | |
| | Setting: Digital (web/app-based) | | |
| **Comparator** | N/A | | |
| **Outcome** | Outcome measure(s): Cost savings | | |
| | Third-party reporting-based cost savings, weight, metabolic health, and 10-year risk of cardiovascular events | | |
| **Results** | Improved metabolic health through the program allowed many participants to reduce or discontinue certain medications. Based on drug list prices, total savings over 24 weeks were estimated at USD 45,171.70, averaging ~USD 5,437.33 per participant. Significant health improvements were observed, including an average weight reduction of 17.4 kg. | | |

| (72) Ornek et al. (2020) | | | |
|---|---|---|---|
| **Title** | Effects of a work-related stress model based mental health promotion program on job stress, stress reactions and coping profiles of women workers: a control groups study | | |
| **Country scope** | Turkey | | |
| **Industry scope** | Consumer Discretionary | | |
| **Study type** | Quasi-Experiment | | |
| **JBI evaluation** | Risk of bias: Low | | |
| **PICO framework** | | | |
| **Population** | Female textile workers with high work-related stress (WRS) were included in the study if they had a job-stress subscale score above 45. Participants who used medication affecting cortisol or IgA were excluded. | | |
| | Sample size: 70 | Mean age: 32.3 | Female ratio: 100% |
| **Intervention(s)** | Domain: Mental health and stress | | |
| | Type: Mindfulness and relaxation | | |
| | The "Work-ProMentH" program, based on the work stress model, included stress management techniques, coping strategies, relaxation exercises, and education on stress physiology, reactions, and related illnesses. The intervention was delivered through a 45-minute session, followed by 12 weeks of reinforcement via reminders, videos, and WhatsApp messages. | | |
| | Duration: 3 months | | |
| | Setting: In-person, phone-based, digital (web/app-based) | | |
| **Comparator** | Women from another textile factory without intervention. | | |
| **Outcome** | Outcome measure(s): Sickness absence hours | | |
| | Primary: Self-reported and registry data on sick hours (3 months), work-related stress and stress reactions, social support, performance, and coping strategies<br>Secondary: Biological stress markers such as salivary immunoglobulin A (S-IgA) and cortisol (S-Cortisol) | | |
| **Results** | Participants in the Work-ProMentH program experienced a significant reduction in absenteeism ($p = .029$; $\eta p^2 = .09$) compared to the control group. Their average hours of absence per month decreased from 12.85 (SD = 11) to 7.65 (SD = 8.17), whereas absenteeism in the control group increased over the study period. | | |

| (73) Proper et al. (2004) | | | |
|---|---|---|---|
| **Title** | Costs, benefits and effectiveness of worksite physical activity counseling from the employer's perspective | | |
| **Country scope** | Netherlands | | |
| **Industry scope** | Not assignable | | |
| **Study type** | RCT | | |
| **JBI evaluation** | Risk of bias: Low | | |
| **PICO framework** | | | |
| **Population** | Office workers in the civil service of a Dutch city (Enschede). | | |
| | Sample size: 299 | Mean age: 43.74 | Female ratio: 34.8% |
| Intervention(s) | Domain: Physical health & fitness | | |
| | Type: Exercise programs, nutrition and weight management | | |
| | Intervention with seven counselling sessions, each lasting around 20 minutes and was conducted during working hours. Counselling was mainly aimed at promoting physical activity and healthy eating habits, using standardized protocols. | | |
| | Duration: 9 months | | |
| | Setting: In-person | | |
| **Comparator** | Generally written information on lifestyle factors such as physical activity, diet, alcohol, smoking, (work) stress and musculoskeletal complaints but not counselling intervention. | | |
| **Outcome** | Outcome measure(s): Cost-benefit (CBA), cost-effectiveness ratio | | |
| | Secondary outcomes measures included sick leave, physical activity, fitness and musculoskeletal complaints. | | |
| **Results** | The cost-benefit analysis did not reveal any significant cost savings. Although not statistically significant, the difference in the cost of sick days between the intervention and control groups was large. The cost-effectiveness analysis showed that an improvement in energy consumption and cardiorespiratory fitness could be achieved in the intervention group at a higher cost (i.e., €5.2 per additional kilocalorie of energy consumed per day per employee and €235 per beat per minute decrease in submaximal heart rate). | | |

| (74) Rantonen et al. (2018) | | | |
|---|---|---|---|
| **Title** | Effectiveness of three interventions for secondary prevention of low back pain in the occupational health setting – a randomised controlled trial with a natural course control | | |
| **Country scope** | Finland | | |
| **Industry scope** | Basic materials | | |
| **Study type** | Quasi-Experiment | | |
| **JBI evaluation** | Risk of bias: Low | | |
| **PICO framework** | | | |
| **Population** | Employees aged 24 to 56 with non-acute, moderate lumbar spine pain (LS) reporting varying levels of physical and mental stress at work. | | |
| | Sample size: 193 | Mean age: 45 | Female ratio: 34% |
| **Intervention(s)** | Domain: Physical health and fitness | | |
| | Type: Exercise programs | | |
| | The study analyzed three interventions: multidisciplinary rehabilitation in an outpatient hospital department (Rehab), progressive back training in an outpatient clinic (Physio), and self-care counselling provided by the company doctor (Counselling). | | |
| | Duration: 1, 3 and 5 months | | |
| | Setting: In-person | | |
| **Comparator** | The control group received care as usual for lumbar spine (LS) pain, with no study visits and only a follow-up questionnaire after two years. | | |
| **Outcome** | Outcome measure(s): Sick days | | |
| | Primary: Registry data on sick days (48 months), physical impairment, health-related quality of life, and LWS-intensity<br>Secondary: Disability index, pain-related anxiety, and number illness periods | | |
| **Results** | None of the three interventions significantly reduced the number of sick days compared to the control group. The mean differences in sick days were: Rehabilitation: -5 days (95% CI: -34 to 24), p = 0.73; Physiotherapy: 1 day (95% CI: -29 to 31), p = 0.94; Counselling: 11 days (95% CI: -22 to 44), p = 0.51. | | |

| (75) Renaud et al. (2008) | | | |
|---|---|---|---|
| **Title** | Implementation and Outcomes of a Comprehensive Worksite Health Promotion Program | | |
| **Country scope** | Canada | | |
| **Industry scope** | Financials | | |
| **Study type** | Quasi-Experiment | | |
| **JBI evaluation** | Risk of bias: High | | |
| **PICO framework** | | | |
| **Population** | The study involved employees from a single branch of a large financial organization in Quebec, Canada. It evaluated 270 employees who participated in at least three modules of a wellness program, including both the first and last modules. | | |
| | Sample size: 270 | Mean age: 40.7 | Female ratio: N/A |
| **Intervention(s)** | Domain: Physical health and fitness, mental health and stress | | |
| | Type: Exercise programs, nutrition and weight management, health monitoring programs, mindfulness and relaxation | | |
| | The "Take care of your health!" program ran for three years, offering six modules on global health, stress management, heart health, nutrition, and physical activity. Each 1.5–2-hour session was led by health experts, followed by a personalized health profile sent after four weeks. Participants could opt for a follow-up call with a nurse to review their risk profile. | | |
| | Duration: 36 months | | |
| | Setting: In-person, phone-based | | |
| **Comparator** | N/A | | |
| **Outcome** | Outcome measure(s): Sickness absence rate | | |
| | Primary: Self-rated health<br>Secondary: Registry data on sickness absence rate, staff turnover, employee satisfaction, qualitative assessments of the program implementation | | |
| **Results** | During the study, absenteeism in the company dropped by 28%, while staff turnover decreased by 54%. Additionally, participants reported significant improvements in both their overall health status and their experience of stress. | | |

| (76) Robroek et al. (2012) | | | |
|---|---|---|---|
| **Title** | Cost-effectiveness of a long-term Internet-delivered worksite health promotion programme on physical activity and nutrition: a cluster randomized controlled trial | | |
| **Country scope** | Netherlands | | |
| **Industry scope** | Health care, consumer services | | |
| **Study type** | RCT | | |
| **JBI evaluation** | Risk of bias: High | | |
| **PICO framework** | | | |
| **Population** | Employees from six companies in the Netherlands focusing on healthcare, logistics, and public administration. | | |
| | Sample size: 924 | Mean age: 42 | Female ratio: 51% |
| **Intervention(s)** | Domain: Physical health and fitness | | |
| | Type: Exercise programs, nutrition and weight management | | |
| | The program included personalized computer-based counseling, online self-monitoring tools, tailored guidance, and opportunities to consult with health professionals. Participants in the intervention group also received monthly e-mail messages during the first 12 months of the study. | | |
| | Duration: 24 months | | |
| | Setting: In-person, digital (web/app-based) | | |
| **Comparator** | The control group received a standard program that included a health check with personal advice and feedback via a website. Participants also had access to general lifestyle and health information, along with personalized reports based on an online questionnaire. | | |
| **Outcome** | Outcome measure(s): Sickness absence rate | | |
| | Primary: Compliance with physical activity and diet guidelines<br>Secondary: Self-and third-party reported direct and indirect costs from a societal perspective (including sickness absence rate calculation 12 and 24 months) and health outcomes | | |
| **Results** | Sickness absence decreased in both groups after one year, with a greater reduction in the control group. After two years, the intervention group saw a larger decrease. Direct healthcare costs averaged 376 euros per participant, while indirect costs totaled 9,476 euros, with 25% from sickness-related absences and 75% from productivity losses. | | |

**(77) Ryan et al. (2018)**

| | | | |
|---|---|---|---|
| **Title** | Measuring the effectiveness of workplace health program: An Australian example | | |
| **Country scope** | Australia | | |
| **Industry scope** | Health Care | | |
| **Study type** | Pre-post design | | |
| **JBI evaluation** | Risk of bias: High | | |
| **PICO framework** | | | |
| **Population** | Employees of a metropolitan hospital in Melbourne from the operating theatre, kitchen, technology, cleaning, nursing wards, pathology, angiography, intensive care unit, maternity ward, administration and management. | | |
| | Sample size: 1,400 | Mean age: N/A | Female ratio: 86% |
| Intervention(s) | Domain: Physical health and fitness, mental health and stress | | |
| | Type: Exercise programs, mindfulness and relaxation | | |
| | The program, based on a biopsychosocial approach, included daily 6-minute "take-a-break" group sessions at the start of each shift. These sessions focused on health education, relaxation, posture training, stretching, balance, strengthening, and social interaction. Employees unable to return to work received personalized recovery programs from qualified clinicians. | | |
| | Duration: 36 months | | |
| | Setting: In-person | | |
| **Comparator** | Injury management data for the three years prior to the implementation of the program (pre-program) with the three years from the start of the program (post-program) | | |
| **Outcome** | Outcome measure(s): Sick days, insurance claims, cost savings | | |
| | Sick days measured using the "Lost Time Injury Days" (LTI days) - number of days an injured employee was absent from work due to a work injury | | |
| **Results** | LTI days fell from over 1600 in the year before the program to just over 400 and 200 respectively in the two subsequent years. Time to return to work after a compensable injury was reduced (on average) from 80 days to less than 20 days. Duration of recovery and time to return to work after an injury requiring incapacity for work was reduced by 68%. Overall, the number of compensation claims fell by 46%. Total cost of claims made, including medical treatment and sick pay, decreased from $350,000 to $150,000 in the year after the program. | | |

| (78) Saleh et al. (2010) | | | |
|---|---|---|---|
| **Title** | The Effectiveness and Cost-Effectiveness of a Rural Employer-Based Wellness Program | | |
| **Country scope** | United States | | |
| **Industry scope** | Health care, consumer services, financials, not assignable | | |
| **Study type** | Quasi-Experiment | | |
| **JBI evaluation** | Risk of bias: Low | | |
| **PICO framework** | | | |
| **Population** | Employees of six rural employers in New York State. | | |
| | Sample size: 151 | Mean age: 43.3 | Female ratio: 80.3% |
| Intervention(s) | Domain: Physical health and fitness | | |
| | Type: Exercise programs, nutrition and weight management, health monitoring programs | | |
| | The wellness program included two levels of intervention. Group 1 ("Trail Markers") received an annual health risk assessment (HRA) screening for data collection and year-round awareness messages but no further interventions. Group 2 ("Coaching and Referral") received the same awareness messages and HRA screening, along with one-on-one lifestyle coaching and case management for high-risk individuals. | | |
| | Duration: 48 months | | |
| | Setting: N/A | | |
| **Comparator** | No intervention or organized health improvement activities; only collection of HRA data | | |
| **Outcome** | Outcome measure(s): Cost-effectiveness ratio | | |
| | Two cost-effectiveness ratios were calculated: the cost per reduced risk factor and the cost of moving a person to a low-risk classification | | |
| **Results** | The total costs for the respective study groups amounted to 15,072 US dollars for the control group (~$100 per employee); 30,143 US dollars for the trail marker group (~$93.32 per employee); 42,437 US dollars for the coaching and referral group (~$212.19 per employee). The cost per reduced risk factor was $776 for the trail marker group compared to $1,204 for the coaching and referral group. The control group showed no changes in the number of risk factors and an increase in the number and percentage of individuals classified as high-risk, making cost-effectiveness ratios inapplicable for this group. | | |

**(79) Song et al. (2021)**

| | | | |
|---|---|---|---|
| **Title** | Health And Economic Outcomes Up to Three Years After A Workplace Wellness Program: A Randomized Controlled Trial | | |
| **Country scope** | United States | | |
| **Industry scope** | Consumer services | | |
| **Study type** | RCT | | |
| **JBI evaluation** | Risk of bias: Low | | |
| **PICO framework** | | | |
| **Population** | Employees of BJ's Wholesale Club, a large warehouse retailer with approximately 26,000 employees across over 200 locations in the eastern United States. The study specifically targeted middle- and low-income workers. | | |
| | Sample size: 32,974 | Mean age: 38.6 | Female ratio: 45.9% |
| **Intervention(s)** | Domain: Physical health and fitness, mental health and stress | | |
| | Type: Exercise programs, nutrition and weight management., mindfulness and relaxation | | |
| | The multi-component workplace wellness program was developed and delivered by Wellness Workdays, an established wellness provider. It included twelve modules covering key prevention and wellbeing topics such as nutrition, exercise, and stress reduction. | | |
| | Duration: 29 months | | |
| | Setting: N/A | | |
| **Comparator** | No intervention. | | |
| **Outcome** | Outcome measure(s): Sickness absence rate | | |
| | Registry data on work results (including absence days), self-reported health and behaviors, clinical health markers, and healthcare expenditure and utilization | | |
| **Results** | The results indicate that the wellness program had no significant effect on absenteeism. The average absenteeism rate was 2.1% in workplaces with the wellness program and 2.2% in control workplaces. The estimated effect of participation in the wellness program on absenteeism was a decrease of 0.21 percentage points (95% CI: -0.46 to 0.04). While absenteeism was slightly lower in workplaces with the wellness program, the difference was not statistically significant. | | |

| (80) Stansfeld et al. (2015) | | | |
|---|---|---|---|
| **Title** | Pilot study of a cluster randomised trial of a guided e-learning health promotion intervention for managers based on management standards for the improvement of employee well-being and reduction of sickness absence: GEM Study | | |
| **Country scope** | United Kingdom | | |
| **Industry scope** | Health care | | |
| **Study type** | RCT – pilot study | | |
| **JBI evaluation** | Risk of bias: Low | | |
| **PICO framework** | | | |
| **Population** | Staff and managers from four mental health services within an NHS Mental Health Trust in England, excluding employees on long-term sick leave. | | |
| | Sample size: 350 | Mean age: N/A | Female ratio: 76% |
| **Intervention(s)** | Domain: Mental health | | |
| | Type: Mindfulness and relaxation | | |
| | The intervention was a guided e-learning program on work-related stress for managers, based on HSE management standards. It consisted of six online modules completed weekly or biweekly over three months, with additional introductory and follow-up sessions with a study leader, plus phone and email support. | | |
| | Duration: 3 months | | |
| | Setting: In-person | | |
| **Comparator** | The comparison group in the GEM study consisted of managers in a psychiatric service who did not receive any intervention. | | |
| **Outcome** | Outcome measure(s): Sick days | | |
| | Primary: Registry data (< 21 days), well-being<br>Secondary: Self-reported short-term and long-term sickness absence; further information on psychological distress and acquired knowledge | | |
| **Results** | The intervention had no significant effect on sickness-related absences, with a mean difference of 0.6 (95% CI: -1.4 to 2.6). A small positive effect was observed on employee well-being, with Warwick-Edinburgh Mental Well-Being Scale values decreasing from 50.4 to 49.0 in the control group and from 51.0 to 49.9 in the intervention group. | | |

| (81) Taimela et al. (2008) | | | |
|---|---|---|---|
| **Title** | An occupational health intervention programme for workers at high risk for sickness absence. Cost effectiveness analysis based on a randomized controlled trial | | |
| **Country scope** | Finland | | |
| **Industry scope** | Industrials, telecommunication | | |
| **Study type** | RCT | | |
| **JBI evaluation** | Risk of bias: Low | | |
| **PICO framework** | | | |
| **Population** | Employees with a high risk of sickness absence expressing concerns about their future working ability, pain, impairment, sleep, stress, fatigue, or depression. | | |
| | Sample size: 382 | Mean age: 49.5 | Female ratio: 6.5% |
| **Intervention(s)** | Domain: Physical health and fitness | | |
| | Type: Health monitoring programs | | |
| | The intervention group attended a program conducted by nurses and physicians, where they received personal feedback on the results of their assessment. They were also invited to a consultation with the company health service. During the counselling session, an action plan was developed, and if necessary, referrals to specialists or psychologists were made. | | |
| | Duration: N/A | | |
| | Setting: In-person | | |
| **Comparator** | The control group was allowed to contact the occupational doctor or nurse upon request. However, they did not receive feedback on the results of the health survey, nor were they invited to a counselling session. | | |
| **Outcome** | Outcome measure(s): Sick days, cost-effectiveness ratio | | |
| | Self-reported and registry data on sick days (48 months), cost-effectiveness ratio | | |
| **Results** | The intervention resulted in a saving of €17 per day of illness avoided. Cost-effectiveness remained robust across changes in cost parameters, with a probabilistic sensitivity analysis indicating that the intervention was cost-saving and more effective in over 90% of the simulated cases. | | |

**(82) Taimela et al. (2009)**

| | | | |
|---|---|---|---|
| **Title** | Effectiveness of a Targeted Occupational Health Intervention in Workers with High Risk of Sickness Absence: Baseline Characteristics and Adherence as Effect Modifying Factors in a Randomized Controlled Trial | | |
| **Country scope** | Finland | | |
| **Industry scope** | Industrials, Telecommunications | | |
| **Study type** | RCT | | |
| **JBI evaluation** | Risk of bias: Medium | | |
| **PICO framework** | | | |
| **Population** | Employees at high risk of sickness absence, identified through a questionnaire assessing concerns about work ability, pain, sleep, stress, fatigue, or depression. | | |
| | Sample size: 382 | Mean age: 49.5 | Female ratio: 6.5% |
| **Intervention(s)** | Domain: Physical health and fitness | | |
| | Type: Health monitoring programs | | |
| | The intervention group received a program led by nurses and physicians, with personal feedback on assessment results and an invitation for consultation with the company health service. An action plan was created during the counseling session, and referrals to specialists or psychologists were made if needed. | | |
| | Duration: Not specified | | |
| | Setting: In-person | | |
| **Comparator** | The control group could contact the occupational doctor or nurse upon request but did not receive feedback on the health survey results or an invitation for counseling sessions. | | |
| **Outcome** | Outcome measure(s): Sick days | | |
| | Registry data on sick days (12 months) | | |
| **Results** | The analysis revealed an average difference of 11 days between the treatment arms (95% CI: 1–20 days). Subgroup analyses indicated that the intervention was particularly effective for workers who anticipated being unable to continue their current job due to health issues (-74 days; 95% CI: -105 to -43), those with comorbidities (-22.5 days; 95% CI: -35.5 to -9.5), and those experiencing severe physical impairment at work (-17.5 days; 95% CI: -28.5 to -6.5). | | |

| **(83) Thiart et al. (2016)** | | | |
|---|---|---|---|
| **Title** | Internet-Based Cognitive Behavioral Therapy for Insomnia: A Health Economic Evaluation | | |
| **Country scope** | Germany | | |
| **Industry scope** | Not assignable | | |
| **Study type** | RCT | | |
| **JBI evaluation** | Risk of bias: Low | | |
| **PICO framework** | | | |
| **Population** | Teachers with clinically significant insomnia symptoms and work-related brooding. | | |
| | Sample size: 128 | Mean age: 48 | Female ratio: 74.2% |
| Intervention(s) | Domain: Mental health and stress | | |
| | Type: Mental health initiatives/training | | |
| | GET.ON Recovery, an internet-based cognitive behavioral therapy for insomnia (iCBT-I) developed specifically for stressed and sleepless workers. The intervention is mainly based on CBT-I methods, including sleep restriction, stimulus control, sleep hygiene and cognitive interventions. The intervention consists of six one-week modules with e-mail feedback from trained clinical psychologists (e-coaches - maximum 30min per session) for each completed module. | | |
| | Duration: 1.5 months | | |
| | Setting: Digital (web/app-based) | | |
| **Comparator** | Waiting list control group with access to usual treatment | | |
| **Outcome** | Outcome measure(s): ROI, net benefit, benefit-cost-ratio | | |
| | For the cost benefit analysis benefits represent productivity gains from reduced absenteeism and presenteeism, meanwhile costs reflect the €200 per employee expense for the online training. The base for ICER was the number of teachers with treatment response. | | |
| **Results** | Cost benefit analyses: A positive financial gain with a net benefit of €417.63 (CI 95%: −593.03 to 1,488.70), a benefit-cost ratio of 3.09 (CI 95%: −1.97 to 8.44), and a ROI of 208.81% (CI 95%: −296.52 to 744.35) could be observed.<br>Cost effectiveness analyses: Savings of €-1.512 (95 % KI: -4.493 bis 1.128) for each participant with a positive treatment outcome after 6 months. The probability of the intervention being cost-effective with a willingness to pay of zero was 87%, and 95% at WTP of €761. | | |

| (84) Tveito and Eriksen (2008) | | | |
|---|---|---|---|
| **Title** | Integrated health program: a workplace randomized controlled trial | | |
| **Country scope** | Norway | | |
| **Industry scope** | Health Care | | |
| **Study type** | RCT | | |
| **JBI evaluation** | Risk of bias: Medium | | |
| **PICO framework** | | | |
| **Population** | Nursing staff in a care home for the elderly | | |
| | Sample size: 40 | Mean age: N/A | Female ratio: 100% |
| **Intervention(s)** | Domain: Physical health and fitness, mental health and stress | | |
| | Type: Exercise programs, mindfulness and relaxation | | |
| | The integrated health program consists of three main components: physical exercise in the form of one-hour aerobic dance sessions three times per week, 15 hours of information on stress, coping, health, and lifestyle, and a practical workplace examination. | | |
| | Duration: 9 months | | |
| | Setting: In-person | | |
| **Comparator** | The control group participated only in the pre- and post-tests and received the same intervention program as the intervention group only at the end of the study. | | |
| **Outcome** | Outcome measure(s): Sick days | | |
| | Primary: Registry data on sick days (12 months)<br>Secondary: Self-reported psychosocial and health outcomes | | |
| **Results** | The average number of sick days did not differ significantly between groups (intervention: 52.6, control: 54.4; p = 0.945). Across the study population, sick days increased 2.6-fold over three years. The intervention group reported significant subjective improvements in health, physical fitness, muscle pain, stress management, and work-related health. | | |

| (85) Van den Ven et al. (2023) | | | |
|---|---|---|---|
| **Title** | Reach and effectiveness of a worksite health promotion program combining a preventive medical examination with motivational interviewing; a quasi-experimental study among workers in low socioeconomic position | | |
| **Country scope** | Netherlands | | |
| **Industry scope** | Health care, industrials | | |
| **Study type** | Quasi-Experiment | | |
| **JBI evaluation** | Risk of bias: Medium | | |
| **PICO framework** | | | |
| **Population** | Dutch employees in a low socio-economic position, working in a hospital and a manufacturing company, often performing physically demanding work. | | |
| | Sample size: 176 | Mean age: N/A | Female ratio: 84.1% |
| **Intervention(s)** | Domain: Physical health and fitness, mental health and stress | | |
| | Type: Exercise programs, nutrition and weight management, mindfulness and relaxation | | |
| | The workplace health promotion program consisted of a preventive medical check-up (PME) followed by up to 7 sessions of coaching with motivational interviewing (MI) over 6 months. The consultation focused on changes in physical activity, smoking, alcohol consumption, nutrition, and relaxation. | | |
| | Duration: 6 months | | |
| | Setting: In-person, digital (web/app-based) | | |
| **Comparator** | The control group consisted of employees who participated only in the preventive medical check-up (PME) but did not engage in the subsequent motivational interviewing (MI) counseling. | | |
| **Outcome** | Outcome measure(s): Sick days (dichotomized) | | |
| | Primary: Participation<br>Secondary: Self-reported data on sick days (dichotomized), health and behavioral outcomes, ability to work | | |
| **Results** | MI coaching had no significant impact on sick days, but 6.2% more participants had fewer than 10 sick days after 6 months. MI coaching, alongside PME, increased participation in health-promoting activities and smoking cessation among employees in low socioeconomic positions. | | |

| **(86) Van Dongen et al. (2013)** | | | |
|---|---|---|---|
| **Title** | A Cost-effectiveness and Return-on-Investment Analysis of a Worksite Vitality Intervention Among Older Hospital Workers | | |
| **Country scope** | Netherlands | | |
| **Industry scope** | Health care | | |
| **Study type** | RCT | | |
| **JBI evaluation** | Risk of bias: Low | | |
| **PICO framework** | | | |
| **Population** | Older hospital employees (45 years or older) from two Dutch academic hospitals: VU University Medical Centre Amsterdam (VUMC) and Leiden University Medical Centre (LUMC). The inclusion criteria were: (1) working at least 16 hours per week and (2) no risk of developing adverse health effects from physical activity. | | |
| | Sample size: 730 | Mean age: 52.4 | Female ratio: 75.5% |
| Intervention(s) | Domain: Physical health & fitness | | |
| | Type: Exercise programs, nutrition and weight management | | |
| | The Vital@Work intervention included three components: a 24-week Vitality Exercise Program with weekly group yoga and training sessions, 45 minutes of unsupervised physical activity, and three personal counseling sessions with a Personal Vitality Coach. Additionally, free fruit was provided during the group sessions. | | |
| | Duration: 6 months | | |
| | Setting: In-person | | |
| **Comparator** | The control group received written information about a healthy lifestyle in terms of physical activity, diet and relaxation (i.e., care as usual). | | |
| **Outcome** | Outcome measure(s): ROI, net benefit (NB), benefit-cost-ratio (BCR) | | |
| | For NB, BCR, and ROI calculation costs were defined as intervention costs. Meanwhile, benefits were defined as the difference in monetized outcome measures (i.e. absenteeism and presenteeism costs) between the intervention and control groups during follow-up. | | |
| **Results** | Average NB was € -478 (95% CI, -2663 to 1816) per employee indicating a net loss of €478 for the employer at 12 months follow-up. At 12 months follow-up the benefit cost ratio was -2.21 (i.e., returned money per € invested) and the ROI (i.e. the profit percentage per € invested) was -321%. | | |

| (87) Van Dongen et al. (2017) | | | |
|---|---|---|---|
| **Title** | The cost-effectiveness and return-on-investment of a combined social and physical environmental intervention in office employees | | |
| **Country scope** | Netherlands | | |
| **Industry scope** | Financials | | |
| **Study type** | RCT | | |
| **JBI evaluation** | Risk of bias: Low | | |
| **PICO framework** | | | |
| **Population** | Office staff from 19 departments of a financial services provider. Participants on sick leave in the last 4 weeks were excluded from participation | | |
| | Sample size: 412 | Mean age: 41.28 | Female ratio: 39.6% |
| Intervention(s) | Domain: Working atmosphere, physical health and fitness | | |
| | Type: Work climate change, exercise programs | | |
| | The program addressed both social and physical environments, with three 90-minute group motivational interviewing sessions and a refresher, led by team leaders. It also included four "VIP zones" in the workplace, such as coffee and lounge areas. | | |
| | Duration: 3.5 months | | |
| | Setting: Digital (web/app-based), In-person | | |
| **Comparator** | Usual practice (no intervention program) | | |
| **Outcome** | Outcome measure(s): Cost-effectiveness ratio, ROI, net benefit, cost-benefit ratio | | |
| | Primary endpoint the need for recovery. Secondary endpoint general vitality and job satisfaction, ICER (employer and social perspective) and ROI (employer perspective) | | |
| **Results** | All interventions showed negative employer benefits, NBs -3102 (CI 95%: -5897; -93), BCRs -5.7 (CI 95% -11.7;0.8), and ROIs -666 (CI 95% -1266; -20), with a low likelihood of financial return. For ICER (from employer perspective) the combined intervention (social and physical) was associated with an ICER of €-370, physical only with an ICER of €-763 (cost to employer per point improvement) and social only with an ICER of €4256 (costs to employer per point decline in NFR). With a willingness to pay of €300 per point improvement in NFR, the combined intervention became the preferred option. | | |

**(88) Van Holland et al. (2017)**

| | | | |
|---|---|---|---|
| **Title** | Effectiveness and Cost-benefit Evaluation of a Comprehensive Workers' Health Surveillance Program for Sustainable Employability of Meat Processing Workers | | |
| **Country scope** | Netherlands | | |
| **Industry scope** | Consumer staples | | |
| **Study type** | RCT | | |
| **JBI evaluation** | Risk of bias: High | | |
| **PICO framework** | | | |
| **Population** | Meat processing workers performing a minimum of 12 hours of paid labor per week. | | |
| | Sample size: 303 | Mean age: 50.6 | Female ratio: 11.2% |
| **Intervention(s)** | Domain: Physical health and fitness | | |
| | Type: Health monitoring programs | | |
| | The POSE program was a comprehensive employee health monitoring initiative involving screening, counseling, and intervention recommendations. Participants completed an online questionnaire assessing physical and mental health, with results guiding personalized intervention recommendations (e.g., GP or dietician visits). | | |
| | Duration: Not specified | | |
| | Setting: Digital (web/app-based), in-person | | |
| **Comparator** | Regular company healthcare benefits unrelated to the POSE program, as well as all existing programs already running within the company. | | |
| **Outcome** | Outcome measure(s): ROI, net benefit, cost-benefit ratio (CBR) | | |
| | ROI, net benefit, cost-benefit ratio (CBR), sickness absence >9 days | | |
| **Results** | The POSE program resulted in a significant cost increase for employers, with average total benefits per participant at €-775 (95% CI: €-1077 to €-440) and a net benefit of €-975 (95% CI: €-1340 to €-691). The cost-benefit ratio was -3.9 (95% CI: -5.7 to -2.5), while the ROI showed a 487% loss (95% CI: -670% to -345%). | | |

**(89) Viester et al. (2014)**

| | | | |
|---|---|---|---|
| **Title** | The effect of a health promotion intervention for construction workers on work-related outcomes: results from a randomized controlled trial | | |
| **Country scope** | Netherlands | | |
| **Industry scope** | Industrials | | |
| **Study type** | RCT | | |
| **JBI evaluation** | Risk of bias: Low | | |
| **PICO framework** | | | |
| **Population** | Construction workers who participated in a non-compulsory regular health check-up and had not been on sick leave for more than four weeks in the four weeks prior to the check-up. | | |
| | Sample size: 314 | Mean age: 46.6 | Female ratio: N/A |
| **Intervention(s)** | Domain: Physical health and fitness | | |
| | Type: Exercise programs, nutrition and weight management | | |
| | The health promotion program aimed to increase physical activity and improve nutrition. It included lifestyle information, exercise guides, and the "VIP in Construction Toolbox" (health resources, waist tape, pedometer, BMI card, calorie guide, healthy recipes, and a lifestyle quiz). | | |
| | Duration: 6 months | | |
| | Setting: In-person, phone-based | | |
| **Comparator** | The control group received usual care and was only contacted for the baseline and follow-up measurements. | | |
| **Outcome** | Outcome measure(s): Sick days | | |
| | Registry data on work-related outcomes (including sick days [dichotomized as no/short-term (≤7 days) or long-term (>7 days)]), musculoskeletal symptoms, and physical functionality | | |
| **Results** | After the intervention, long-term sickness absence was lower in the intervention group at 6 months but slightly higher at 12 months. However, the differences were not statistically significant. After 12 months, the intervention group had an average of 8.5 sick days (SD: 20.6) compared to 7.5 days in the control group (SD: 16.9). Although the baseline sick days were lower in the control group, the intervention group showed a higher reduction in sick days. | | |

| (90) Von Thiele Schwarz et al. (2012) | | | |
|---|---|---|---|
| **Title** | Effects of Worksite Health Interventions Involving Reduced Work Hours and Physical Exercise on Sickness Absence Costs | | |
| **Country scope** | Sweden | | |
| **Industry scope** | Health care | | |
| **Study type** | Quasi-Experiment | | |
| **JBI evaluation** | Risk of bias: Low | | |
| **PICO framework** | | | |
| **Population** | The study included employees from six public dental health workplaces, selected to have at least 25 employees, being profitable, and receiving consent from management and employees. | | |
| | Sample size: 1,311 | Mean age: 45.2 | Female ratio: 90.7% |
| **Intervention(s)** | Domain: Physical health and fitness, working atmosphere | | |
| | Type: Exercise programs, others | | |
| | The physical exercise (PE) condition required employees to engage in 2.5 hours of medium to high intensity exercise each week, with sessions scheduled on two separate days. The type and duration of each session were tracked and monitored by designated coworkers. In the reduced working hours (RWH) condition, employees worked 37.5 hours per week instead of the usual 40, without mandatory exercise. Part-time workers had a proportional reduction in hours. | | |
| | Duration: 12 months | | |
| | Setting: In-person | | |
| **Comparator** | Employees at workplaces that have not undergone either of the two interventions (PE or RWH) and continued to work 40 hours a week. | | |
| **Outcome** | Outcome measure(s): Sick days | | |
| | Primary: Costs of sick days<br>Secondary: Registry data on sick days (12 months), prolonged illness, organizational procedures, and productivity | | |
| **Results** | Sick days decreased in all groups, with the largest reduction in the reference group (-15.9%), followed by the PE group (-11.4%) and the RWH group (-4.9%). The PE group also saw a significant cost reduction (-22.2%), while the reference group experienced a cost increase (+10.2%). The PE intervention proved cost-effective, reducing both direct and indirect costs. | | |

| **(91) Vonderlin et al. (2023)** | | | |
|---|---|---|---|
| **Title** | Improving Health and Reducing Absence Days at Work: Effects of a Mindfulness- and Skill-Based Leadership Intervention on Supervisor and Employee Sick Days | | |
| **Country scope** | Germany | | |
| **Industry scope** | Health care | | |
| **Study type** | Quasi-Experiment | | |
| **JBI evaluation** | Risk of bias: Low | | |
| **PICO framework** | | | |
| **Population** | Managers and their employees from 12 participating companies. | | |
| | Sample size: 1,830 | Mean age: 43.5 | Female ratio: 78% |
| **Intervention(s)** | Domain: Physical health and fitness, mental health and stress | | |
| | Type: Health monitoring programs, mindfulness and relaxation | | |
| | The three-day mindfulness and skills-based leadership program (MBP) aims to promote health-related mindfulness and behavior among managers. It included three full-day courses and two three-hour refresher sessions on self-care, employee care, and handling stressed employees, led by experienced health coaches and psychologists with preventive health intervention experience. | | |
| | Duration: 6 months | | |
| | Setting: In-person | | |
| **Comparator** | No intervention. | | |
| **Outcome** | Outcome measure(s): Sick days | | |
| | Registry data on sick days (24 months) | | |
| **Results** | Managers in the MBP group had significantly fewer non-specific sick days than the control group in the two years following the intervention. On average, managers in the intervention group had 13.9 sick days (adjusted mean), while those in the control group had 32.9 sick days (adjusted mean, $\chi2[1] = 4.38$, $p = 0.036$, $d = 0.47$), with the effect primarily driven by an increase in sick days in the control group. At the employee level, no significant differences were found between the experimental and control groups. | | |

**(92) Wijnen et al. (2019)**

| | | | |
|---|---|---|---|
| **Title** | Implementing interventions to reduce work-related stress among health-care workers: an investment appraisal from the employer's perspective | | |
| **Country scope** | Netherlands | | |
| **Industry scope** | Health care | | |
| **Study type** | Cohort study | | |
| **JBI evaluation** | Risk of bias: Low | | |
| **PICO framework** | | | |
| **Population** | Healthcare workers, mainly carers. | | |
| | Sample size: 303 | Mean age: 44.45 | Female ratio: 96.4% |
| **Intervention(s)** | Domain: Mental health and stress | | |
| | Type: Mindfulness and relaxation, mental health policies | | |
| | Stress-Prevention@Work is a digital platform helping healthcare teams assess stress needs, select interventions like communication guidelines, and evaluate their effectiveness, focusing on both organizational and individual solutions. | | |
| | Duration: 12 months | | |
| | Setting: Digital (web/app-based) | | |
| **Comparator** | Waiting list control group receiving access to the intervention after 12 months. | | |
| **Outcome** | Outcome measure(s): ROI, net benefit, cost-benefit analysis | | |
| | Self-reported data on ROI, net benefit, and productivity losses (12 months, including absence days) | | |
| **Results** | The Stress-Prevention@Work intervention showed a net benefit of €2981 per employee per year, with an ROI of 59.6 from a €50 investment. There was a 96.7% chance of cost recovery within a year and an 88.2% likelihood of achieving €1000 in net benefit. The positive ROI was driven by low costs, reduced productivity losses, and early impact. | | |

**(93) Zetterberg et al. (2022)**

| | |
|---|---|
| **Title** | Preventing Pain and Stress-Related Ill-Health in Employees: A 6-Months Follow-Up of a Psychosocial Program in a Cluster Randomized Controlled Trial |
| **Country scope** | Sweden |
| **Industry scope** | Health care, industrials and consumer services |
| **Study type** | RCT |
| **JBI evaluation** | Risk of bias: Low |
| **PICO framework** | |
| **Population** | Employees who reported pain and/or stress-related illnesses, with the majority of participants being women working in public organizations such as healthcare, schools, and administrative departments.<br>Sample size: 147 / Mean age: 43.3 / Female ratio: 94.6% |
| **Intervention(s)** | Domain: Physical health and fitness, mental health and stress<br>Type: Health monitoring programs, mental health initiatives/training<br>The "Effective Communication within the Organization" (ECO) program aimed to address workplace pain and stress. It included 2-3 hybrid group sessions (live or online) led by psychologists, featuring skill training, case studies, role-playing, assignments, and reflection.<br>Duration: 6 months<br>Setting: In-person, digital (web/app-based) |
| **Comparator** | The control group received a psychoeducational intervention consisting of two one-hour lectures on risk factors and self-management of pain and/or stress-related illnesses, along with information brochures on pain and stress. |
| **Outcome** | Outcome measure(s): Sick days<br>Primary: Registry data on sick days (6 months)<br>Secondary: Self-reported health and well-being |
| **Results** | There was no statistically significant difference in the number of sick days between the intervention group (ECO) and the control group (PE) during the follow-up period. The intervention group reported 8.93 sick days (SD = 27.2), while the control group reported 9.42 sick days (SD = 34.86) during the 6-month follow-up. Self-reported sick days in the last year also did not differ between the two groups after 6 months. |

**Table S5. Summary statistics of papers included in the review (n = 68)**

| | | | | | | | |
|---|---|---|---|---|---|---|---|
| Statistics on study design | | | | | | | |
| **RCT ratio**<br>[#/% of studies] | 36 (52.9%) | **Total sample size**<br>[across incl. papers] | ~144k | **Mean age**<br>[in years] | 40.26 | **Avg. female ratio**<br>[in %] | 45.52 |
| Statistics on occupational health interventions | | | | | | | |
| **Country scope**<br>[#/% of studies] | | **Industry scope**<br>[#/% of studies] | | **Delivery format**<br>[#/% of studies] | | **Target level**<br>[#/% of studies] | |
| Netherlands | 18 (26.5%) | Health care | 21 (30.9%) | In-person only | 30 (44.1%) | Individual level | 63 (92.6%) |
| USA | 15 (22.1%) | Industrials | 15 (22.1%) | Phone only | 2 (2.9%) | Leadership | 13 (19.1%) |
| Germany | 6 (8.8%) | Consumer services | 9 (13.2%) | Digital only | 6 (8.8%) | Organizational | 9 (13.2%) |
| Sweden | 6 (8.8%) | Financials | 5 (7.4%) | In-person/phone | 5 (7.4%) | Team | 5 (7.4%) |
| Denmark | 5 (7.4%) | Technology | 3 (4.4%) | In-person/digital | 11 (16.2%) | | |
| Japan | 3 (4.4%) | Telecommunication | 2 (2.9%) | Phone/digital | 1 (1.5%) | | |
| U.K. | 3 (4.4%) | Basic materials | 1 (1.5%) | | | | |
| Finland | 3 (4.4%) | Consumer staples | 1 (1.5%) | | | | |
| Australia | 2 (2.9%) | Not assignable | 9 (1.5%) | | | | |
| Canada | 2 (2.9%) | No reference | 15 (22.1%) | | | | |
| Norway | 2 (2.9%) | | | | | | |
| Turkey | 1 (1.5%) | | | | | | |
| Switzerland | 1 (1.5%) | | | | | | |
| No reference | 1 (1.5%) | | | | | | |

**Table S6. Overview of JBI critical appraisal results**

Green: Low risk of bias (0.70 – 1.0); orange = moderate risk of bias (0.50 – 0.70), red = high risk of bias (0 – 0.50)

| | | Domains for assessment of risk of bias for randomized controlled trials (2023) | | | | | | | | | | | | | JBI rating (-5 & -6) |
|---|---|---|---|---|---|---|---|---|---|---|---|---|---|---|---|
| Authors | Year | 1 | 2 | 3 | 4 | (5) | (6) | 7 | 8 | 9 | 10 | 11 | 12 | 13 | |
| Karlsson et al. | 2024 | Yes | No | Yes | Yes | No | No | Unclear | Yes | Yes | Yes | Yes | Yes | Yes | 0.82 |
| Keus van de Poll et al. | 2020 | Yes | Yes | No | Yes | No | Unclear | Unclear | Yes | Yes | Yes | Yes | Yes | Yes | 0.82 |
| Duijts et al. | 2008 | Yes | No | Yes | No | No | Yes | Unclear | Yes | No | No | Yes | Yes | Yes | 0.55 |
| Taimela et al. | 2009 | Yes | Unclear | Yes | No | Unclear | Yes | Unclear | Yes | Yes | No | Yes | Yes | Yes | 0.64 |
| Arends et al. | 2013 | Yes | Yes | Unclear | Yes | No | Unclear | Unclear | Yes | Yes | No | Yes | Yes | Yes | 0.73 |
| Keus van de Poll et al. | 2020 | Yes | Yes | Yes | Yes | No | Unclear | Unclear | Yes | Yes | Yes | Unclear | Yes | Yes | 0.82 |
| Stansfeld et al. | 2015 | Yes | Yes | Yes | Yes | No | Unclear | Unclear | Yes | Yes | Yes | Unclear | Yes | Yes | 0.82 |
| Framke et al. | 2016 | Yes | Yes | Yes | Unclear | No | Unclear | Yes | Yes | Yes | Yes | Yes | Yes | Yes | 0.91 |
| Klasen et al. (RCT 2) | 2021 | Yes | Unclear | Yes | No | Unclear | Unclear | Unclear | Yes | Yes | Yes | Yes | Yes | Yes | 0.73 |
| Van Holland et al. | 2017 | Yes | Unclear | No | No | Unclear | Unclear | Unclear | Yes | Yes | No | Unclear | Yes | Unclear | 0.36 |
| Krampen | 2010 | Yes | Unclear | Yes | Unclear | Unclear | Unclear | Unclear | Yes | Yes | Unclear | Unclear | No | Yes | 0.45 |
| Tveito and Eriksen | 2008 | Yes | Yes | Yes | Unclear | Unclear | Unclear | Unclear | Yes | Yes | No | Unclear | No | Yes | 0.55 |
| De Boer et al. | 2004 | Yes | Unclear | Yes | Unclear | Unclear | Unclear | Unclear | Yes | Yes | Yes | Yes | Yes | Yes | 0.73 |
| Hengel et al. | 2013 | Yes | Yes | Yes | Unclear | Unclear | Unclear | Unclear | Yes | Yes | No | Yes | Yes | Yes | 0.73 |
| Zetterberg et al. | 2022 | Yes | Yes | No | Unclear | Unclear | Yes | Unclear | Yes | Yes | Yes | Yes | Yes | Yes | 0.73 |
| Milani et al. | 2009 | Yes | Unclear | Yes | Unclear | Unclear | Unclear | Unclear | Yes | Yes | Yes | Unclear | No | Yes | 0.55 |
| Hengel et al. | 2014 | Yes | Yes | No | No | No | Unclear | Unclear | Yes | Yes | Yes | Yes | Yes | Yes | 0.73 |
| Viester et al. | 2014 | Yes | Yes | Yes | No | Unclear | Unclear | Yes | Yes | Yes | Yes | Yes | Yes | Yes | 0.91 |
| Taimela et al. | 2007 | Yes | Unclear | Yes | No | Unclear | Unclear | Unclear | Yes | Yes | Yes | Yes | Yes | Yes | 0.73 |
| Ebert et al. | 2018 | Yes | Yes | Yes | Unclear | No | Unclear | Unclear | Yes | Yes | Yes | Yes | Yes | Yes | 0.82 |
| Lerner et al. | 2020 | Yes | Yes | Yes | Unclear | Unclear | Yes | Yes | Yes | Yes | Yes | Yes | Yes | Yes | 0.91 |
| Song et al. | 2021 | Yes | Unclear | Yes | No | Unclear | Unclear | Unclear | Yes | Yes | Yes | Yes | Yes | Yes | 0.73 |
| Robroek et al. | 2012 | Yes | Yes | No | Yes | Unclear | No | Unclear | Yes | No | No | Unclear | No | Yes | 0.45 |
| Meenan et al. | 2010 | Yes | Unclear | Unclear | No | Unclear | Unclear | Unclear | Yes | No | Yes | Unclear | No | Yes | 0.36 |
| Freund et al. | 2024 | Yes | Yes | Yes | No | N/A | Unclear | Unclear | Yes | Yes | Yes | Yes | Yes | Yes | 0.82 |
| Geraedts et al. | 2015 | Yes | Yes | Unclear | No | No | Unclear | Unclear | Yes | Yes | No | Yes | Yes | Yes | 0.64 |
| Noben et al. | 2015 | Yes | Unclear | Yes | Unclear | Unclear | Unclear | Unclear | Yes | Yes | Yes | Yes | Yes | Yes | 0.73 |
| Brox & Frøystein | 2005 | Yes | Yes | No | Unclear | Unclear | Unclear | Yes | Yes | Yes | No | Yes | No | Yes | 0.64 |
| Blangsted et al. | 2008 | Yes | Unclear | Yes | Unclear | Unclear | Unclear | Yes | Yes | No | No | Yes | No | Yes | 0.55 |
| Jorgensen et al. | 2011 | Yes | Yes | Yes | No | Unclear | Unclear | Unclear | Yes | Yes | Yes | Yes | Yes | Yes | 0.82 |
| Herman et al. | 2008 | Yes | No | No | Unclear | No | Yes | Unclear | Yes | Yes | Yes | Yes | Yes | Yes | 0.64 |
| Van Dongen et al. | 2013 | Yes | Yes | Yes | Unclear | Unclear | Unclear | Unclear | Yes | Yes | Yes | Yes | Yes | Yes | 0.82 |
| Thiart et al. | 2016 | Yes | Yes | Yes | Unclear | Unclear | Unclear | Unclear | Yes | Yes | Yes | Yes | Yes | Yes | 0.82 |

| Authors | Year | Domains for assessment of risk of bias for randomized controlled trials (2023) | | | | | | | | | | | | | JBI rating (-5 & -6) |
|---|---|---|---|---|---|---|---|---|---|---|---|---|---|---|---|
| | | 1 | 2 | 3 | 4 | (5) | (6) | 7 | 8 | 9 | 10 | 11 | 12 | 13 | |
| Van Dongen et al. | 2017 | Yes | Unclear | Yes | Unclear | Unclear | Unclear | Unclear | Yes | Yes | Yes | Yes | Yes | Yes | 0.73 |
| Groeneveld et al. | 2011 | Yes | Yes | Yes | No | No | Unclear | Yes | Yes | No | Yes | Yes | Yes | Yes | 0.82 |
| Proper et al. | 2004 | Yes | Yes | Yes | Unclear | No | Unclear | Unclear | Yes | Yes | Yes | Unclear | Yes | Yes | 0.73 |

| Authors | Year | Domains for assessment of risk of bias for quasi-experimental studies (2024) | | | | | | | | | JBI Rating (-4) |
|---|---|---|---|---|---|---|---|---|---|---|---|
| | | 1 | 2 | 3 | (4) | 5 | 6 | 7 | 8 | 9 | |
| Linden et al. | 2014 | Yes | Yes | Unclear | Unclear | Yes | Yes | Unclear | Unclear | No | 0.50 |
| Akerstrom et al. | 2021 | Yes | Yes | Unclear | Unclear | Yes | Yes | No | Unclear | Yes | 0.63 |
| Kobayashi et al. | 2008 | Yes | Yes | No | Unclear | Yes | Unclear | No | Yes | Yes | 0.63 |
| Vonderlin et al. | 2023 | Yes | Yes | No | Unclear | Yes | Yes | Yes | Yes | Yes | 0.88 |
| Blake et al. | 2013 | Yes | No | N/A | N/A | Yes | N/A | No | N/A | Yes | 0.38 |
| Van den Ven et al. | 2023 | Yes | Yes | Unclear | Unclear | Yes | Unclear | No | No | Yes | 0.50 |
| Makrides et al. | 2011 | Yes | No | N/A | N/A | Yes | N/A | Yes | N/A | Yes | 0.50 |
| Von Thiele Schwarz et al. | 2012 | Yes | Yes | Yes | Unclear | Yes | Yes | Yes | No | No | 0.75 |
| Rantonen et al. | 2018 | Yes | Yes | Yes | Unclear | Yes | Yes | Yes | Yes | Yes | 1.00 |
| Hendriksen et al. | 2016 | Yes | No | N/A | N/A | Yes | N/A | Yes | N/A | Yes | 0.50 |
| Losina et al. | 2017 | Yes | No | N/A | N/A | Yes | N/A | Yes | N/A | Yes | 0.50 |
| Gregson et al. | 2023 | Yes | No | N/A | N/A | Yes | N/A | Yes | N/A | Yes | 0.50 |
| Renaud et al. | 2008 | Yes | No | N/A | N/A | Yes | N/A | Yes | N/A | No | 0.38 |
| Ornek et al. | 2020 | Yes | Yes | Yes | Unclear | Yes | No | Yes | Yes | Yes | 0.88 |
| Iijima et al. | 2013 | Yes | No | N/A | N/A | No | N/A | No | N/A | No | 0.13 |
| Bondar et al. | 2022 | Yes | No | N/A | N/A | Yes | N/A | Yes | N/A | Yes | 0.50 |
| Goetzel et al. | 2014 | Yes | No | N/A | N/A | Yes | N/A | No | N/A | No | 0.25 |
| Hughes et al. | 2007 | Yes | No | N/A | N/A | Yes | N/A | Yes | N/A | Unclear | 0.38 |
| Baker et al. | 2008 | Yes | No | N/A | N/A | Yes | N/A | No | N/A | No | 0.25 |
| Norwitz et al. | 2022 | Yes | No | N/A | N/A | Yes | N/A | No | N/A | Yes | 0.38 |
| Gubler et al. | 2017 | Yes | Yes | Yes | No | Yes | Yes | No | Yes | Yes | 0.88 |
| Braun et al. | 2014 | Yes | No | N/A | N/A | Yes | N/A | No | N/A | No | 0.25 |
| Larsen et al. | 2019 | Yes | Yes | Yes | Unclear | Yes | Yes | Yes | Yes | Yes | 1.00 |
| Saleh et al. | 2010 | Yes | Yes | No | Unclear | Yes | Yes | Yes | Yes | No | 0.75 |
| Ikegami et al. | 2010 | Yes | No | N/A | N/A | Yes | N/A | Yes | N/A | Yes | 0.50 |
| Ryan et al. | 2018 | Yes | No | N/A | N/A | Yes | N/A | Yes | N/A | No | 0.38 |
| Musich et al. | 2015 | Yes | Yes | Yes | Unclear | Yes | Yes | Yes | No | Yes | 0.88 |

| Authors | Year | Domains for assessment of risk of bias for cohort studies (2024) | | | | | | | | | | | JBI Rating |
|---|---|---|---|---|---|---|---|---|---|---|---|---|---|
| | | 1 | 2 | 3 | 4 | 5 | 6 | 7 | 8 | 9 | 10 | 11 | |
| Dement et al. | 2015 | Yes | Yes | Yes | Yes | Yes | Yes | Yes | Yes | No | Yes | Yes | 0.91 |
| Kapinos et al. | 2015 | Yes | Yes | Yes | Yes | Yes | Yes | Yes | Unclear | No | No | Yes | 0.73 |
| Wijnen et al. | 2019 | Yes | Yes | Yes | Yes | Yes | Yes | Yes | Yes | No | Yes | Yes | 0.91 |
| Jenny et al. | 2011 | Unclear | Unclear | No | Yes | Yes | Unclear | Yes | Yes | No | No | No | 0.36 |

| Authors | Year | Domains for economic evaluations (2020) | | | | | | | | | | | JBI Rating |
|---|---|---|---|---|---|---|---|---|---|---|---|---|---|
| | | 1 | 2 | 3 | 4 | 5 | 6 | 7 | 8 | 9 | 10 | 11 | |
| Keus van de Poll et al. | 2020 | Yes | Yes | Yes | Yes | No | Yes | Yes | Yes | Yes | Yes | Yes | 0.91 |
| Van Holland et al. | 2017 | Yes | Yes | Yes | No | Yes | Yes | No | Yes | Yes | Yes | No | 0.82 |
| Milani et al. | 2009 | Yes | Yes | Yes | Yes | Yes | Yes | Yes | Yes | No | Yes | No | 0.82 |
| Hengel et al. | 2014 | Yes | Yes | Yes | No | Yes | Yes | Yes | Yes | Yes | Yes | Yes | 0.91 |
| Dement et al. | 2015 | Yes | Yes | Yes | Yes | Yes | Yes | No | Yes | Yes | Yes | Yes | 0.91 |
| Von Thiele Schwarz et al. | 2012 | Yes | Yes | Yes | Yes | Yes | Yes | Yes | Yes | No | Yes | No | 0.82 |
| Taimela et al. | 2007 | Yes | Yes | Yes | Yes | Yes | Yes | No | Yes | Yes | Yes | Yes | 0.91 |
| Kapinos et al. | 2015 | Yes | Yes | Yes | Yes | Yes | Yes | Yes | Yes | No | Yes | Yes | 0.91 |
| Ebert et al. | 2018 | Yes | Yes | Yes | Yes | Yes | Yes | Yes | Yes | Yes | Yes | Yes | 1.00 |
| Wijnen et al. | 2019 | Yes | Yes | Yes | Yes | Yes | Yes | Yes | Yes | Yes | Yes | Yes | 1.00 |
| Iijima et al. | 2013 | Yes | No | Yes | No | No | Yes | Yes | Yes | No | Yes | No | 0.55 |
| Elson et al. | 2019 | Yes | No | Yes | Yes | No | Yes | Yes | No | Yes | Yes | No | 0.64 |
| Bondar et al. | 2022 | Yes | No | Yes | Yes | No | Yes | No | Yes | Yes | Yes | No | 0.64 |
| Goetzel et al. | 2014 | Yes | No | Yes | Yes | No | Yes | Yes | Yes | No | Yes | No | 0.64 |
| Lerner et al. | 2020 | Yes | Yes | Yes | Yes | Yes | Yes | Yes | Yes | Yes | Yes | No | 0.91 |
| Baker et al. | 2008 | Yes | Yes | Yes | No | Yes | Yes | Yes | Yes | No | No | Yes | 0.73 |
| Meenan et al. | 2010 | Yes | Yes | Yes | Yes | No | Yes | Yes | Yes | Yes | No | No | 0.73 |
| Norwitz et al. | 2022 | Yes | No | No | Yes | No | Yes | Yes | No | No | Yes | No | 0.45 |
| Gubler et al. | 2017 | Yes | Yes | Yes | Yes | Yes | Yes | No | Yes | No | Yes | No | 0.73 |
| Braun et al. | 2014 | Yes | No | No | No | No | No | No | No | No | No | No | 0.09 |
| Saleh et al. | 2010 | Yes | Yes | Yes | Yes | Yes | No | No | Yes | Yes | Yes | No | 0.73 |
| Ryan et al. | 2018 | Yes | No | Yes | No | Yes | Yes | No | No | No | Yes | No | 0.45 |
| Freund et al. | 2024 | Yes | Yes | Yes | Yes | Yes | Yes | Yes | Yes | Yes | Yes | Yes | 1.00 |
| Geraedts et al. | 2015 | Yes | Yes | Yes | Yes | Yes | Yes | Yes | Yes | Yes | Yes | Yes | 1.00 |

| Authors | Year | Domains for economic evaluations (2020) | | | | | | | | | | | JBI Rating |
|---|---|---|---|---|---|---|---|---|---|---|---|---|---|
| | | 1 | 2 | 3 | 4 | 5 | 6 | 7 | 8 | 9 | 10 | 11 | |
| Noben et al. | 2015 | Yes | Yes | Yes | Yes | Yes | Yes | Yes | Yes | Yes | Yes | No | 0.91 |
| Herman et al. | 2008 | Yes | Yes | Yes | Yes | Yes | Yes | Yes | Yes | Yes | Yes | No | 0.91 |
| Van Dongen et al. | 2013 | Yes | Yes | Yes | No | Yes | Yes | Yes | Yes | Yes | Yes | Yes | 0.91 |
| Thiart et al. | 2016 | Yes | Yes | Yes | Yes | Yes | Yes | Yes | Yes | Yes | Yes | Yes | 1.00 |
| Van Dongen et al. | 2017 | Yes | Yes | Yes | Yes | Yes | Yes | Yes | Yes | Yes | Yes | No | 0.91 |
| Groeneveld et al. | 2011 | Yes | Yes | Yes | Yes | No | Yes | Yes | Yes | Yes | No | Yes | 0.82 |
| Proper et al. | 2004 | Yes | Yes | Yes | Yes | Yes | Yes | Yes | Yes | Yes | Yes | Yes | 1.00 |
| Musich et al. | 2015 | Yes | No | Yes | Yes | Yes | No | Yes | Yes | No | Yes | No | 0.64 |
| Jenny et al. | 2011 | Yes | Yes | Yes | Yes | Yes | No | No | Yes | No | Yes | No | 0.64 |

**Table S7. Leave-one-out analysis for low risk-of-bias studies (JBI > 0.7)**

| Omitted study | MD sick days | 95% CI | p-value | $I^2$ |
|---|---|---|---|---|
| Stansfeld et al. (2015) | -0.72 | [-3.58, 2.13] | 0.62 | 0% |
| Framke et al. (2016) | -0.18 | [-2.80, 2.43] | 0.89 | 0% |
| Klasen et al. (2021) | 0.01 | [-2.54, 2.56] | 0.99 | 0% |
| De Boer et al. (2004) | -0.18 | [-2.80, 2.43] | 0.89 | 0% |
| Zetterberg et al. (2022) | -0.26 | [-3.04, 2.52] | 0.85 | 0% |
| Vonderlin et al. – Supervisor (2023) | 0.24 | [-1.40, 1.88] | 0.77 | 0% |
| Vonderlin et al. – Employee (2023) | -0.46 | [-1.44, 0.51] | 0.76 | 0% |
| Hengel et al. (2014) | -0.18 | [-2.80, 2.43] | 0.89 | 0% |
| Viester et al. (2014) | -0.43 | [-3.39, 2.51] | 0.77 | 0% |
| Rantonen et al. – Rehab (2018) | -0.19 | [-2.99, 2.60] | 0.89 | 0% |
| Rantonen et al. – Physio (2018) | -0.24 | [-3.03, 2.55] | 0.86 | 0% |
| Rantonen et al. – Advice (2018) | -0.28 | [-2.99, 2.43] | 0.84 | 0% |
| Larsen et al. (2019) | -0.15 | [-3.58, 3.08] | 0.89 | 0% |
| **Random-effects model (all studies)** | **-0.18** | **[-2.80, 2.43]** | **0.89** | **0%** |

| Omitted study | ROI | 95% CI | p-value | $I^2$ |
|---|---|---|---|---|
| Keus van de Poll et al. (2020) | 1.92 | [-0.34, 4.17] | 0.096 | 33.4% |
| Hengel et al. (2014) | 1.20 | [-0.82, 3.22] | 0.244 | 90.2% |
| Dement et al. (2015) | 1.92 | [-0.34, 4.17] | 0.096 | 33.4% |
| Ebert et al. (2018) | 2.74 | [-0.50, 5.97] | 0.097 | 32.6% |
| Wijnen et al. (2019) | 1.82 | [-0.39, 4.03] | 0.095 | 33.8% |
| Lerner et al. (2020) | 1.97 | [-0.33, 4.27] | 0.095 | 33.4% |
| Gubler et al. (2017) | 1.92 | [-0.34, 4.17] | 0.095 | 33.4% |
| Freund et al. (2024) | 2.38 | [-0.58, 5.34] | 0.115 | 41.2% |
| Noben et al. (2015) | 1.15 | [-0.81, 3.10] | 0.255 | 0.0% |
| Van Dongen et al. (2013) | 2.12 | [-0.29, 4.53] | 0.085 | 49.7% |
| Thiart et al. (2016) | 2.05 | [-0.88, 5.29] | 0.067 | 41.6% |
| Van Dongen et al. – Social (2017) | 2.62 | [-0.10, 5.34] | 0.059 | 33.3% |
| Van Dongen et al. – Physical (2017) | 1.96 | [-0.31, 4.25] | 0.092 | 48.1% |
| **Random-effects model (all studies)** | **1.92** | **[-0.34, 4.17]** | **0.096** | **33.4%** |

**Figure S1. Search results by intervention type and outcome measure**

Figure 1 illustrates the search logic. Numbers that are connected with Boolean logical operators reflect the corresponding search strings in Illustration 1 of the Supplement Material.

| | Economics:<br>#1 AND<br>(#2 OR #3 OR #4 OR #5)<br>*Hits: 1,795* | | Sickness absence:<br>#1 AND<br>(#2 OR #3 OR #4 OR #5)<br>*Hits: 816* | |
|---|---|---|---|---|
| Intervention type | PubMed | WoS | PubMed | WoS |
| Mental Health<br>#1 AND #2<br>AND (#6 OR #7)<br>Hits: 744 | #1 AND #2<br>AND #6<br>Hits: 197 | #1 AND #2<br>AND #6<br>Hits: 270 | #1 AND #2 AND<br>#7<br>Hits: 111 | #1 AND #2 AND<br>#7<br>Hits: 166 |
| Physical activity<br>#1 AND #3<br>AND (#6 OR #7)<br>Hits: 856 | #1 AND #3<br>AND #6<br>Hits: 267 | #1 AND #3<br>AND #6<br>Hits: 327 | #1 AND #3 AND<br>#7<br>Hits: 104 | #1 AND #3 AND<br>#7<br>Hits: 158 |
| Nutrition<br>#1 AND #4<br>AND (#6 OR #7)<br>Hits: 259 | #1 AND #4<br>AND #6<br>Hits: 72 | #1 AND #4<br>AND #6<br>Hits: 121 | #1 AND #4 AND<br>#7<br>Hits: 21 | #1 AND #4 AND<br>#7<br>Hits: 45 |
| Working climate<br>#1 AND #5<br>AND (#6 OR #7)<br>Hits: 752 | #1 AND #5<br>AND #6<br>Hits: 250 | #1 AND #5<br>AND #6<br>Hits: 291 | #1 AND #5 AND<br>#7<br>Hits: 80 | #1 AND #5 AND<br>#7<br>Hits: 131 |

**Figure S2. Evolution of OHI delivery formats for studies included in the systematic review**

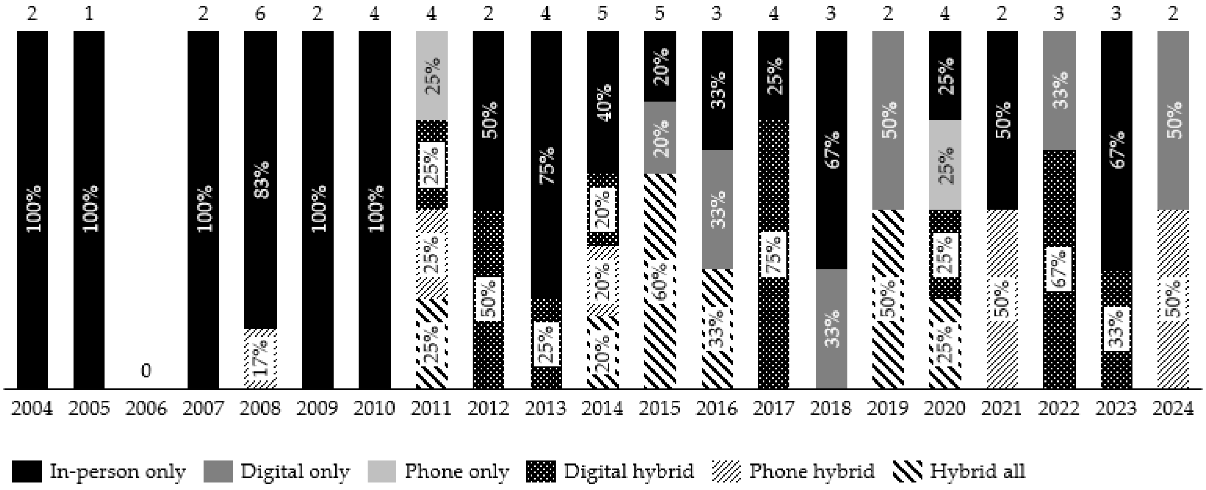


Note: Numbers on top of the bar chart indicate the number of studies included in this systematic review at this point in time.

**Figure S3. Overview of study results and risk of bias for selected studies clustered by industry**

Study numbers correspond to the reference number and numbering of Table 4 of the Supplement Material, with some studies appearing multiple times as they evaluate various interventions and outcomes. Shading pattern reflects the JBI rating: fully shaded for low (JBI > 0.7), dotted for moderate (0.5–0.7), and striped for high risk of bias (< 0.5). Colors indicate effect direction: black for benefits, dark gray for no effect, and light grey for negative outcomes. Non-significant results are classified as no effect. Stars indicate statistical significance levels reported for outcome variable in respective study. Crosses represent studies without reported significance levels. 1) Return on investment. 2) Cost-benefit analysis. 3) Cost-benefit ratio. 4) Cost-effectiveness ratio. 5) E.g., Reduced work hours

## I. Studies conducted in the healthcare sector

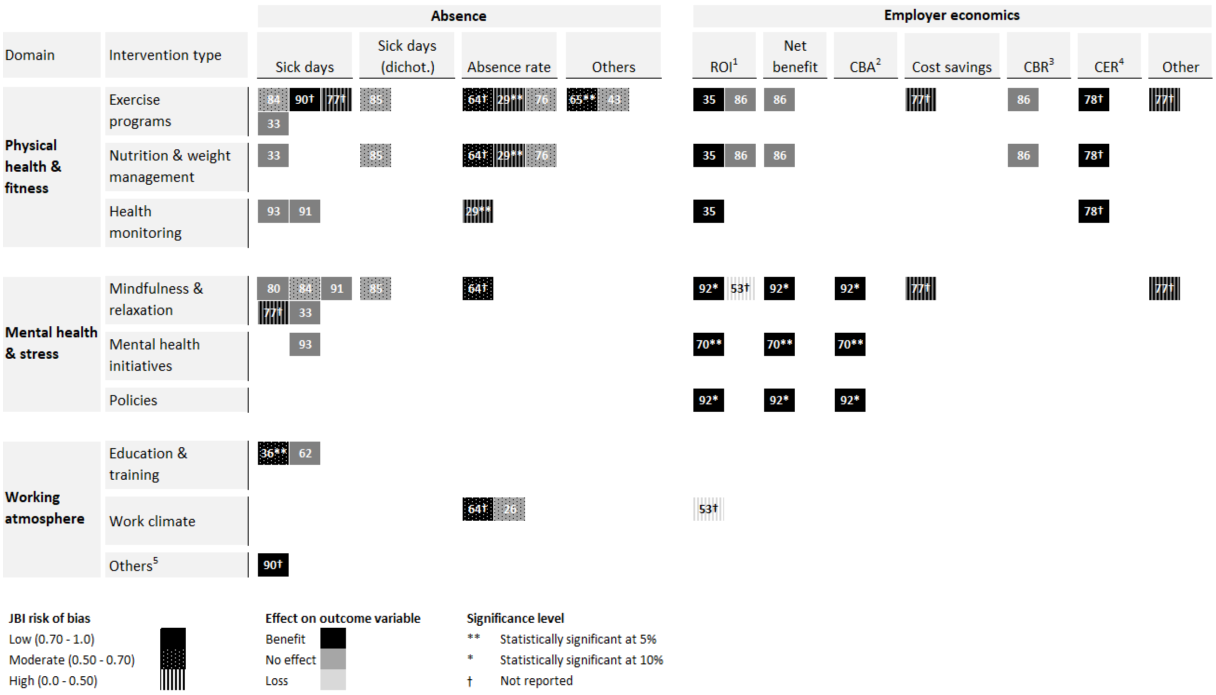

## II. Studies conducted in the industrial sector

| Domain | Intervention type | Absence: Sick days | Absence: Sick days (dichot.) | Absence: Absence rate | Employer economics: ROI[1] | Employer economics: Net benefit | Employer economics: Cost savings | Employer economics: CBR[3] | Employer economics: CER[4] | Employer economics: Other |
|---|---|---|---|---|---|---|---|---|---|---|
| **Physical health & fitness** | Exercise programs | 61** 48† 89 54 | 47 85 | 64† | 48** 49† | | 44 | | | |
| | Nutrition & weight management | 61** 89 | 85 | 64† | 49† | | 44 | | | |
| | Health monitoring | 81** 93 82* | | | 45† | | | | 82* | |
| **Mental health & stress** | Mindfulness & relaxation | 61** 48† | 47 85 | 64† | 48** 41 49† 53† | 41 | | 41 | 41 | 41 |
| | Mental health initiatives | 93 | | | 41 | 41 | | 41 | 41 | 41 |
| | Policies | | | | | | | | | |
| **Working atmosphere** | Education & training | | | | | | | | | |
| | Work climate | 61** | 60 | 64† | 53† | | | | | |
| | Others[5] | | | | | | | | | |

**JBI risk of bias**
Low (0.70 - 1.0)
Moderate (0.50 - 0.70)
High (0.0 - 0.50)

**Effect on outcome variable**
Benefit
No effect
Loss

**Significance level**
** Statistically significant at 5%
* Statistically significant at 10%
† Not reported

## III. Studies conducted in the consumer service sector

| Domain | Intervention type | Absence: Sick days | Absence: Absence rate | Employer economics: ROI[1] | Employer economics: Net benefit | Employer economics: CBR[3] | Employer economics: CER[4] | Employer economics: Other |
|---|---|---|---|---|---|---|---|---|
| **Physical health & fitness** | Exercise programs | | 64† 79 76 | | | | 78† | |
| | Nutrition & weight management | | 64† 79 76 | | | | 78† | 67† |
| | Health monitoring | 93 | | | | | 78† | |
| **Mental health & stress** | Mindfulness & relaxation | | 64† 79 | 41 | 41 | 41 | 41 | 41 |
| | Mental health initiatives | 93 | | 41 | 41 | 41 | 41 | 41 |
| | Policies | | | | | | | |
| **Working atmosphere** | Education & training | 36** | | | | | | |
| | Work climate | 39 | 64† | | | | | |
| | Others[5] | | | | | | | |

**JBI risk of bias**
Low (0.70 - 1.0)
Moderate (0.50 - 0.70)
High (0.0 - 0.50)

**Effect on outcome variable**
Benefit
No effect
Loss

**Significance level**
** Statistically significant at 5%
* Statistically significant at 10%
† Not reported

## IV. Studies conducted in other sectors.

Financial (= Fi), technology (= Tec), telecommunication (= Tel), basic materials (= Ba), and consumer stables (=CS) have been clustered as other sectors. Industry abbreviations are listed in the respective cells.

| | Absence | | | Employer economics | | | | |
|---|---|---|---|---|---|---|---|---|
| Intervention type | Sick days | Absence rate | Others | ROI[1] | Net benefit | CBR[3] | CER[4] | CER[4] |
| Exercise programs | 74 (Ba) | 75 (Fi)† | | 87 (Fi)** | 87 (Fi)** | 87 (Fi)** | 78 (Fi)† 87 (Fi)** | |
| Nutrition & weight management | | 75 (Fi)† | | | | | 78 (Fi)† | |
| Health monitoring | 81 (Tel)** 34 (Tec) 82 (Tel)* | 46 (Fi)** 75 (Fi)† | | 88 (CS)* 69 (Tec)† | 88 (CS)* 69 (Tec)† | 88 (CS)* | 82 (Tel)* 78 (Fi)† | |
| Mindfulness & relaxation | | 75 (Fi)† | | 41 (Fi) | 41 (Fi) | 41 (Fi) | 41 (Fi) | 41 (Fi) |
| Mental health initiatives | | 52 (Tec)** | | 41 (Fi) | 41 (Fi) | 41 (Fi) | 41 (Fi) | 41 (Fi) |
| Policies | | | | | | | | |
| Education & training | | | | 69 (Tec)† | 69 (Tec)† | | | |
| Work climate | | 46 (Fi)** 52 (Tec)** | | 87 (Fi)** | 87 (Fi)** | 87 (Fi)** | 87 (Fi)** | |
| Others[5] | | | | | | | | |

**JBI risk of bias**
Low (0.70 - 1.0)
Moderate (0.50 - 0.70)
High (0.0 - 0.50)

**Effect on outcome variable**
Benefit
No effect
Loss

**Significance level**
** Statistically significant at 5%
* Statistically significant at 10%
† Not reported

## Figure S4. Random-effects model (REML method) for mean differences (MD) in sick days in low risk of bias studies (JBI > 0.7)

| Study | Domain | Industry | MD | 95%-CI | Weight |
|---|---|---|---|---|---|
| (34) De Boer et al. (2004) | P | Consumer Products/Services | -12.00 | | 0.0% |
| (91) Vonderlin et al. - Supervisor (2023) | P, M | Health Care | -9.50 | [-17.99; -1.01] | 0.3% |
| (47) Hengel et al. (2014) | P, M | Industrials | -8.50 | | 0.0% |
| (59) Klasen et al. (2021) | M | Not reported | -6.45 | [-21.37; 8.47] | 0.1% |
| (74) Rantonen et al. - Rehab (2018) | P | Basic Materials | -1.25 | [-15.75; 13.25] | 0.1% |
| (39) Framke et al. (2016) | W | Public sector | -0.49 | | 0.0% |
| (62) Larsen et al. (2019) | W | Health Care | -0.44 | [ -0.95; 0.07] | 95.0% |
| (74) Rantonen et al. - Physio (2018) | P | Basic Materials | 0.25 | [-14.75; 15.25] | 0.1% |
| (93) Zetterberg et al. (2022) | P, M | Public sector; Health Care | 0.98 | [-13.33; 15.29] | 0.1% |
| (91) Vonderlin et al. - Employee (2023) | P, M | Health Care | 1.00 | [ -3.11; 5.11] | 1.5% |
| (89) Viester et al. (2014) | P | Industrials | 1.00 | [ -3.65; 5.65] | 1.1% |
| (80) Stansfeld et al. (2015) | M | Health Care | 2.40 | [ -1.60; 6.40] | 1.5% |
| (74) Rantonen et al. - Advice (2018) | P | Basic Materials | 2.75 | [-13.75; 19.25] | 0.1% |
| **Random effects model** | | | **-0.39** | **[ -0.89; 0.10]** | **100.0%** |

Heterogeneity: $I^2 = 0.0\%$, $\tau^2 = 0$, $p = 0.5397$

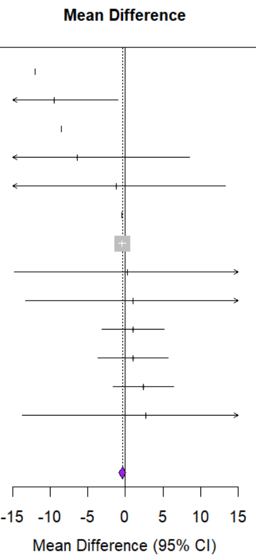


Note: Study numbers correspond to reference number and numbering of Table 4 of the Supplement Material. For the intervention domain, the following abbreviations apply: P = Physical health and fitness; M = Mental health and stress; W = Education and training. Studies that did not report statistical results for the mean difference in sick days were excluded from the random-effects calculation (assigned a weight of 0%) and are displayed for reference purposes only. Some studies report

sick day results for specific sub-groups (e.g., Vonderlin et al. (91), hence they appear multiple times in the forest plot with the respective subgroup designation. The P value at the lower end of the forest diagram refers to the heterogeneity test.

**Figure S5. Funnel plots for primary meta-analyses (low-risk studies only) and all studies including in meta analyses irrespective of JBI rating**

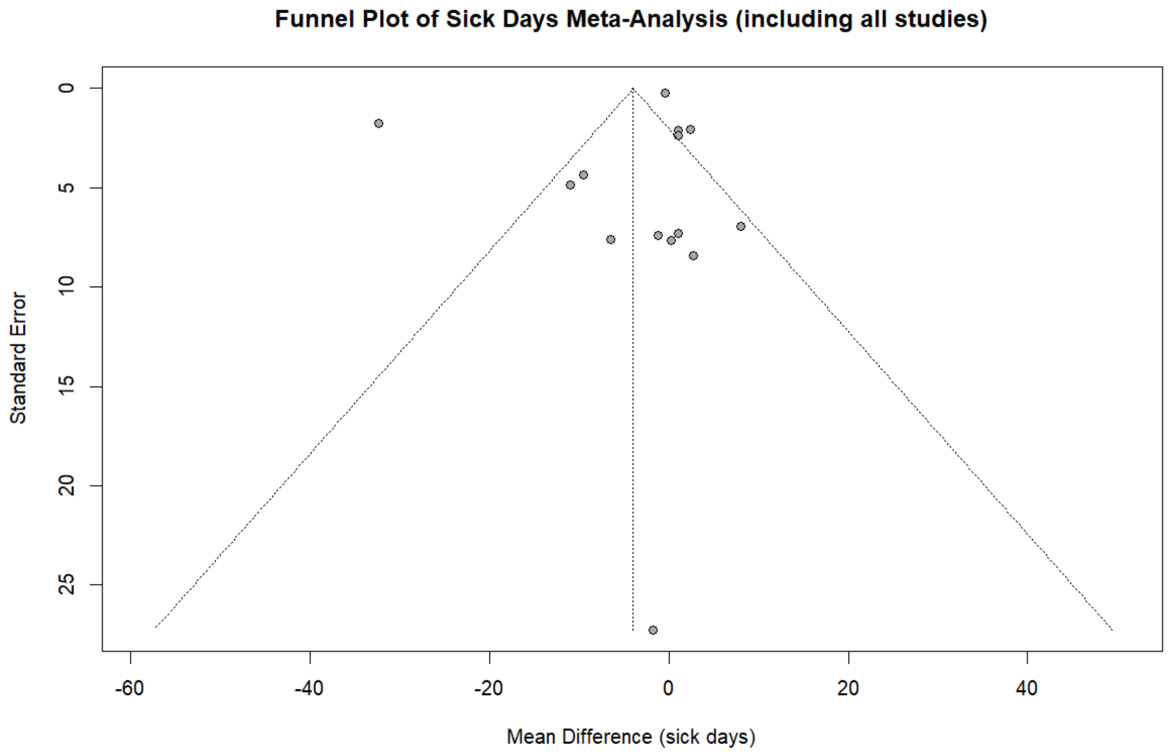


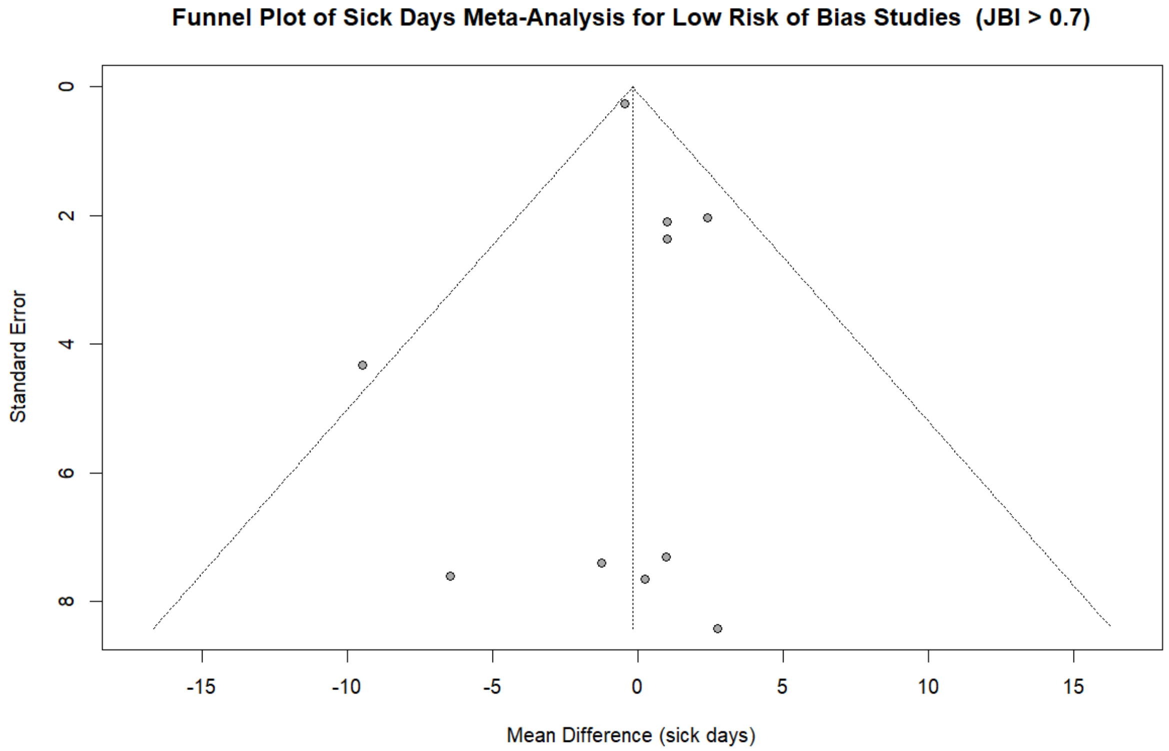

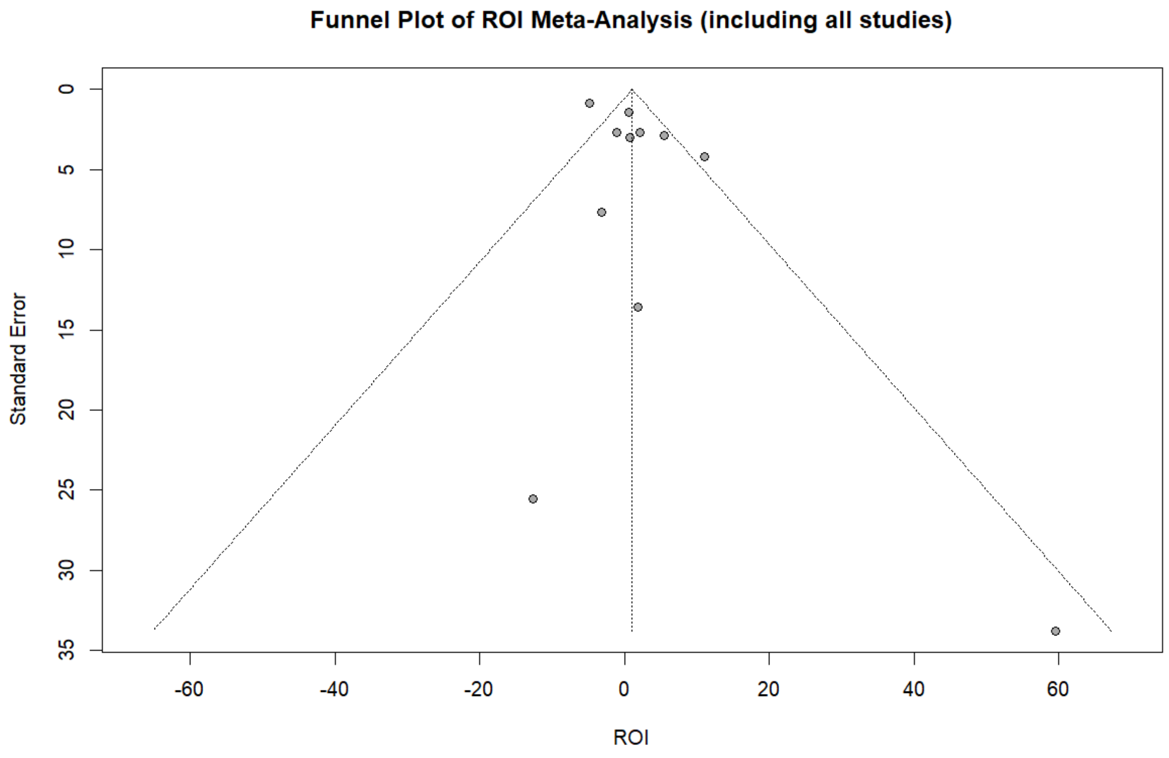
Funnel Plot of ROI Meta-Analysis (including all studies)
Standard Error
0
5
10
15
20
25
30
35
-60
-40
-20
0
20
40
60
ROI

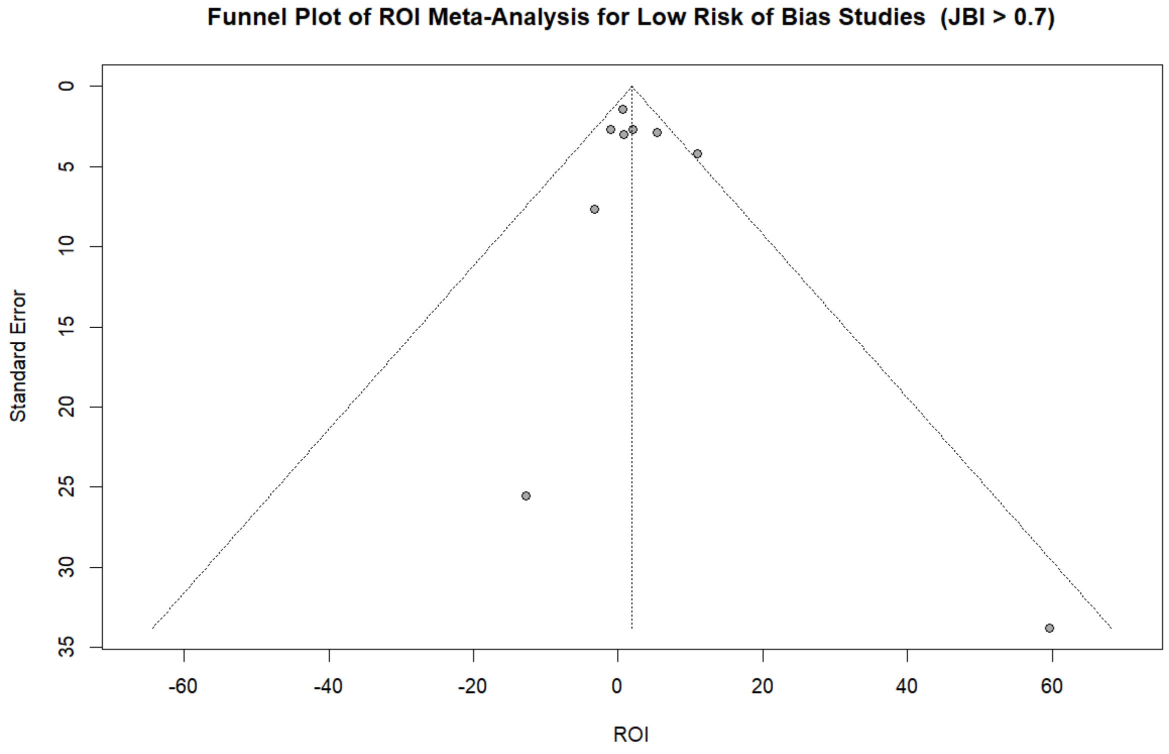
Funnel Plot of ROI Meta-Analysis for Low Risk of Bias Studies (JBI > 0.7)
Standard Error
0
5
10
15
20
25
30
35
-60
-40
-20
0
20
40
60
ROI

## Figure S6. Forest plots of sub-group analyses

Ten subgroup analyses have been conducted to assess the impact of OHIs on sick days and return on investment. The results of these analyses are presented below.

### 1. Subgroup analysis: All studies (sick days)

| Study | Domain | Bias | MD | 95%-CI | Weight |
|---|---|---|---|---|---|
| (61) Krampen (2010) | P, M, W | High | -32.40 | [-35.88; -28.92] | 9.2% |
| (34) De Boer et al. (2004) | P | Low | -12.00 | | 0.0% |
| (82) Taimela et al. (2009) | P | Moderate | -11.00 | [-20.00; -1.00] | 7.8% |
| (91) Vonderlin et al. - Supervisor (2023) | P, M | Low | -9.50 | [-17.99; -1.01] | 8.1% |
| (47) Hengel et al. (2014) | P, M | Low | -8.50 | | 0.0% |
| (59) Klasen et al. (2021) | M | Low | -6.45 | [-21.37; 8.47] | 6.1% |
| (36) Duijts et al. (2008) | W | Moderate | -2.53 | | 0.0% |
| (84) Tveito and Erikson (2008) | P, M | Moderate | -1.80 | [-51.60; 55.20] | 1.2% |
| (74) Rantonen et al. - Rehab (2018) | P | Low | -1.25 | [-15.75; 13.25] | 6.2% |
| (39) Framke et al. (2016) | W | Low | -0.49 | | 0.0% |
| (62) Larsen et al. (2019) | W | Low | -0.44 | [ -0.95; 0.07] | 9.5% |
| (74) Rantonen et al. - Physio (2018) | P | Low | 0.25 | [-14.75; 15.25] | 6.1% |
| (93) Zetterberg et al. (2022) | P, M | Low | 0.98 | [-13.33; 15.29] | 6.3% |
| (91) Vonderlin et al. - Employee (2023) | P, M | Low | 1.00 | [ -3.11; 5.11] | 9.1% |
| (89) Viester et al. (2014) | P | Low | 1.00 | [ -3.65; 5.65] | 9.0% |
| (80) Stansfeld et al. (2015) | M | Low | 2.40 | [ -1.60; 6.40] | 9.1% |
| (74) Rantonen et al. - Advice (2018) | P | Low | 2.75 | [-13.75; 19.25] | 5.7% |
| (33) Brox and Frøystein (2005) | P, M | Moderate | 8.06 | [ -5.56; 21.67] | 6.5% |
| **Random effects model** | | | **-3.99** | **[-10.18; 2.21]** | **100.0%** |

Heterogeneity: $I^2 = 96.1\%$, $\tau^2 = 105.1069$, $p < 0.0001$

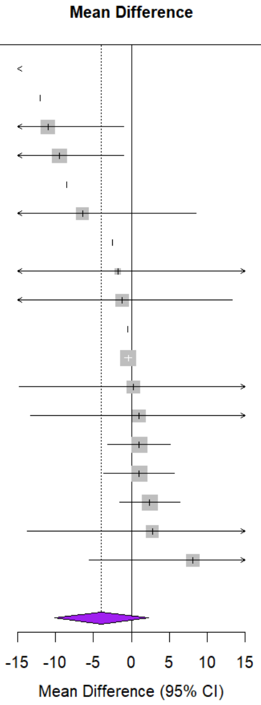


### 2. Subgroup analysis: All studies (ROI)

| Study | Domain | Bias | ROI | 95%-CI | Weight | Follow-up |
|---|---|---|---|---|---|---|
| (87) Van Dongen et al. - Physical (2017) | P | Low | -12.68 | [-65.64; 34.42] | 0.4% | 12 |
| (88) Van Holland et al. (2017) | M | High | -4.87 | [ -6.70; -3.45] | 18.5% | 36 |
| (86) Van Dongen et al. (2013) | P | Low | -3.21 | [-17.87; 12.19] | 3.8% | 12 |
| (87) Van Dongen et al. - Social (2017) | W | Low | -1.04 | [ -6.31; 4.21] | 12.9% | 12 |
| (58) Keus van de Poll et al. (2020) | W | Low | -0.12 | | 0.0% | 12 |
| (49) Herman et al. (2008) | P | Moderate | 0.08 | | 0.0% | 6 |
| (28) Baker et al. (2008) | P | High | 0.17 | | 0.0% | 12 |
| (37) Ebert et al. (2018) | M, W | Low | 0.61 | [ -2.20; 3.50] | 16.9% | 6 |
| (45) Gubler et al. (2017) | P | Low | 0.76 | | 0.0% | 36 |
| (40) Freund et al. (2024) | M, W | Low | 0.77 | [ -5.03; 6.72] | 11.9% | 6 |
| (42) Goetzel et al. (2014) | P, M | High | 1.03 | | 0.0% | 12 |
| (35) Dement et al. (2015) | P | Low | 1.53 | | 0.0% | 72 |
| (51) Iijima et al. (2013) | M | High | 1.55 | | 0.0% | 12 |
| (63) Lerner et al. (2020) | M | Low | 1.60 | | 0.0% | 23 |
| (41) Geraedts et al. (2015) | M | Moderate | 1.78 | [-24.66; 28.63] | 1.4% | 12 |
| (83) Thiart et al. (2016) | M | Low | 2.08 | [ -2.97; 7.44] | 13.0% | 6 |
| (38) Elson et al. (2019) | M | Moderate | 2.34 | | 0.0% | 12 |
| (48) Hengel et al. (2014) | P, M | Low | 5.44 | [ 0.41; 11.79] | 12.2% | 12 |
| (70) Noben et al. (2015) | M | Low | 11.00 | [ 2.82; 19.18] | 8.7% | 6 |
| (92) Wijnen et al. (2019) | M | Low | 59.62 | [ -6.58; 125.82] | 0.2% | 12 |
| **Random effects model** | | | **1.05** | **[ -2.21; 4.30]** | **100.0%** | |

Heterogeneity: $I^2 = 74.0\%$, $\tau^2 = 14.2240$, $p < 0.0001$

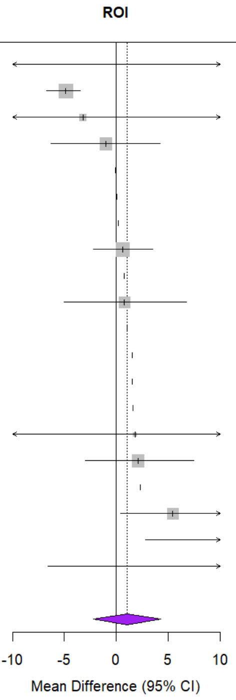

## 3. Subgroup analysis: Excluding high risk of bias (sick days)

| Study | Domain | Industry | MD | 95%-CI | Weight |
|---|---|---|---|---|---|
| (34) De Boer et al. (2004) | P | Consumer Products/Services | -12.00 | | 0.0% |
| (82) Taimela et al. (2009) | P | Industrials; Telecomm. | -11.00 | [-20.00; -1.00] | 6.9% |
| (91) Vonderlin et al. - Supervisor (2023) | P, M | Health Care | -9.50 | [-17.99; -1.01] | 8.0% |
| (47) Hengel et al. (2014) | P, M | Industrials | -8.50 | | 0.0% |
| (59) Klasen et al. (2021) | M | Not reported | -6.45 | [-21.37; 8.47] | 3.6% |
| (36) Duijts et al. (2008) | W | Health Care; Public | -2.53 | | 0.0% |
| (84) Tveito and Erikson (2008) | P, M | Health Care | -1.80 | [-51.60; 55.20] | 0.3% |
| (74) Rantonen et al. - Rehab (2018) | P | Basic Materials | -1.25 | [-15.75; 13.25] | 3.7% |
| (39) Framke et al. (2016) | W | Public sector | -0.49 | | 0.0% |
| (62) Larsen et al. (2019) | W | Health Care | -0.44 | [ -0.95; 0.07] | 19.7% |
| (74) Rantonen et al. - Physio (2018) | P | Basic Materials | 0.25 | [-14.75; 15.25] | 3.5% |
| (93) Zetterberg et al. (2022) | P, M | Public sector; Health Care | 0.98 | [-13.33; 15.29] | 3.8% |
| (91) Vonderlin et al. - Employee (2023) | P, M | Health Care | 1.00 | [ -3.11; 5.11] | 14.7% |
| (89) Viester et al. (2014) | P | Industrials | 1.00 | [ -3.65; 5.65] | 13.7% |
| (80) Stansfeld et al. (2015) | M | Health Care | 2.40 | [ -1.60; 6.40] | 14.9% |
| (74) Rantonen et al. - Advice (2018) | P | Basic Materials | 2.75 | [-13.75; 19.25] | 3.0% |
| (33) Brox and Frøystein (2005) | P, M | Health Care | 8.06 | [ -5.56; 21.67] | 4.1% |
| **Random effects model** | | | **-0.79** | **[ -3.89; 2.32]** | **100.0%** |

Heterogeneity: $I^2$ = 15.6%, $\tau^2$ = 12.6567, $p$ = 0.2874

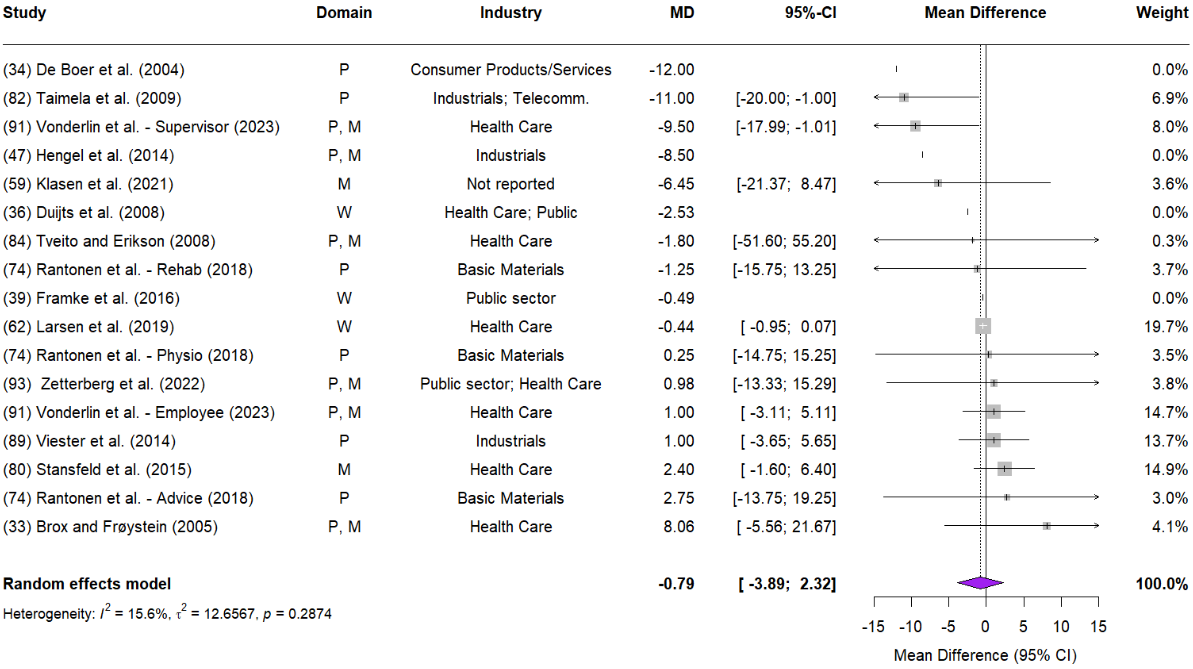


## 4. Subgroup analysis: Excluding high risk of bias (ROI)

| Study | Domain | Industry | ROI | 95%-CI | Weight | Timeframe |
|---|---|---|---|---|---|---|
| (87) Van Dongen et al. - Physical (2017) | P | Financials | -12.68 | [-65.64; 34.42] | 0.2% | 12 |
| (86) Van Dongen et al. (2013) | P | Health Care | -3.21 | [-17.87; 12.19] | 2.1% | 12 |
| (87) Van Dongen et al. - Social (2017) | W | Financials | -1.04 | [ -6.31; 4.21] | 14.7% | 12 |
| (58) Keus van de Poll et al. (2020) | W | Various | -0.12 | | 0.0% | 12 |
| (49) Herman et al. (2008) | P | Consumer Discretionary | 0.08 | | 0.0% | 6 |
| (37) Ebert et al. (2018) | M, W | Not reported | 0.61 | [ -2.20; 3.50] | 35.3% | 6 |
| (45) Gubler et al. (2017) | P | Consumer Discretionary | 0.76 | | 0.0% | 36 |
| (40) Freund et al. (2024) | M, W | Diverse | 0.77 | [ -5.03; 6.72] | 12.2% | 6 |
| (35) Dement et al. (2015) | P | Health Care; Public Sector | 1.53 | | 0.0% | 72 |
| (63) Lerner et al. (2020) | M | Not reported | 1.60 | | 0.0% | 23 |
| (41) Geraedts et al. (2015) | M | Financials; Public sector; Industrials | 1.78 | [-24.66; 28.63] | 0.7% | 12 |
| (83) Thiart et al. (2016) | M | Public sector | 2.08 | [ -2.97; 7.44] | 15.0% | 6 |
| (38) Elson et al. (2019) | M | Public sector | 2.34 | | 0.0% | 12 |
| (48) Hengel et al. (2014) | P, M | Industrials | 5.44 | [ 0.41; 11.79] | 12.9% | 12 |
| (70) Noben et al. (2015) | M | Health Care | 11.00 | [ 2.82; 19.18] | 6.8% | 6 |
| (92) Wijnen et al. (2019) | M | Health Care | 59.62 | [ -6.58; 125.82] | 0.1% | 12 |
| **Random effects model** | | | **1.90** | **[ -0.32; 4.12]** | **100.0%** | |

Heterogeneity: $I^2$ = 25.1%, $\tau^2$ = 1.5230, $p$ = 0.2126

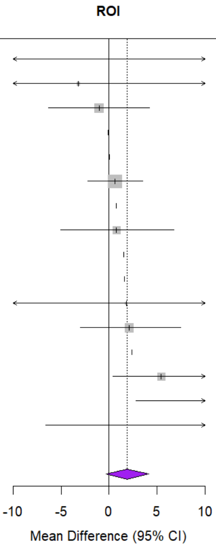

## 5. Subgroup analysis: Low risk of bias by intervention domain (sick days)

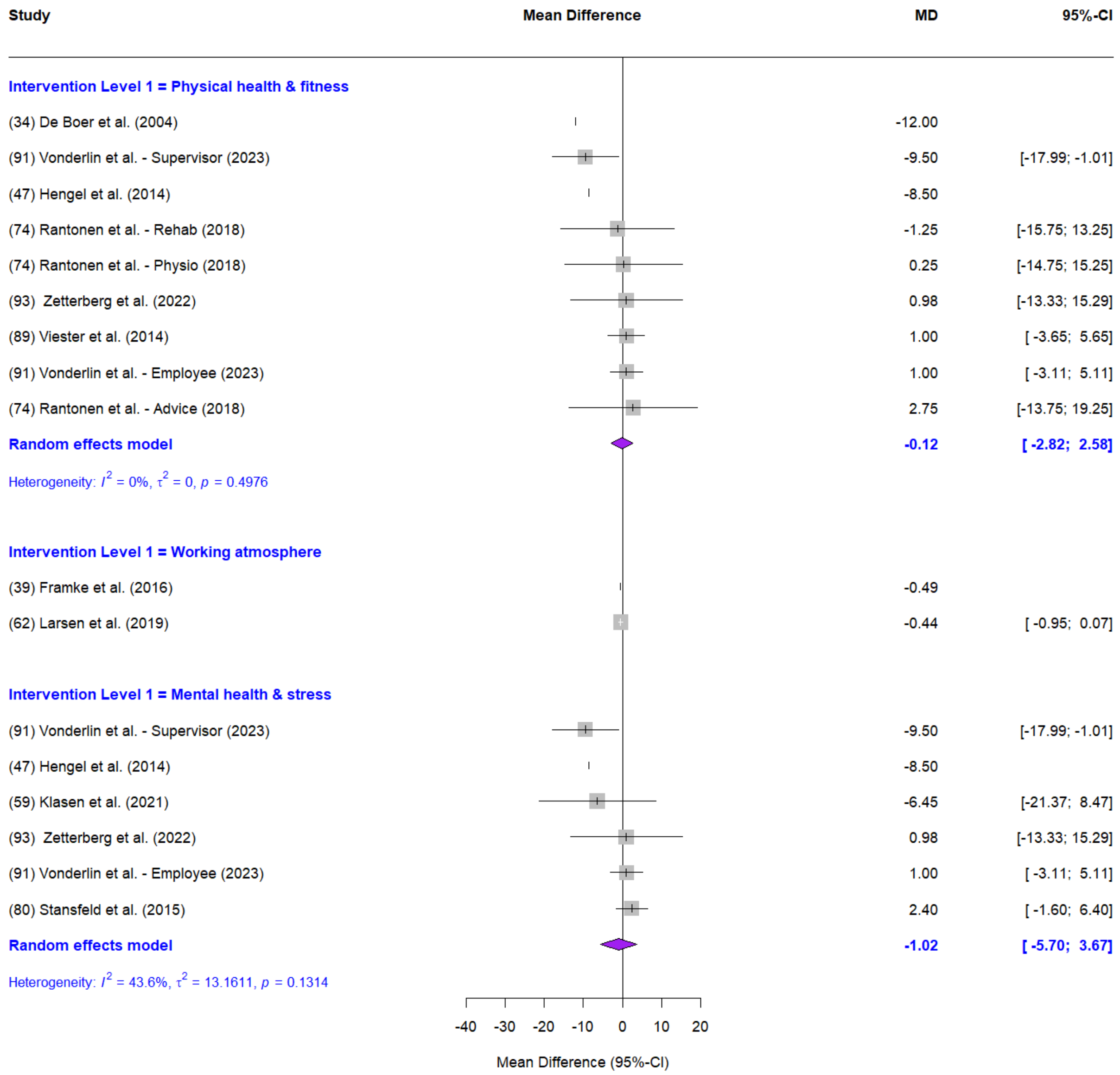

## 6. Subgroup analysis: Low risk of bias by intervention domain (ROI)

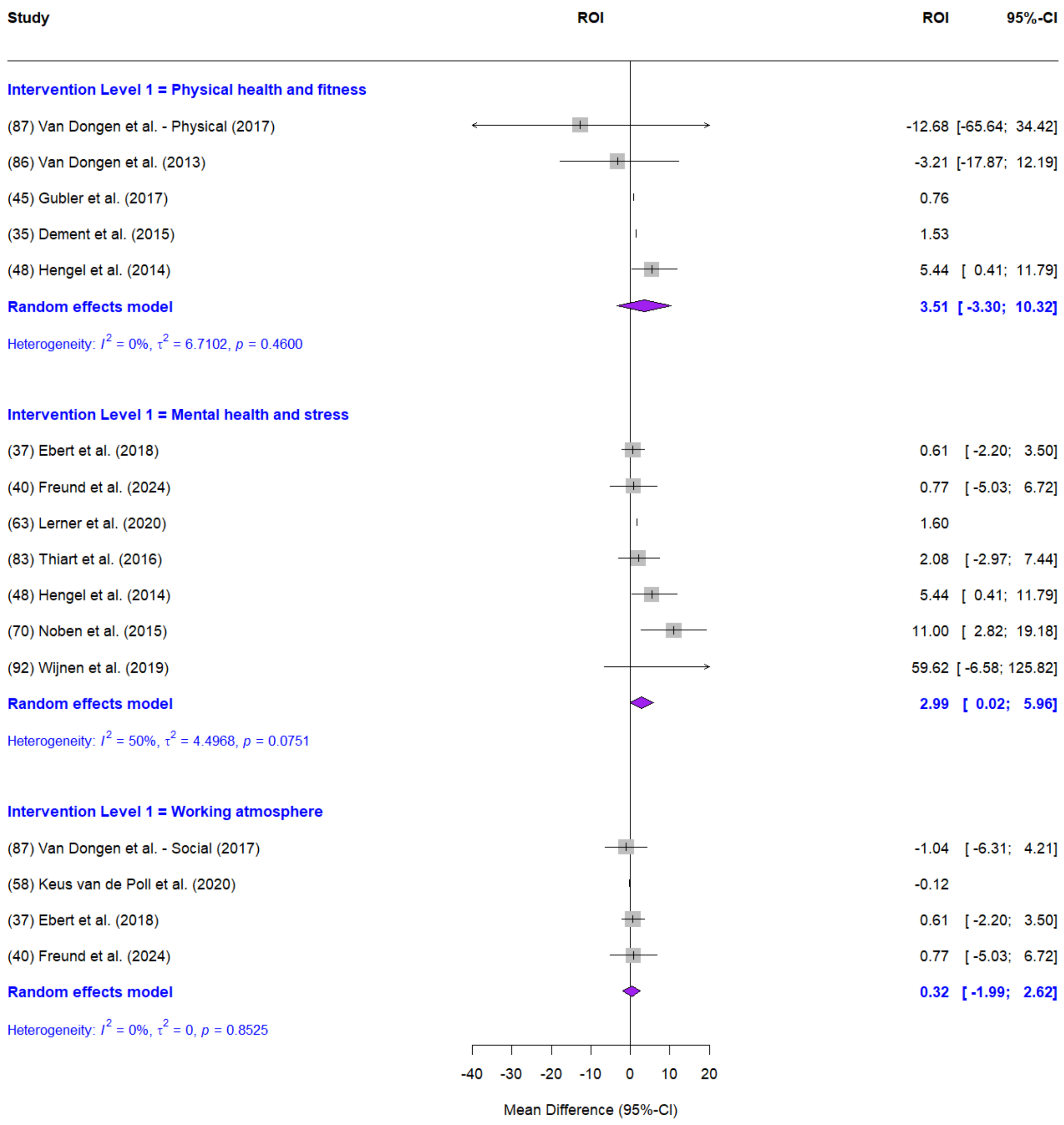

## 7. Subgroup analysis: Low risk of bias by intervention type (sick days)

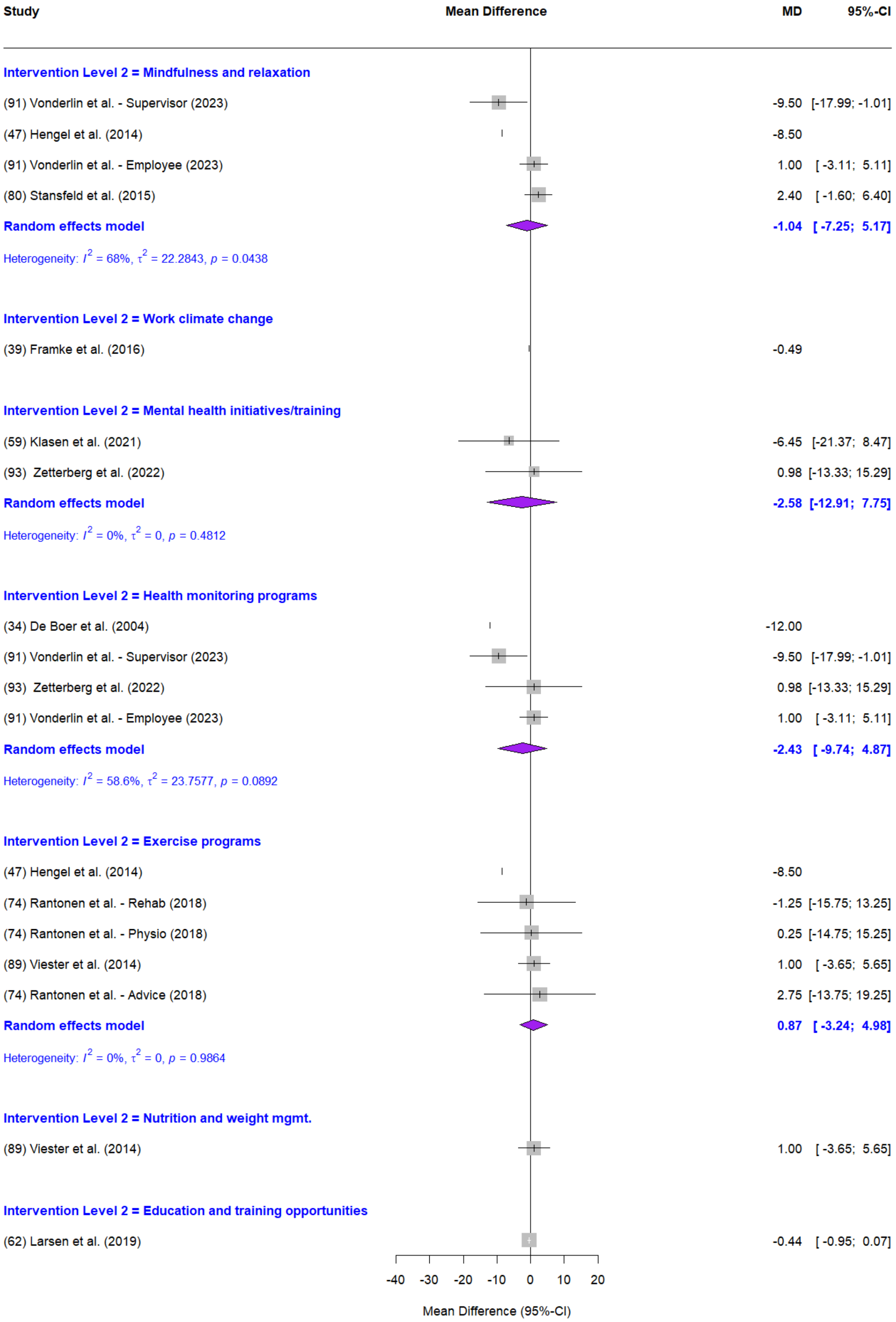

# 8. Subgroup analysis: Low risk of bias by intervention type (ROI)

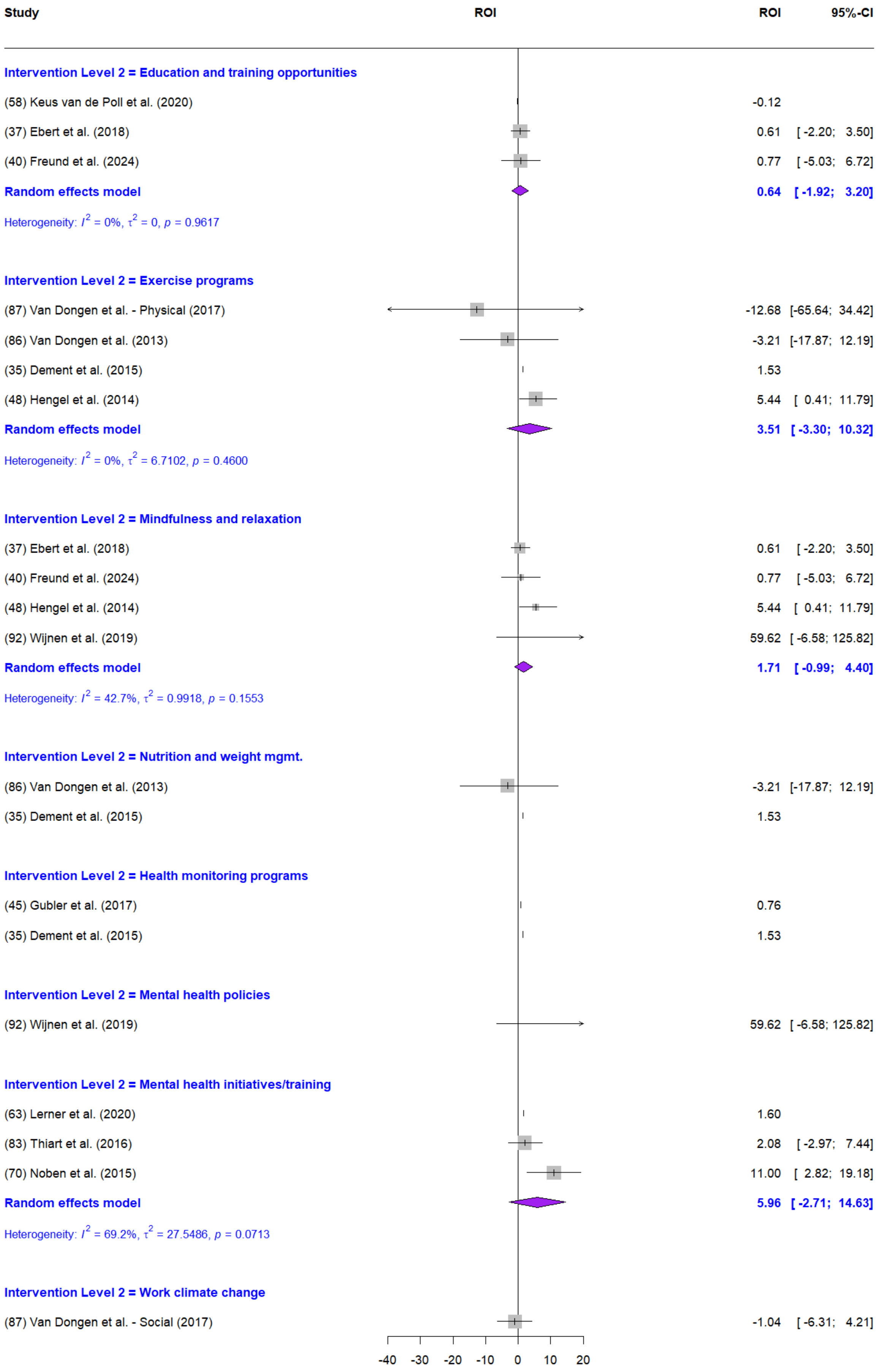

## 9. Subgroup analysis: Low risk of bias by industry type (sick days)

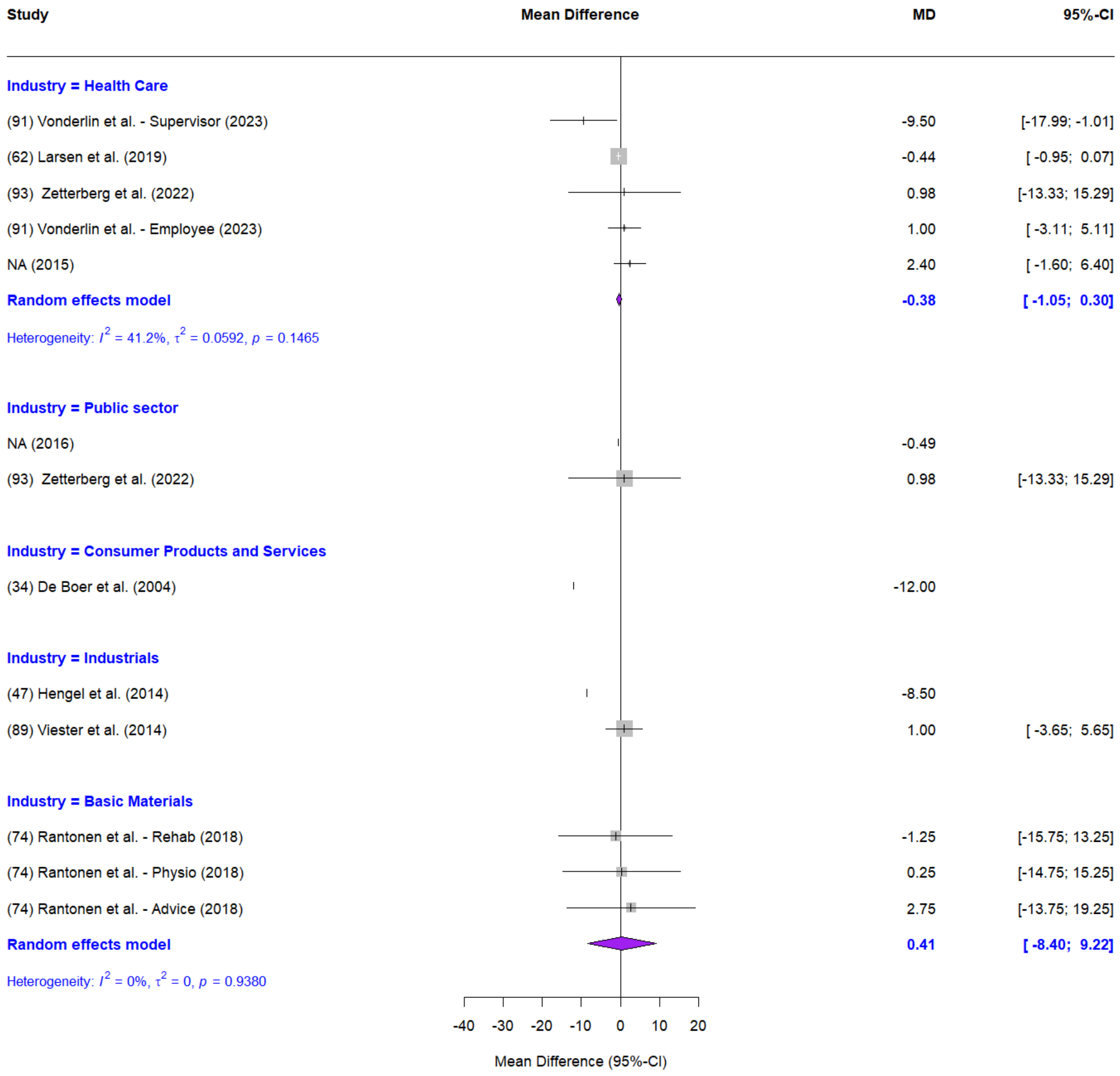

## 10. Subgroup analysis: Low risk of bias by industry type (ROI)

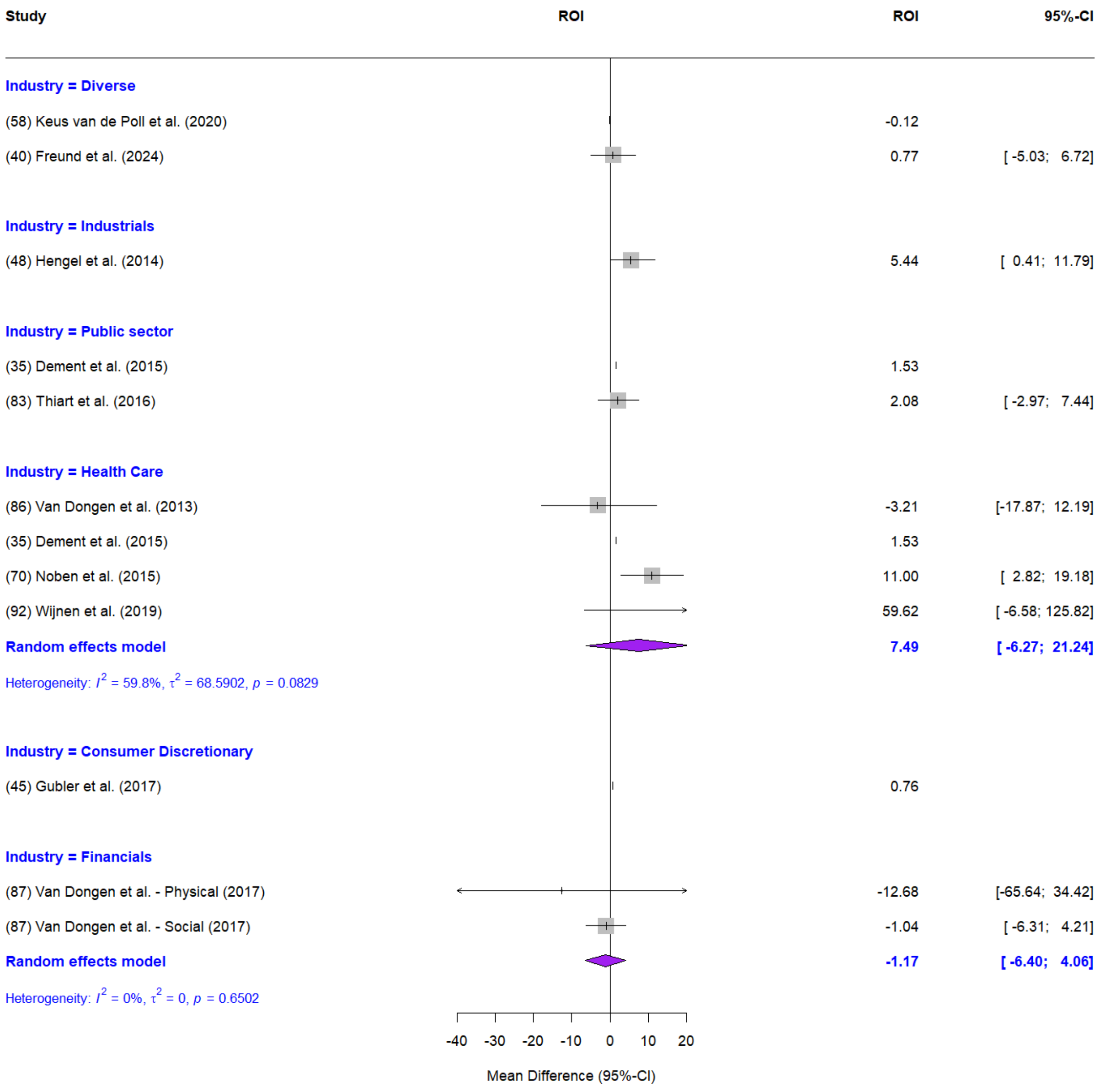

## 11. Subgroup analysis: Low risk of bias by delivery format (sick days)

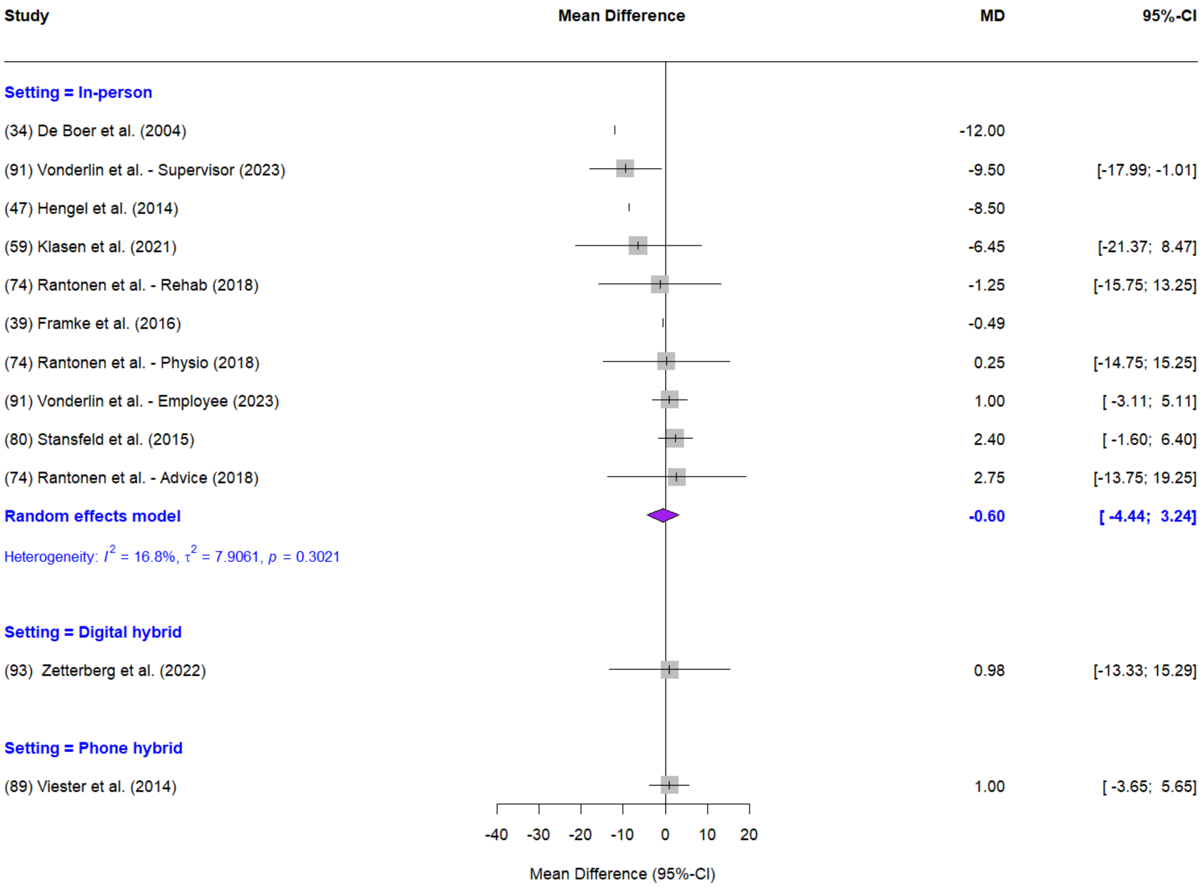

## 12. Subgroup analysis: Low risk of bias by delivery format (ROI)

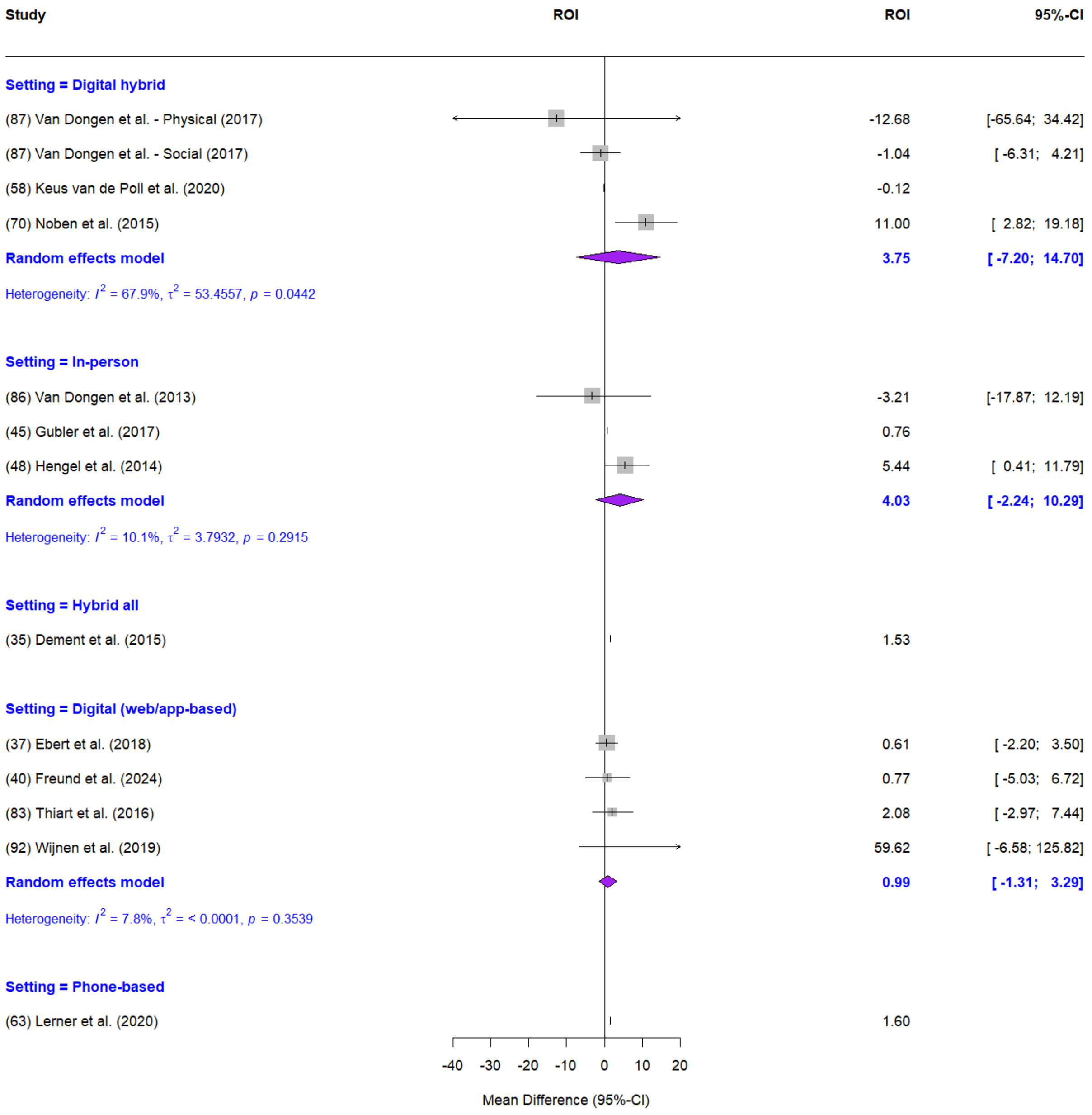

Supplement: Supplementary material [file SJWEH-52-79-S001.pdf]
